# National, regional, and global trends in adult overweight and obesity prevalences

**DOI:** 10.1186/1478-7954-10-22

**Published:** 2012-11-20

**Authors:** Gretchen A Stevens, Gitanjali M Singh, Yuan Lu, Goodarz Danaei, John K Lin, Mariel M Finucane, Adil N Bahalim, Russell K McIntire, Hialy R Gutierrez, Melanie Cowan, Christopher J Paciorek, Farshad Farzadfar, Leanne Riley, Majid Ezzati

**Affiliations:** 1 Department of Health Statistics and Information Systems, World Health Organization, Geneva, Switzerland; 2 Department of Global Health and Population, Harvard School of Public Health, Boston, USA; 3 Department of Epidemiology, Harvard School of Public Health, Boston, USA; 4 Independent consultant, Geneva, Switzerland; 5 Department of Chronic Diseases and Health Promotion, World Health Organization, Geneva, Switzerland; 6 Department of Statistics, University of California, Berkeley, USA; 7 Noncommunicable Diseases Research Centre, Endocrinology and Metabolism Research Institute, Tehran University of Medical Sciences, Tehran, Iran; 8 Department of Epidemiology and Biostatistics, MRC-HPA Center for Environment and Health, School of Public Health, Imperial College London, London, UK

**Keywords:** Overweight, Obesity, Prevalence, Population health, Risk transition, Global health, Noncommunicable diseases

## Abstract

**Background:**

Overweight and obesity prevalence are commonly used for public and policy communication of the extent of the obesity epidemic, yet comparable estimates of trends in overweight and obesity prevalence by country are not available.

**Methods:**

We estimated trends between 1980 and 2008 in overweight and obesity prevalence and their uncertainty for adults 20 years of age and older in 199 countries and territories. Data were from a previous study, which used a Bayesian hierarchical model to estimate mean body mass index (BMI) based on published and unpublished health examination surveys and epidemiologic studies. Here, we used the estimated mean BMIs in a regression model to predict overweight and obesity prevalence by age, country, year, and sex. The uncertainty of the estimates included both those of the Bayesian hierarchical model and the uncertainty due to cross-walking from mean BMI to overweight and obesity prevalence.

**Results:**

The global age-standardized prevalence of obesity nearly doubled from 6.4% (95% uncertainty interval 5.7-7.2%) in 1980 to 12.0% (11.5-12.5%) in 2008. Half of this rise occurred in the 20 years between 1980 and 2000, and half occurred in the 8 years between 2000 and 2008. The age-standardized prevalence of overweight increased from 24.6% (22.7-26.7%) to 34.4% (33.2-35.5%) during the same 28-year period. In 2008, female obesity prevalence ranged from 1.4% (0.7-2.2%) in Bangladesh and 1.5% (0.9-2.4%) in Madagascar to 70.4% (61.9-78.9%) in Tonga and 74.8% (66.7-82.1%) in Nauru. Male obesity was below 1% in Bangladesh, Democratic Republic of the Congo, and Ethiopia, and was highest in Cook Islands (60.1%, 52.6-67.6%) and Nauru (67.9%, 60.5-75.0%).

**Conclusions:**

Globally, the prevalence of overweight and obesity has increased since 1980, and the increase has accelerated. Although obesity increased in most countries, levels and trends varied substantially. These data on trends in overweight and obesity may be used to set targets for obesity prevalence as requested at the United Nations high-level meeting on Prevention and Control of NCDs.

## Background

Excess body weight is an important risk factor for mortality and morbidity from cardiovascular diseases, diabetes, cancers, and musculoskeletal disorders, causing nearly three million annual deaths worldwide [1-5]. In previous work, we used a systematic analysis of data from population-based health examination surveys and epidemiologic studies to estimate trends in mean body mass index (BMI) between 1980 and 2008 in 199 countries and territories in 21 regions [6]. The results demonstrated the extent of the rise in BMI, and its similarities and differences, across countries and regions. This work, however, provided limited information on the prevalences of overweight and obesity, at the regional level and for two years, 1980 and 2008, only. Data on the prevalence of overweight and obesity by country and their trends are needed for two reasons: first, although epidemiologic studies have shown that ischemic heart disease, ischemic stroke, and diabetes are linearly associated with measures of excess body weight from low levels, some other outcomes, including hemorrhagic stroke, may have thresholds at higher levels [1,3,5]. Second, prevalence estimates are commonly used for public and policy communication of the extent of the obesity epidemic; hence there is great demand for such information. In this paper, we advance our previous work by estimating trends in overweight and obesity for all countries and regions.

## Methods

### Overview

We estimated 1980 to 2008 trends in the prevalence of overweight, defined as BMI ≥ 25 kg/m^2^, and obesity, defined as BMI ≥ 30 kg/m^2^, by sex, for 199 countries and territories. We estimated prevalences using the relationship between prevalence and mean BMI, applied to estimates of mean BMI from a previously published systematic analysis of health examination surveys and epidemiological studies [6]. We also estimated the uncertainties of prevalence estimates, accounting for the uncertainties of mean BMI and those of converting from mean to prevalence.

### Mean BMI by sex, age group, country, and year

Mean BMI was from a previous systematic analysis of population-based data, by sex and age group, for 199 countries and territories, described in detail elsewhere [6]. In summary, we reviewed and accessed published and unpublished health examination surveys and epidemiologic studies to collate comprehensive data on BMI, screened for being representative of populations studied. This review led to a total of 960 country-years of data, with 9.1 million participants. Of these, 369 were nationally representative. We applied a Bayesian statistical model that systematically addressed missing data, non-linear time trends and age associations, national vs. subnational and community representativeness of data, and data that were from only rural or urban populations. Using these data and methods, we estimated mean BMI trends and their uncertainties by sex, country, and age group for adults ≥ 20 years.

The uncertainty of the estimated mean BMI accounted for sampling uncertainty in the original data sources; uncertainty associated with interannual fluctuations in national data, due to unmeasured study design factors, or because some surveys did not have sample weights; uncertainty associated with data sources that were not national, due to variation across communities in each country; uncertainty associated with converting between different metrics of excess body weight; and uncertainty due to using a model to estimate mean BMI by age group, country, and year when data were missing. As described elsewhere, we fit the Bayesian model using the Markov chain Monte Carlo (MCMC) algorithm and obtained samples from the posterior distribution of model parameters, reflecting the above sources of uncertainty, which were in turn used to obtain samples from the posterior distribution of mean BMI [6,7]. Five thousand posterior draws of mean BMI by age, sex, country, and year were generated.

### Estimating the prevalences of overweight and obesity from mean BMI

**Figure 1:**
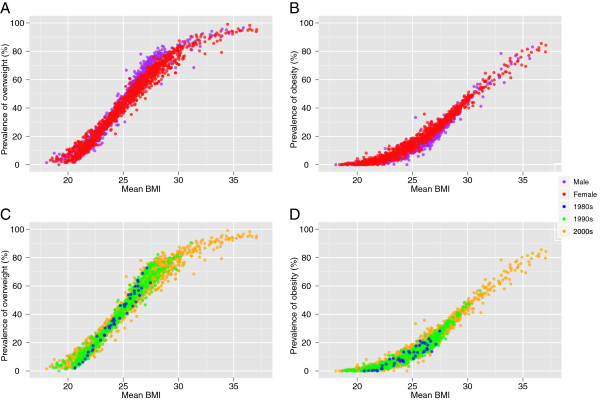
**Mean BMI vs. prevalence of overweight (BMI ≥ 25 kg/m**^**2**^**), (A) by gender and (C) by decade; and mean BMI vs. prevalence of obesity (BMI ≥ 30 kg/m**^**2**^**), (B) by gender and (D) by decade.** Data are from 243 health examination surveys, by age and sex.

We developed regressions to estimate overweight and obesity prevalences from mean BMI. The dependent variable in each regression was the logit of the prevalence; the independent variables were mean BMI, age (mid-year of age group), sex, year of survey, and whether the country was high income. We used logit transformation to restrict the estimated prevalences between 0 and 1. We modeled the relationship using a cubic spline of mean BMI because the association was nonlinear (Figure 1); see below for sensitivity to this functional form. We included year in the regressions to reflect changes in the mean-prevalence relationship over time. Ideally, we would have used a very flexible function of year. However, fewer sources with individual-level data were available in the terminal years of the analysis, with data for males virtually absent in the 1980s. To avoid potentially spurious effects when extrapolating year effects to years when data were very sparse, we set the year variable to 0 prior to 1993. For other years, we used the difference between two splines, one with knots in 1993, 1998, and 2003, and the other with knots in 1998, 2003, and 2008, the last year of the analysis. This difference increases linearly with year between 1998 and 2003, and is attenuated in the tails, when data are most sparse (see below for sensitivity of results to the inclusion of year and choice of year function). Our final model was:

(1) $$\text{Logit}\left(\text{Pr}\right)=\beta_{0}+\beta_{1}\text{m}+\beta_{2}{\text{m}}^{2}+\beta_{3}{\text{m}}_{\text{s}1}+\beta_{4}{\text{m}}_{\text{s}2}+\beta_{5}{\text{m}}_{\text{s}3}+\beta_{6}{\text{m}}_{\text{s}4}+\beta_{7}\text{a}+\beta_{8}\text{s}+\beta_{9}\text{y}+\beta_{10}\text{h}+\beta_{11}\text{y}*\text{h}$$

where *Pr* is prevalence of overweight/obesity, *m* is mean BMI, *m*_*s1*_*– m*_*s4*_ are BMI spline segments, *a* is the midpoint of age for each age group, *s* is an indicator variable taking the value 0 for males and 1 for females, *y* is the smooth function of year described above, and *h* is an indicator variable taking the value 1 for high-income countries and 0 for all other countries. To fit the “cross-walking” regressions, we used mean BMI, and prevalence of overweight and obesity from 243 population-representative health examination surveys for which we had access to individual-level data (generating 1884 age-sex observations). We used sample weights when analyzing survey data. We fit the model using ordinary least squares regression, and list the estimated β values in Table 1.

**Table 1:** Regression coefficients used to predict the prevalence of overweight/obesity from mean BMI

**Predictor**	**BMI ≥ 25 kg/m2 (n=1883)**^**b**^	**BMI ≥ 30 kg/m2 (n=1857)**^**b**^
Constant	-24.9 (-27.0, -22.8)	-33.9 (-36.9, -31.0)
Cube of first spline segment (knot at BMI of 21.3)	-.0000423 (-.000118, .0000334)	-.0000632 (-.0002191, .0000927)
Cube of second spline segment (knot at BMI of 25.1)	-.00522 (-.00680, -.00365)	-.00619 (-.00947, -.00291)
Cube of third spline segment (knot at BMI of 28.9)	-.00490 (-.00735, -.00245)	-.00437 (-.00611, -.00263)
Cube of last spline segment	.00168 (.00038, .00298)	.00316 (.00222, .00410)
Square of mean BMI	-.0182 (-.0220, -.0144)	-.0280 (-.0327, -.0233)
Mean BMI	1.46 (1.27, 1.65)	1.98 (1.71, 2.25)
High-income country	.0077 (-.0287, .0442)	-.113 (-.181, -.0456)
Age (midpoint of age category)	.00567 (.00499, .00635)	.00456 (.00335, .00577)
Year of survey ^a^	.00934 (.00492, .0138)	.0236 (.0157, .0314)
Female sex	.91 (.62, 1.19)	1.01 (.575, 1.45)
Sex * mean BMI	-.0405 (-.0517, -.0292)	-.0294 (-.0455, -.0133)
County income category * year of survey ^a^	-.0120 (-.0194, -.0047)	-.00128 (-.0132, .0106)
R^2^	0.97	0.92

* denotes statistical interaction.

^a^ See methods for further details on how year of survey was used.

^b^ 1884 age-sex groups provided mean and prevalence data with sufficient sample size, but those with prevalence zero were not used in the above regression because the logit(0) is not defined.

The uncertainty of the estimated prevalences included those of mean BMI from the Bayesian model, described elsewhere [6,7] and summarized above, and the uncertainty associated with converting mean to prevalence. For each of 5,000 draws of mean BMI (which comprises mean BMI values for every age-sex-country unit), we sampled a set of regression coefficients from the fitted regression described above, taking into account the covariance of the regression coefficients. We also sampled from the error term of the fitted regression. We then used the sampled regression coefficients and error term corresponding to each draw to calculate the prevalence of overweight/obesity, resulting in 5,000 predictions for each age, sex, country, and year unit. We calculated uncertainty as the 2.5 and 97.5 percentiles of the distribution of predicted prevalences.

We tested the sensitivity of our model to our treatment of BMI and of year, and found that the model was not sensitive to these choices. If we include a linear year term in equation (1), the average relative difference between country predicted values using the two models is 0.6% (overweight model) and 6.0% (obesity model) prior to 1993, and 0.3% (overweight model) and 0.7% (obesity model) from 1993 onward. After excluding a year term from the model altogether, the average relative difference between country predicted values using the two models is 2.6% (overweight model) and 10.0% (obesity model) prior to 1993, and 0.7% (overweight model) and 2.2% (obesity model) from 1993 onward. Likewise, treating mean BMI as a quadratic function had only a small average effect on predictions – the average relative difference of country predictions was 0.2% for overweight and 0.7% for obesity – but residual plots indicated that the predictions for very high and very low mean BMIs were biased.

For each analysis year, country, and sex, we calculated age-standardized prevalences of overweight and obesity using the World Health Organization (WHO) standard population [8], with age-standardization done for each draw. We report the linear trends in age-standardized prevalence over the 28 years of analysis. To estimate trends, for each posterior draw, country, and sex, we regressed age-standardized prevalence of overweight and of obesity on year. We calculated uncertainty of the estimated trends as the 2.5 and 97.5 percentiles of the regression coefficients across 5,000 posterior draws. We also report the posterior probability (pp) that an estimated increase or decrease corresponds to a truly increasing or decreasing BMI trend, calculated as the proportion of 5,000 regression coefficients that have the same sign as the mean regression coefficient. Posterior probability would be 0.50 in a country or region in which an increase is statistically indistinguishable from a decrease, and a larger posterior probability indicates more certainty.

## Results

The fitted regression equations between prevalence and mean population BMI had an R^2^ of 0.97 for overweight and 0.92 for obesity (Figure 1; Table 1). Overweight and obesity prevalences increased over time for any mean population BMI, indicating that above and beyond increasing mean BMI, there is a widening of the BMI distributions. After accounting for mean BMI and other predictors, women had higher prevalence of obesity, an effect that was stronger at low mean BMIs. Our predicted age-standardized prevalences of overweight ranged from 2.6% in Vietnamese women in 1980 to 93.6% in Nauru men in 2008, and of obesity from 0.3% in Vietnamese men in 1980 to 74.8% in Nauru women in 2008 (Additional file 1).

Globally, the age-standardized prevalence of overweight increased from 24.6% (95% uncertainty interval 22.7-26.7%) in 1980 to 34.4% (33.2-35.5%) in 2008. The prevalence of obesity increased from 6.4% (5.7-7.2%) in 1980 to 12.0% (11.5-12.5%) in 2008. Half of this rise (2.8 percentage points absolute increase in prevalence) occurred in the twenty years between 1980 and 2000, when the global prevalence of obesity was 9.3% (9.1-9.6%); the other half occurred in the 8 years between 2000 and 2008. In absolute numbers, this represents an increase from 572 (527–621) million overweight adults in 1980 to 1.46 (1.41-1.51) billion in 2008. Of these, 508 (486–530) million were obese in 2008.

**Figure 2:**
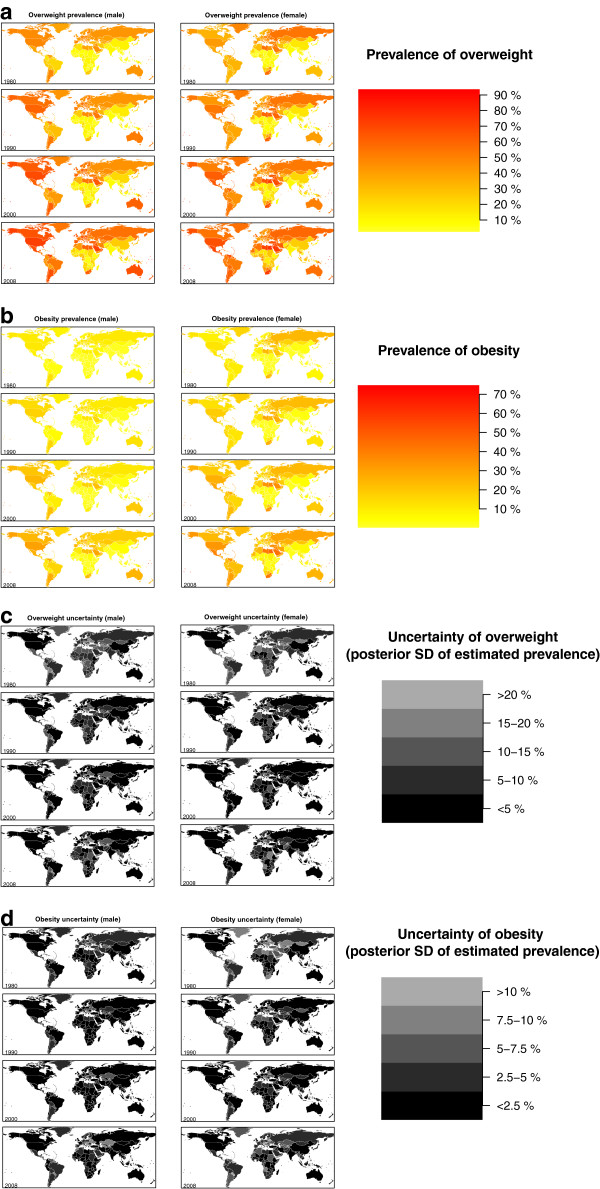
**Age-standardized prevalence of (a) overweight in 1980, 1990, 2000, and 2008, for males and females, (b) obesity in 1980, 1990, 2000, and 2008, for males and females, adults ≥ 20 years.** Panels (**c**) and (**d**) show the posterior standard deviation (akin to standard error) of each estimate.

In 1980, the age-standardized prevalence of overweight among men ranged from 2.8% (1.4-5.1%) in Vietnam to over 50% in 10 countries: Andorra, Cook Islands, Czech Republic, Ireland, Lithuania, Malta, Nauru, Samoa, Slovenia, and Tonga (Figure 2; Additional files 2 and 3). Among women, overweight prevalence was also lowest in Vietnam (2.6%; 1.1-5.0%) and was under 5% in seven other countries in South and Southeast Asia and Central and Eastern Africa. It was over 50% in 29 countries in Eastern, Central, and Western Europe, the Caribbean, Oceania, North Africa, the Middle East, and Southern Africa. By 1990, more than 50% of women were overweight in 47 countries, a number that increased to 74 in 2000 and to 101 in 2008. By 2008, the lowest prevalence of overweight among women was 8.0% (5.1-11.7%) in Bangladesh. Female overweight prevalence was also below 10% in Ethiopia, Madagascar, and Nepal; at the other extreme, it reached over 90% in Cook Islands, Nauru, and Tonga. Male overweight in 2008 ranged from 5.8% (1.7-13.3%) in the Democratic Republic of the Congo to over 90% in the Cook Islands and Nauru (these countries also had prevalences of overweight greater than 90% without age standardization). More than half of men were overweight in 100 countries in 2008. In both 1980 and 2008, the correlation between the male and female prevalence of overweight was 0.85.

In 1980, the age-standardized obesity prevalence in men ranged from 0.3% (0.1-0.5%) in Vietnam to 27.5% (10.3-44.5%) in Nauru (Figure 2; Additional files 2 and 4). Female obesity was as low as 0.3% (0.1-0.7%) in Vietnam, reaching 30.6% (17.2-43.5%) in Kuwait and 32.0% (11.2-52.3%) in Nauru. Male obesity was below 1% in 20 countries and female obesity was below 1% in 11 countries in South and Southeast Asia and Sub-Saharan Africa. By 1990, the prevalence of obesity among women was greater than 25% in 26 countries in Oceania, North Africa and Middle East, the Caribbean, and also in Moldova, the Netherlands, and South Africa; by 2008, this number increased to 80 countries. By 2000, obesity in women already exceeded 50% in four countries in Oceania; these four countries were joined by Kuwait and five more countries in Oceania by 2008. In 2008, female obesity ranged from 1.4% (0.7-2.2%) in Bangladesh and 1.5% (0.9-2.4%) in Madagascar to 70.4% (61.9-78.9%) in Tonga and 74.8% (66.7-82.1%) in Nauru. In 2008, the prevalence of male obesity was below 1% in three countries: Democratic Republic of the Congo, Ethiopia and Bangladesh. More than one half of men were obese in Cook Islands (60.1%, 52.6-67.6%) and Nauru (67.9%, 60.5-75%). Between 1980 and 2008, the correlation between the male and female prevalence of obesity increased slightly from 0.82 to 0.86.

In 1980, one half of the 572 million adults in the world with BMI ≥ 25 kg/m^2^ lived in China (72 million), the United States (70 million), and five other countries (Russia, India, Germany, Italy, and Ukraine). In 2008, the countries with the most overweight people were China (241 million) and the United States (158 million). Half of the world's 1.46 billion overweight people were living in these two countries and seven others: India, Russia, Brazil, Mexico, Germany, Indonesia, and Turkey. The largest absolute increase in the number of overweight people was in China (169 million in 28 years) and the United States (88 million). The largest absolute increase in number of obese people occurred in these two countries (56 million in the United States and 42 million in China), followed by Brazil (20 million) and Mexico (18 million).

**Figure 3:**
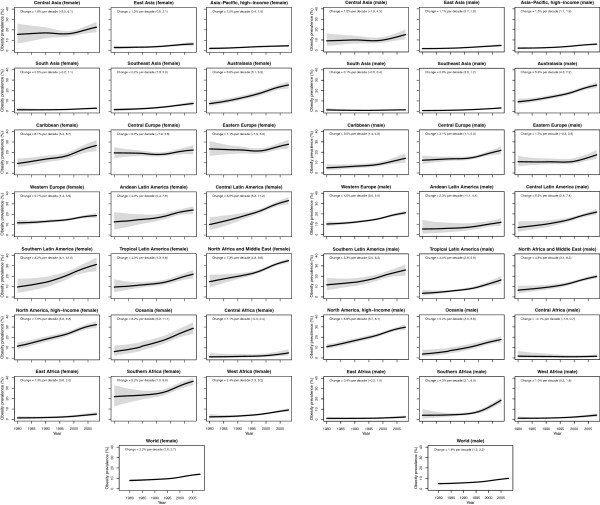
**Trends in age-standardized mean obesity (BMI ≥ 30 kg/m**^**2**^**) by subregion between 1980 and 2008, adults ≥ 20 years.** See Additional file 4 for trends by country. The solid line represents the posterior mean estimate and the shaded area the 95% uncertainty interval.

**Figure 4:**
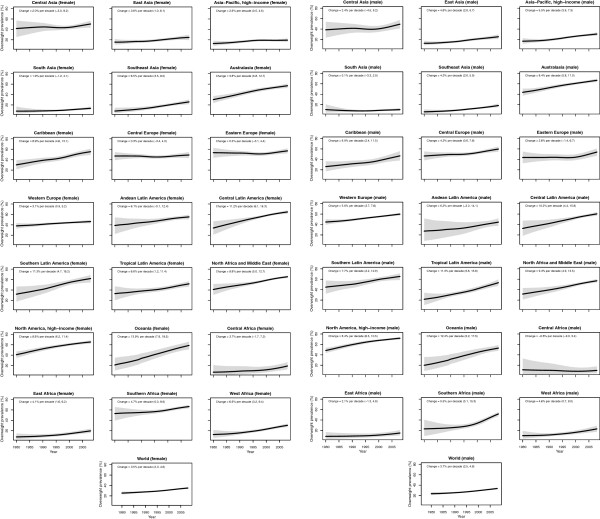
**Trends in age-standardized mean overweight (BMI ≥ 25 kg/m**^**2**^**) by subregion between 1980 and 2008, adults ≥ 20 years.** See Additional file 3 for trends by country. The solid line represents the posterior mean estimate and the shaded area the 95% uncertainty interval.

The regions with the lowest prevalence of obesity in 1980 were South and Southeast Asia, East, Central, and West Sub-Saharan Africa, with prevalences ranging from 1.1% to 1.9% (Figure 3). Central and Eastern Europe had the highest prevalence of obesity (16.3-18.2%) in 1980. Regional patterns of overweight were similar (Figure 4). By 1990, North America was also among the regions with the highest prevalence of obesity (17.6%, 15.9-19.4%), surpassing other regions by 2000 when obesity prevalence reached 24.9% (23.5-26.3%). Due to its large increase in obesity, Southern Latin America also joined regions with high prevalence of obesity by 2000 (22.2%, 19.8-24.6%). In 2008, the regions with the highest obesity prevalence were North Africa and Middle East, Central and Southern Latin America, Southern Sub-Saharan Africa, and high-income North America, with prevalences ranging from 27.4% to 31.1%. By 2008, South and Southeast Asia and Central and East Sub-Saharan Africa still had some of the lowest prevalences of obesity, as did high-income Asia Pacific, with prevalences ranging from 2.2% to 5.4%. On the other hand, Western Sub-Saharan Africa, which had remained a low-obesity region until 2000, experienced a relatively large rise in BMI in the next 8 years, and hence no longer was a region with low prevalence in 2008.

In 16 of 21 regions, the prevalence of female obesity was greater than that of men in every year from 1980 to 2008. In high-income Asia-Pacific, Australasia, Western Europe, Southern Latin America, and Central Sub-Saharan Africa, male obesity exceeded female obesity in some years, usually by a small margin. Male and female obesity prevalence differed most in Southern Sub-Saharan Africa, where 18.7% (15.6-21.5%) of men were obese vs. 36.7% (33.0%-40.2%) of women in 2008.

**Figure 5:**
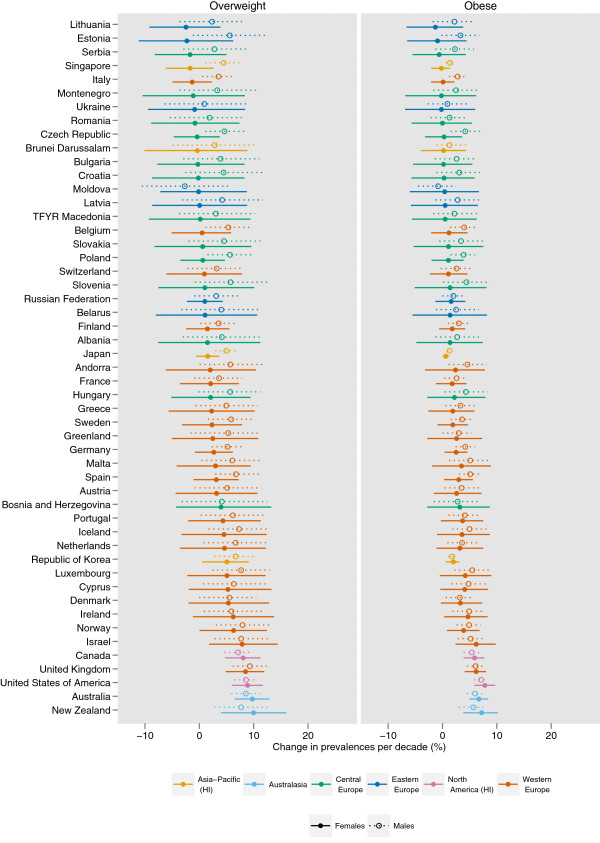
**Change in the age-standardized prevalences of obesity (BMI ≥ 30 kg/m**^**2**^**) and overweight (BMI ≥ 25 kg/m**^**2**^**) in high-income regions and Central and Eastern Europe for male and female adults ≥ 20 years, percentage points per decade.** Note that the absolute change in prevalence is shown, rather than a relative change in the prevalence of obesity / overweight.

**Figure 6:**
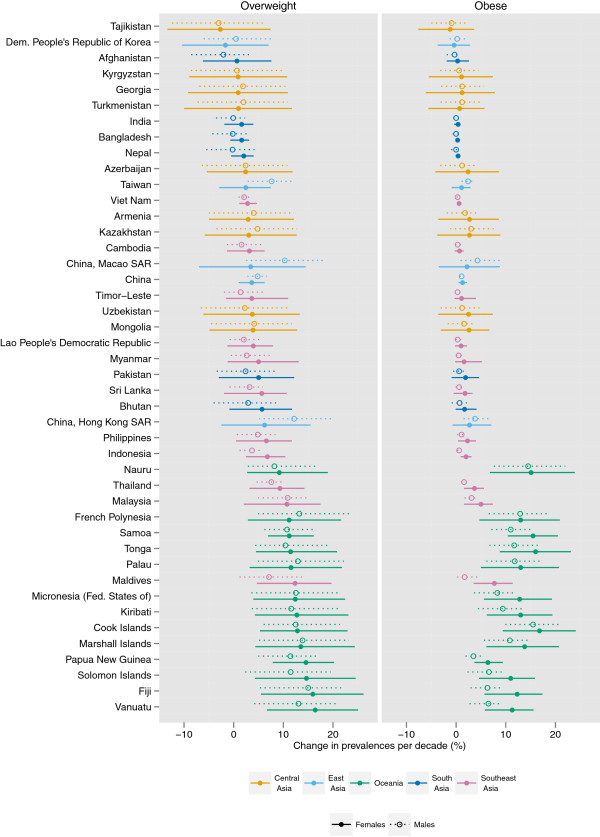
**Change in the age-standardized prevalences of obesity (BMI ≥ 30 kg/m**^**2**^**) and overweight (BMI ≥ 25 kg/m**^**2**^**) in Asia and Oceania for male and female adults ≥ 20 years, percentage points per decade.** Note that the absolute change in prevalence is shown, rather than a relative change in the prevalence of obesity / overweight.

**Figure 7:**
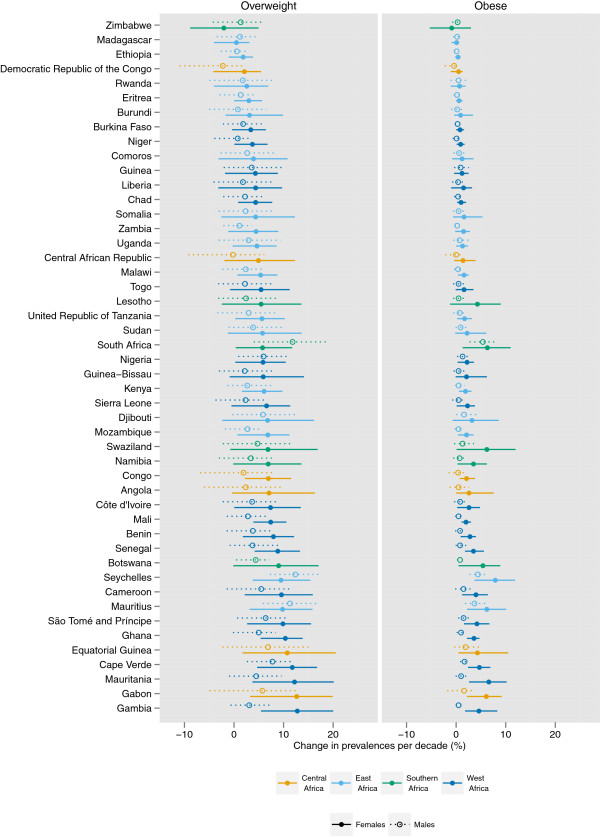
**Change in the age-standardized prevalences of obesity (BMI ≥ 30 kg/m**^**2**^**) and overweight (BMI ≥ 25 kg/m**^**2**^**) in sub-Saharan Africa for male and female adults ≥ 20 years, percentage points per decade.** Note that the absolute change in prevalence is shown, rather than a relative change in the prevalence of obesity / overweight.

**Figure 8:**
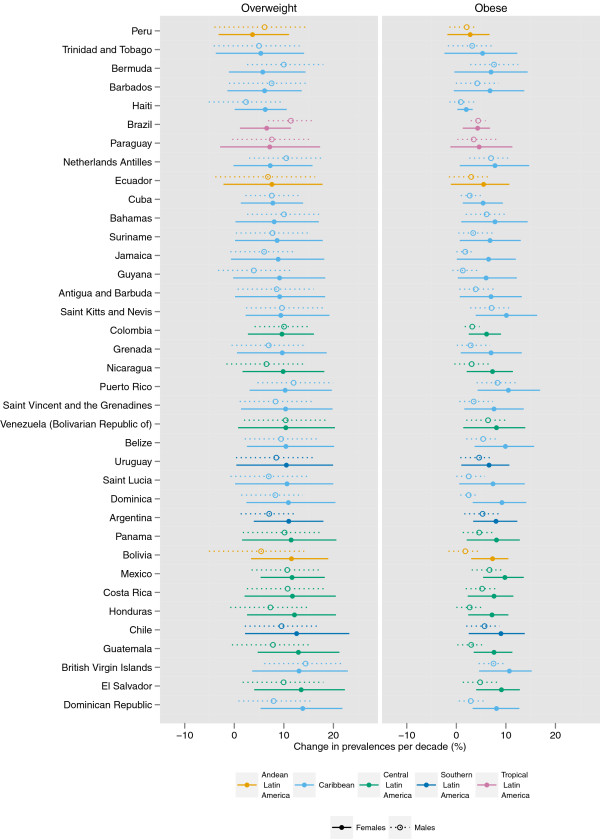
**Change in the age-standardized prevalences of obesity (BMI ≥ 30 kg/m**^**2**^**) and overweight (BMI ≥ 25 kg/m**^**2**^**) in Latin America and Caribbean for male and female adults ≥ 20 years, percentage points per decade.** Note that the absolute change in prevalence is shown, rather than a relative change in the prevalence of obesity / overweight.

**Figure 9:**
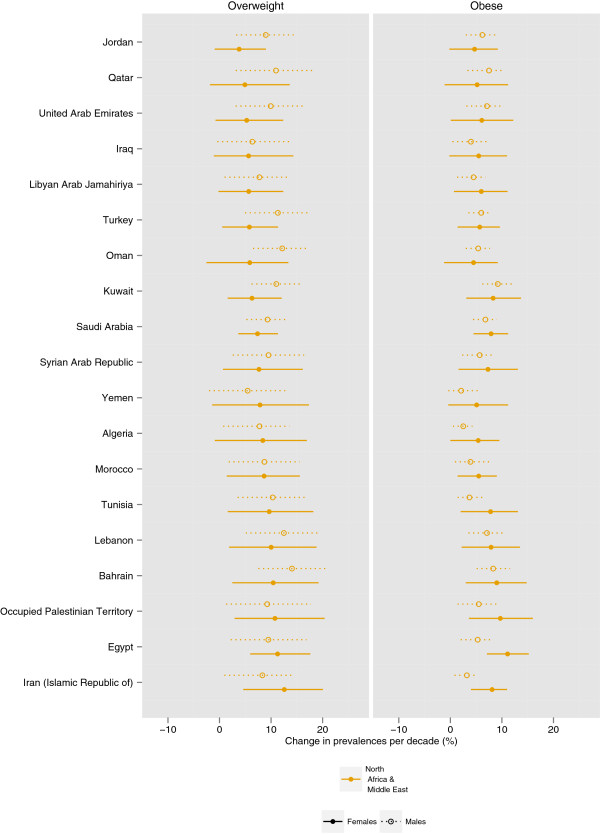
**Change in the age-standardized prevalences of obesity (BMI ≥ 30 kg/m**^**2**^**) and overweight (BMI ≥ 25 kg/m**^**2**^**) in North Africa and Middle East for male and female adults ≥ 20 years, percentage points per decade.** Note that the absolute change in prevalence is shown, rather than a relative change in the prevalence of obesity / overweight.

The prevalence of female obesity increased the most in some countries in Oceania, with the largest increase in Cook Islands (16.8 percentage points per decade, 9.4-24.1, pp > 0.99) and Tonga (16.0 percentage points, 8.8-23.1, pp > 0.99) (Figures 5, 6, 7, 8, 9). The prevalence of obesity increased more than 10 percentage points per decade in 16 countries in Oceania and the Caribbean, and in Egypt. A decline in the prevalence of obesity was estimated for women in 10 countries: Lithuania, Tajikistan, Estonia, Zimbabwe, Serbia, Democratic People's Republic of Korea, Montenegro, Singapore, Ukraine, and Romania; none were statistically distinguishable from no trend (pp ranged from 0.50-0.68). Male obesity increased more than 10 percentage points per decade in seven countries in Oceania, most notably in Nauru (14.5 percentage points per decade, 7.6-22.0, pp > 0.99) and Cook Islands (15.5 percentage points per decade, 9.9-20.7, pp > 0.99). A decline in the prevalence of obesity was estimated for men in seven countries: Tajikistan, Moldova, Democratic Republic of the Congo, Afghanistan, Central African Republic, Nepal, and Bangladesh; none were statistically distinguishable from no trend (pp ranged from 0.41-0.75).

The regions with the largest increase in the prevalence of female obesity were Central Latin America (8.5 percentage points per decade, 5.3-11.2, pp > 0.99), Oceania (8.2 percentage points per decade, 5.2-11.1, pp > 0.99), and Southern Latin America (8.2 percentage points per decade, 4.1-12.0, pp > 0.99); female obesity increased in every region (pp ranging from 0.79 in Central Asia to > 0.99 in 15 regions). The regions with the largest increase in male obesity were North America (6.9 percentage points per decade, 5.7-8.1, pp > 0.99) and Australasia (5.9 percentage points per decade, 4.6-7.2, pp > 0.99), while the regions with the smallest increases were Central Sub-Saharan Africa and South Asia, both of which had trends that were statistically indistinguishable from zero (pp ranged from 0.43-0.66). The absolute annual increase in obesity prevalence was greater during 2000 to 2008 than the 1980s or the 1990s in all 21 regions. In nine regions (Central and South Asia, Central and Eastern Europe, Tropical Latin America, and East, West, Central, and Southern Sub-Saharan Africa), more than half of the rise in obesity over the period 1980 to 2008 occurred in 2000 to 2008.

Diverging trends in obesity were observed among high-income regions, with larger increases in Australasia and North America and smaller increases in the high-income Asia Pacific and Western European regions, especially for females (Figure 3). Female obesity increased 7.6 (5.8-9.3) percentage points per decade in high-income North America and 6.8 (5.1-8.3) percentage points per decade in Australasia, vs. 2.7 (1.4-3.9) and 1.0 (0.4-1.5) percentage points per decade in Western Europe and high-income Asia-Pacific, respectively. Likewise, male obesity increased 6.9 (5.7-8.1) percentage points per decade in high-income North America and 5.9 (4.6-7.2) percentage points per decade in Australasia, vs. 4.0 (3.0-5.0) and 1.5 (1.1-1.9) percentage points per decade in Western Europe and high-income Asia-Pacific, respectively. Based on these trends and on 1980 levels, in 2008 the prevalence of obesity was higher in high-income North America (31.1%, 28.9-33.3%) and Australasia (25.3%, 23.1-27.6%), than in Western Europe (20.0%, 18.5%-21.6%) and high-income Asia-Pacific (5.4%, 4.6-6.2%).

While overweight and obesity prevalence generally increased worldwide, there were differences between men and women in some regions and countries (Figures 5- 9). For example, the prevalence of female obesity increased more than the prevalence of male obesity in 46 of 48 countries in Sub-Saharan Africa and in 35 of 37 countries in Latin America and the Caribbean. In Central Sub-Saharan Africa and Southern Latin America, this resulted in women having a higher prevalence of obesity in 2008 than men, in contrast to their 1980 orders. In contrast, in Western, Central, and Eastern Europe, and in the high-income Asia Pacific countries, the prevalence of obesity increased more among men than women in 41 of 47 countries.

## Discussion

One in every 3 adults in the world was overweight and 1 in every 9 was obese in 2008. Beyond this global average, at least 1 in 5 women were obese in 117 countries and at least 1 in 5 men were obese in 73 countries. Notably, the increase in the prevalence of obesity has accelerated in the last decade compared to the 1980s and 1990s.

The rising obesity and overweight are clearly associated with increases in mean BMI. However, we found that the relationship between mean and prevalence was not linear: BMI increase at low BMI levels results in a smaller increase in obesity than an identical absolute increase at higher BMIs (Figure 1). The relationship between obesity and mean BMI also changed over time: in 2008, the prevalence of obesity was greater at all mean BMIs than it had been in earlier decades, indicating that the population BMI distributions is likely to have become wider over time. If recent trends in mean BMI continue [6], the prevalence of obesity will continue to rise at the recent alarmingly high rates.

This study is the first report of adult overweight and obesity prevalence by country, year, and sex. The strengths and innovations of this study include analysis of long-term trends; large amount of high-quality measured population-based data accessed and used; the use of a Bayesian hierarchical model for estimating mean BMI, incorporating non-linear time trends; systematic conversion of mean BMI to overweight and obesity prevalence, including adjusting for age, sex, region, and time; and systematic analysis of uncertainty. The main limitation of our study is that data gaps remained despite our extensive data seeking, especially in the 1980s and for men through the 1990s. We did not model the full population distribution of BMI by country, which should be a topic for future research. Our analysis did not consider trends in central adiposity due to a lack of population-based data, nor did it quantify within-country disparities by socioeconomic status or race [9-11]. Finally, we did not estimate childhood and adolescent adiposity. Child and adolescent overweight and obesity are important because childhood weight gain may have larger adverse effects than weight gain during adulthood due to the longer exposure, and because they are predictive of future trends in overweight and obesity in adults. Measuring overweight and obesity in school-aged children and adolescents is challenging for several reasons: first, there is an age gap in survey data as most surveys on maternal and child health include data up to 5 years of age and those related to noncommunicable disease risk factors and behaviors target adults; second, until the 2007 introduction of WHO growth standards, there was little consensus on definitions of overweight and obesity in children aged 5 years to 19 years. This has resulted in incomparable measures, available in the sparse published literature.

The United Nations General Assembly High-Level Meeting on the Prevention and Control of Noncommunicable Diseases in September 2011 requested that WHO develop targets for key noncommunicable disease indicators, which may include the prevalence of obesity. Data on historical trends allows setting ambitious, yet feasible targets [12]. Our estimates provide a consistent baseline for such empirical targets. The tenth best performing percentile of countries, ranked from lowest to highest absolute increase in obesity, experienced an increase of 0.6 percentage points per decade from 1980 to 2008. The relative change at the tenth percentile was around a 9% increase over 15 years for women and 25% for men. We estimated nonsignificant change (at the pp=0.75 level) in obesity prevalence in 15 countries, while the remainder experienced a statistically significant increase in obesity. Most of these countries maintained low levels of obesity prevalence through food scarcity rather than effective policy action, and thus their experience may not be the appropriate baseline for policies that aim to halt the rise in obesity. Therefore, given the widespread rise in obesity, a key question is how ambitious an obesity target should be relative to the historical experience.

Disentangling the contributions of declines in physical activity, changes in calorie consumption and composition, and other factors to the recent increases in mean BMI and overweight and obesity prevalence are currently active areas of research [13,14]. The data presented in this paper can be used to generate and evaluate hypotheses about these trends via ecological analyses that explore the relationship between trends in overweight/obesity prevalence and in their possible causes. However, we must also carry out rigorous intervention studies to test programs and actions that may curb and reverse these trends [15].

## Conclusions

Our systematic analysis of population-based data sources helped unpack the “global obesity pandemic” into its constituent trends by country, region, and sex. We found that, although the magnitude of the rise in obesity varies by region, country, and gender, stability in obesity prevalence was rare. Moreover, the rise in obesity has accelerated in the last decade. This documentation of country trends in obesity prevalence may be used to set targets as requested by the United Nations General Assembly's High-Level Meeting on the Prevention and Control of Noncommunicable Diseases.

## Abbreviations

BMI: Body mass index; WHO: World Health Organization.

## Competing interests

The authors declare that they have no competing interests.

## Authors’ contributions

GAS, GD, and ME developed the study concept. GAS, GMS, YL, and JKL analyzed databases and prepared results, with input from GD, ME, MMF, and CJP. GAS and ME wrote the first draft of the paper. Other Writing and Global Analysis Group members contributed to study design, analysis, and writing of manuscript. ME and GAS oversaw the research. ME is the study guarantor. GAS, MJC, and LMR are staff members of WHO. The authors alone are responsible for the views expressed in this publication and they do not necessarily represent the decisions, policy, or views of WHO. All authors read and approved the final manuscript.

## Global Burden of Metabolic Risk Factors of Chronic Diseases Collaborating Group (Body Mass Index)

**Writing and Global Analysis Group:** Gretchen A Stevens, Gitanjali M Singh, Yuan Lu, Goodarz Danaei, John Lin, Mariel M Finucane, Adil N Bahalim, Russell K McIntire, Hialy R Gutierrez, Melanie Cowan, Christopher J Paciorek, Leanne Riley, Majid Ezzati.

**Country data group:** Geir Aamodt; Ziad Abdeen; Nabila A Abdella; Hanan F Abdul-Rahim; Juliet Addo; Mohamed M Ali; Mohannad Al-Nsour; Ramachandran Ambady; Pertti Aro; Carlo M Barbagallo; Alberto Barceló; Henrique Barros; Leonelo E Bautista; Peter Bjerregaard; Enzo Bonora; Pascal Bovet; Grazyna Broda; Ian J Brown; Michael Bursztyn; Antonio Cabrera de León; Francesco P Cappuccio; Katia Castetbon; Somnath Chatterji; Zhengming Chen; Chien-Jen Chen; Lily Chua; Renata Cífková; Linda J Cobiac; Anna Maria Corsi; Cora L Craig; Saeed Dastgiri; Martha S de Sereday; Gonul Dinc; Yasufumi Doi; Eleonora Dorsi; Nico Dragano; Adam Drewnowski; Paul Elliott; Anders Engeland; Alireza Esteghamati; Jian-Gao Fan; Catterina Ferreccio; Nélida S Fornés; Flávio D Fuchs; Simona Giampaoli; Sidsel Graff-Iversen; Janet F Grant; Ramiro Guerrero Carvajal; Martin C Gulliford; Rajeev Gupta; Prakash C Gupta; Oye Gureje; Noor Heim; Joachim Heinrich; Tomas Hemmingsson; Victor M Herrera; Suzanne C Ho; Michelle Holdsworth; Wilma M Hopman; Abdullatif Husseini; Nayu Ikeda; Bjarne K Jacobsen; Tazeen H Jafar; Mohsen Janghorbani; Grazyna Jasienska; Michel R Joffres; Jost B Jonas; Ofra Kalter-Leibovici; Ioannis Karalis; Joanne Katz; Lital Keinan-Boker; Paul Kelly; Omid Khalilzadeh; Young-Ho Khang; Stefan Kiechl; Maressa P Krause; Yadlapalli S Kusuma; Arnulf Langhammer; Jeannette Lee; Claire Lévy-Marchal; Yanping Li; Yuqiu Li; Stephen Lim; Cheng-Chieh Lin; Lauren Lissner; Patricio Lopez-Jaramillo; Roberto Lorbeer; Guansheng Ma; Stefan Ma; Francesc Macià; Dianna J Magliano; Marcia Makdisse; Roberto Miccoli; Juhani Miettola; Jaime Miranda; Mostafa K Mohamed; V Mohan; Salim Mohanna; Ali Mokdad; Dante D Morales; Lorenza M Muiesan; Iraj Nabipour; Vinay Nangia; Barbara Nemesure; Martin Neovius; Kjersti A Nerhus; Flavio Nervi; Hannelore Neuhauser; Minh Nguyen; Ayse E Önal; Altan Onat; Myriam Orostegui; Hermann Ouedraogo; Demosthenes B Panagiotakos; Francesco Panza; Yongsoo Park; Mangesh S Pednekar; Marco A Peres; Rafael Pichardo; Hwee Pin Phua; Francesco Pistelli; Pedro Plans; Dorairaj Prabhakaran; Roaeid B Ragab; Olli T Raitkari; Sanjay Rampal; Finn Rasmussen; Josep Redon; Luis Revilla; Victoria Reyes-García; Fernando Rodriguez-Artalejo; Luis Rosero-Bixby; Harshpal S Sachdev; José R Sánchez; Selim Y Sanisoglu; Norberto Schapochnik; Lluís Serra-Majem; Rahman Shiri; Xiao Ou Shu; Leon A Simons; Margaret Smith; Vincenzo Solfrizzi; Emily Sonestedt; Pär Stattin; Aryeh D Stein; George S Stergiou; Jochanan Stessman; Akihiro Sudo; Valter Sundh; Kristina Sundquist; Johan Sundström; Martin Tobias; Liv E Torheim; Josep A Tur; Ana I Uhernik; Flora A Ukoli; Mark P Vanderpump; Jose Javier Varo; Marit B Veierød; Gustavo Velásquez-Meléndez; Monique Verschuren; Salvador Villalpando; Jesus Vioque; Peter Vollenweider; Mark Ward; Sarwono Waspadji; Johann Willeit; Mark Woodward; Liang Xu; Fei Xu; Gonghuan Yang; Li-Chia Yeh; Jin-Sang Yoon; Qisheng You; Wei Zheng.

## Supplementary Material

Additional file 1 Prevalence of male and female overweight and obesity, adults ≥ 20 years, by country and year, 1980–2008. Click here for file

Additional file 2 **Prevalences of (a) male obesity (BMI ≥ 30 kg/m**^**2**^**), (b) male overweight (BMI ≥ 25 kg/m**^**2**^**), (c) female obesity (BMI ≥ 30 kg/m**^**2**^**), (d) female overweight (BMI ≥ 25 kg/m**^**2**^**), adults ≥ 20 years in 1980 and 2008.** Click here for file

Additional file 3 **Trends in age-standardized mean overweight (BMI ≥ 25 kg/m**^2^**) by country between 1980 and 2008, adults ≥ 20 years.** The solid line represents the posterior mean estimate and the shaded area the 95% uncertainty interval. Click here for file

Additional file 4 **Trends in age-standardized mean obesity (BMI ≥ 25 kg/m**^**2**^**) by country between 1980 and 2008, adults ≥ 20 years.** The solid line represents the posterior mean estimate and the shaded area the 95% uncertainty interval. Click here for file
