# Supplementary material for: National, regional, and global trends in adult overweight and obesity prevalences
Source: Popul Health Metr. 2012 Nov 20;10:22. doi: 10.1186/1478-7954-10-22 (PMC3543235; doi:10.1186/1478-7954-10-22)
Supplement: Additional file 1 — Prevalence of male and female overweight and obesity, adults ≥ 20 years, by country and year, 1980–2008. [file 1478-7954-10-22-S1.pdf]

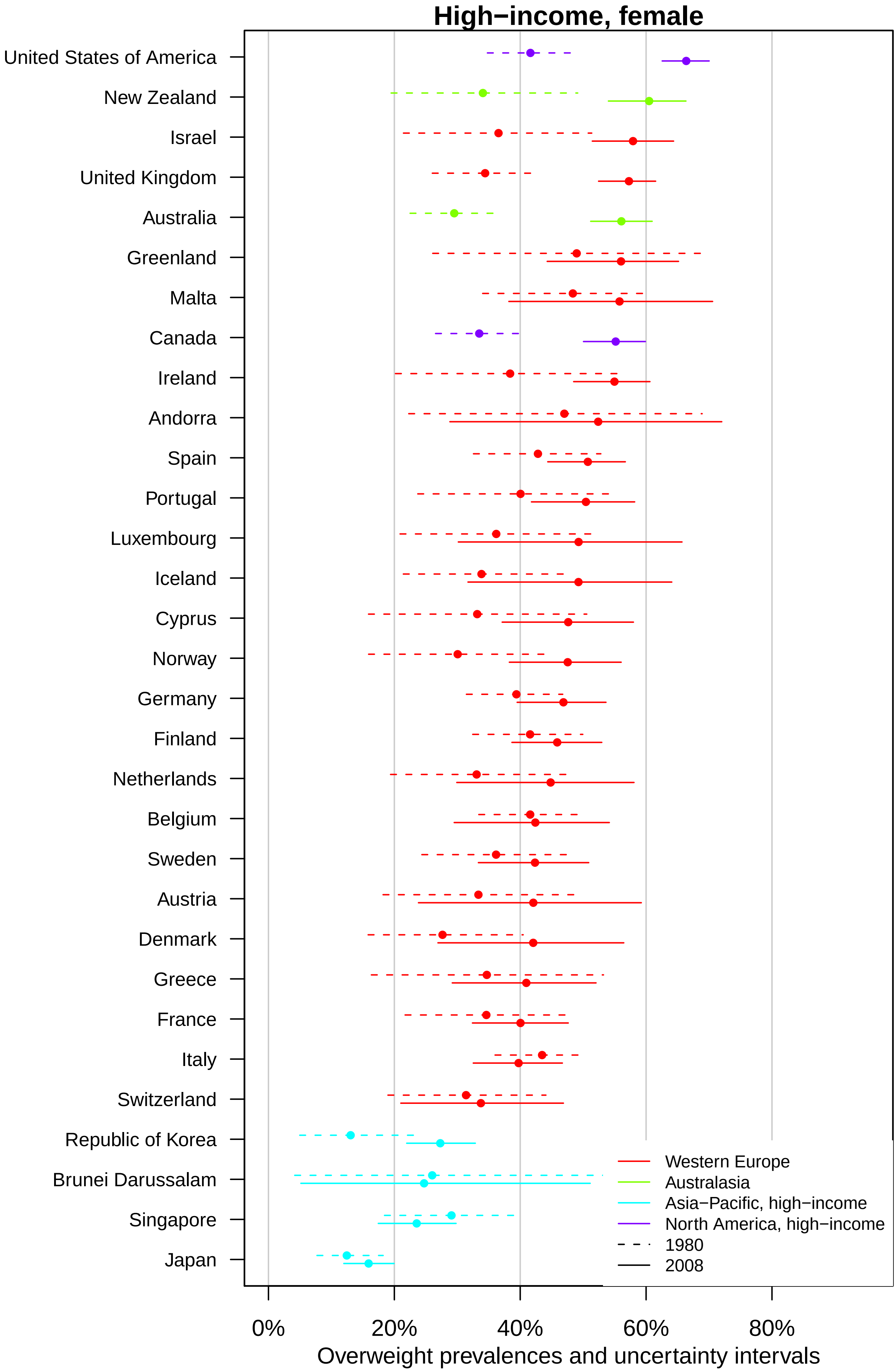

High−income, female
United States of America
New Zealand
Israel
United Kingdom
Australia
Greenland
Malta
Canada
Ireland
Andorra
Spain
Portugal
Luxembourg
Iceland
Cyprus
Norway
Germany
Finland
Netherlands
Belgium
Sweden
Austria
Denmark
Greece
France
Italy
Switzerland
Republic of Korea
Brunei Darussalam
Singapore
Japan
Western Europe
Australasia
Asia−Pacific, high−income
North America, high−income
1980
2008
0%
20%
40%
60%
80%
Overweight prevalences and uncertainty intervals

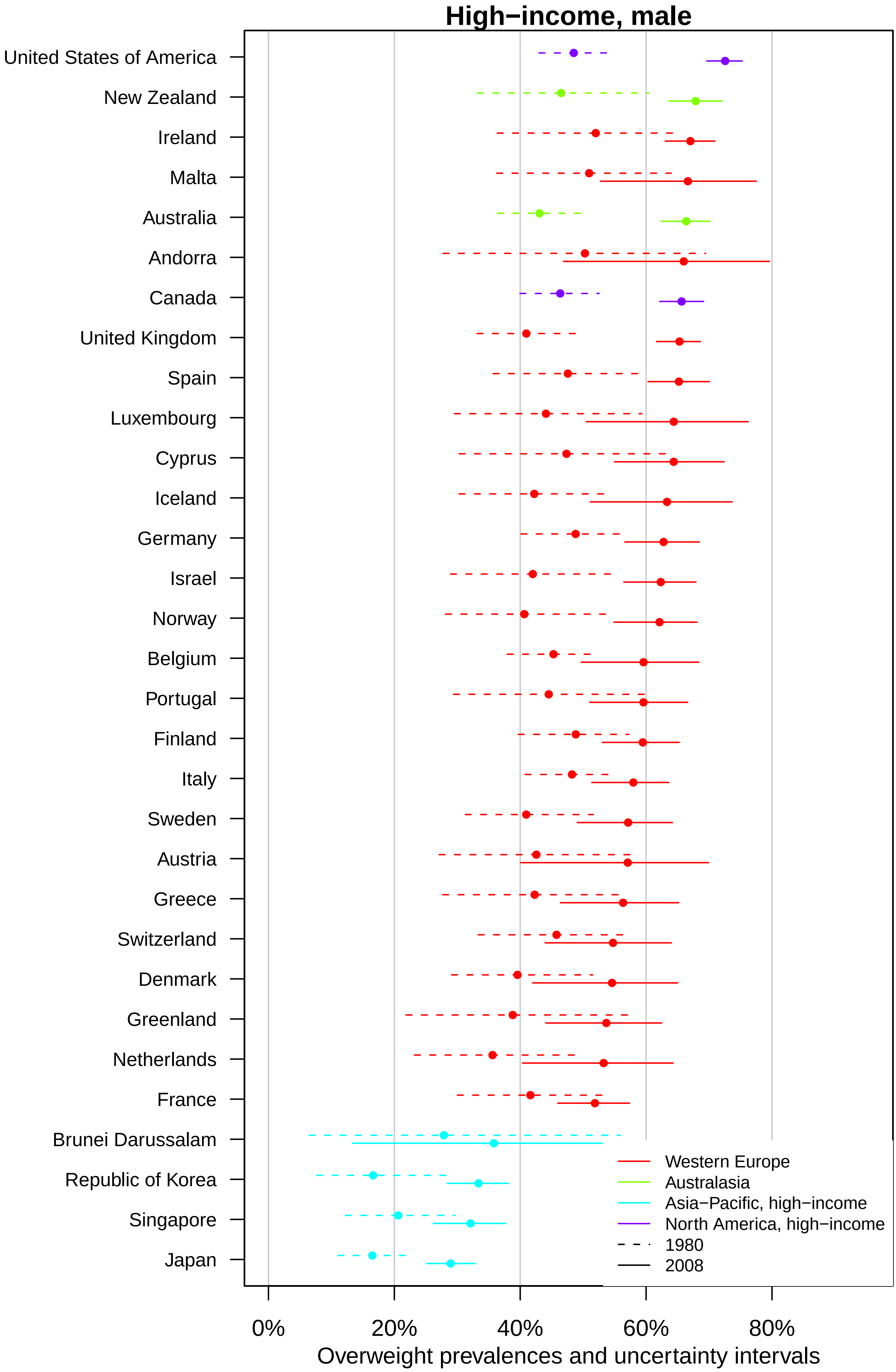

High−income, male
United States of America
New Zealand
Ireland
Malta
Australia
Andorra
Canada
United Kingdom
Spain
Luxembourg
Cyprus
Iceland
Germany
Israel
Norway
Belgium
Portugal
Finland
Italy
Sweden
Austria
Greece
Switzerland
Denmark
Greenland
Netherlands
France
Brunei Darussalam
Republic of Korea
Singapore
Japan
Western Europe
Australasia
Asia−Pacific, high−income
North America, high−income
1980
2008
0%
20%
40%
60%
80%
Overweight prevalences and uncertainty intervals

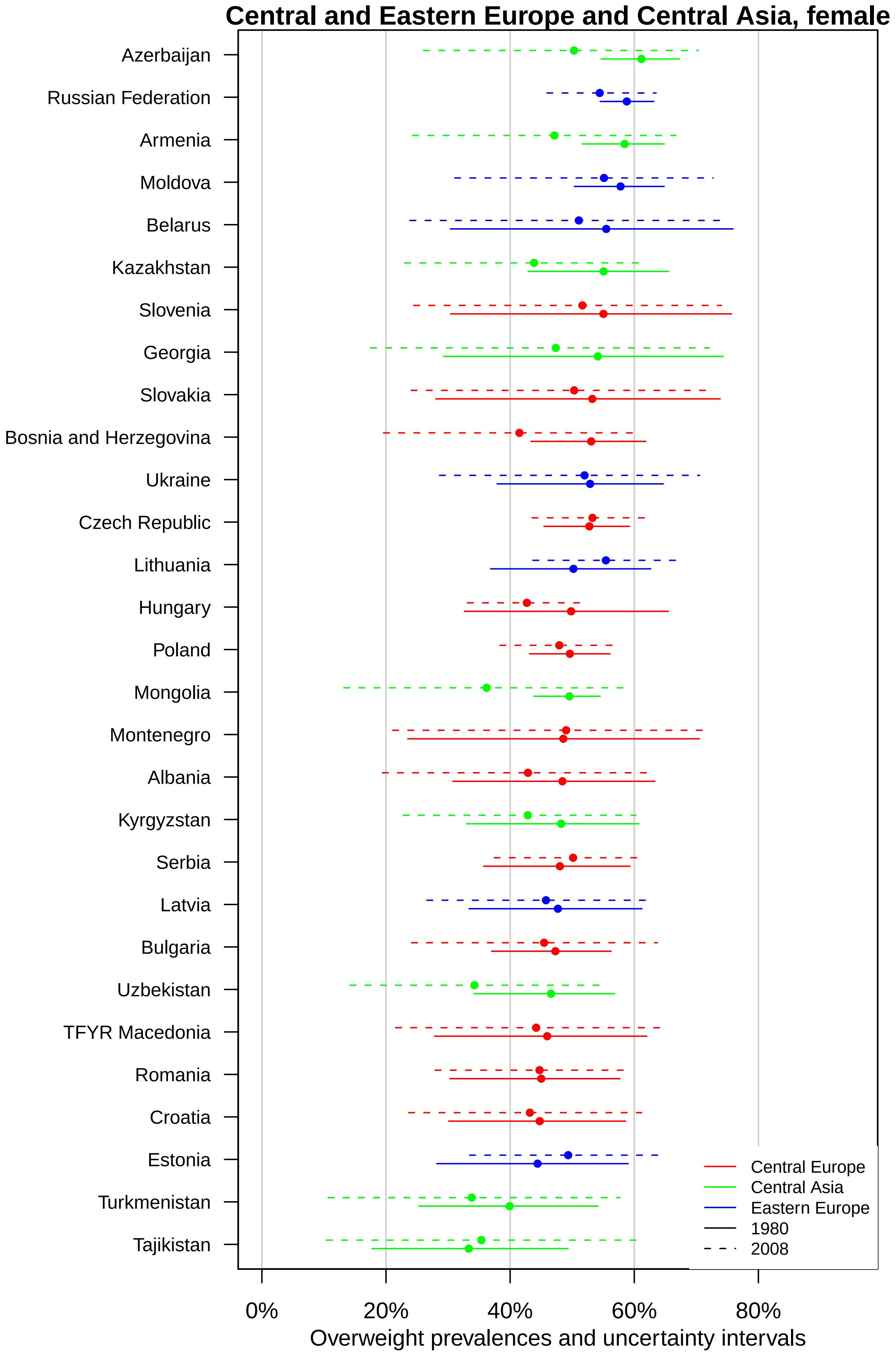

Central and Eastern Europe and Central Asia, female
Azerbaijan
Russian Federation
Armenia
Moldova
Belarus
Kazakhstan
Slovenia
Georgia
Slovakia
Bosnia and Herzegovina
Ukraine
Czech Republic
Lithuania
Hungary
Poland
Mongolia
Montenegro
Albania
Kyrgyzstan
Serbia
Latvia
Bulgaria
Uzbekistan
TFYR Macedonia
Romania
Croatia
Estonia
Turkmenistan
Tajikistan
Central Europe
Central Asia
Eastern Europe
1980
2008
0%
20%
40%
60%
80%
Overweight prevalences and uncertainty intervals

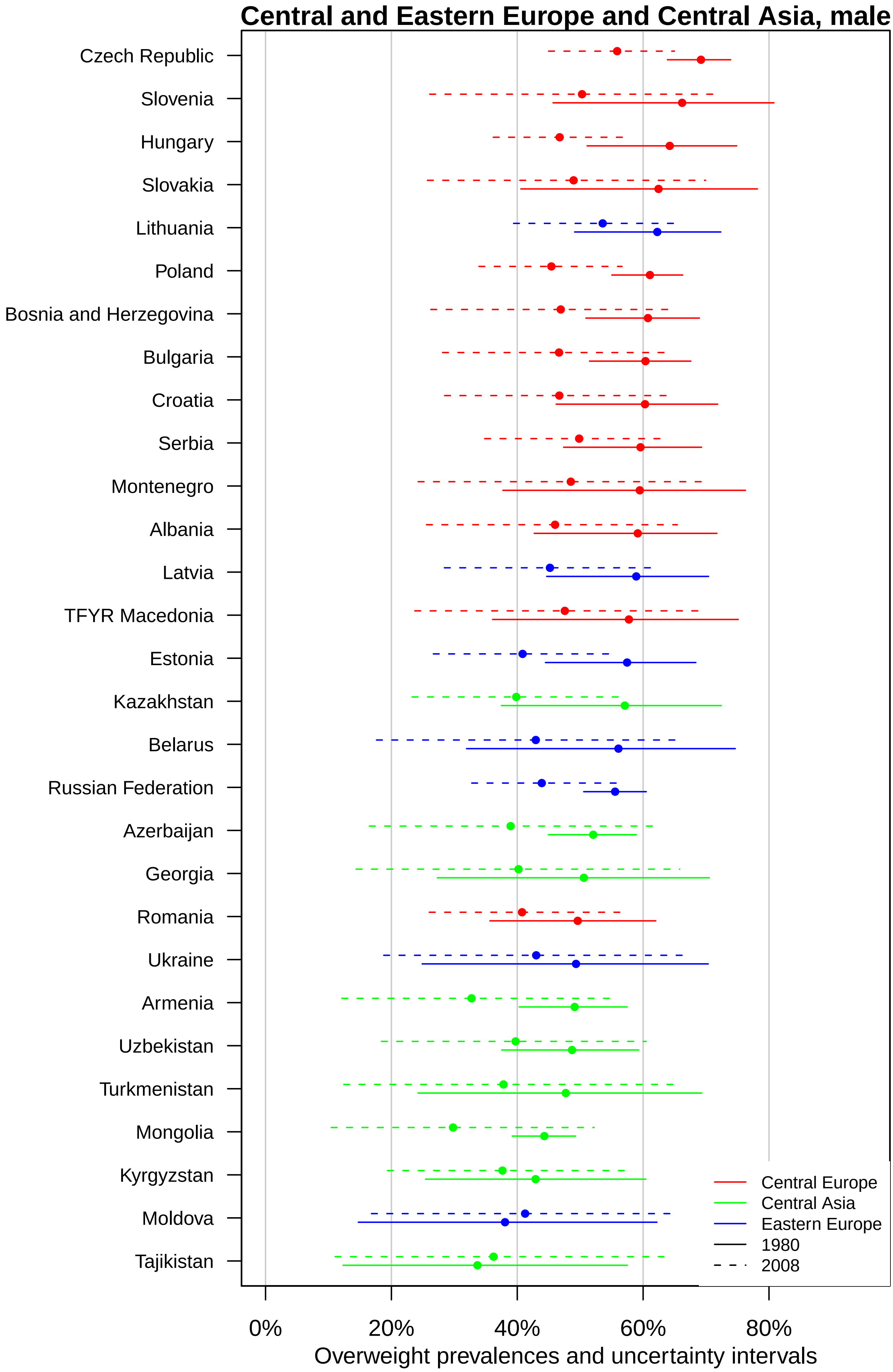
Central and Eastern Europe and Central Asia, male
Czech Republic
Slovenia
Hungary
Slovakia
Lithuania
Poland
Bosnia and Herzegovina
Bulgaria
Croatia
Serbia
Montenegro
Albania
Latvia
TFYR Macedonia
Estonia
Kazakhstan
Belarus
Russian Federation
Azerbaijan
Georgia
Romania
Ukraine
Armenia
Uzbekistan
Turkmenistan
Mongolia
Kyrgyzstan
Moldova
Tajikistan
Central Europe
Central Asia
Eastern Europe
1980
2008
0%
20%
40%
60%
80%
Overweight prevalences and uncertainty intervals

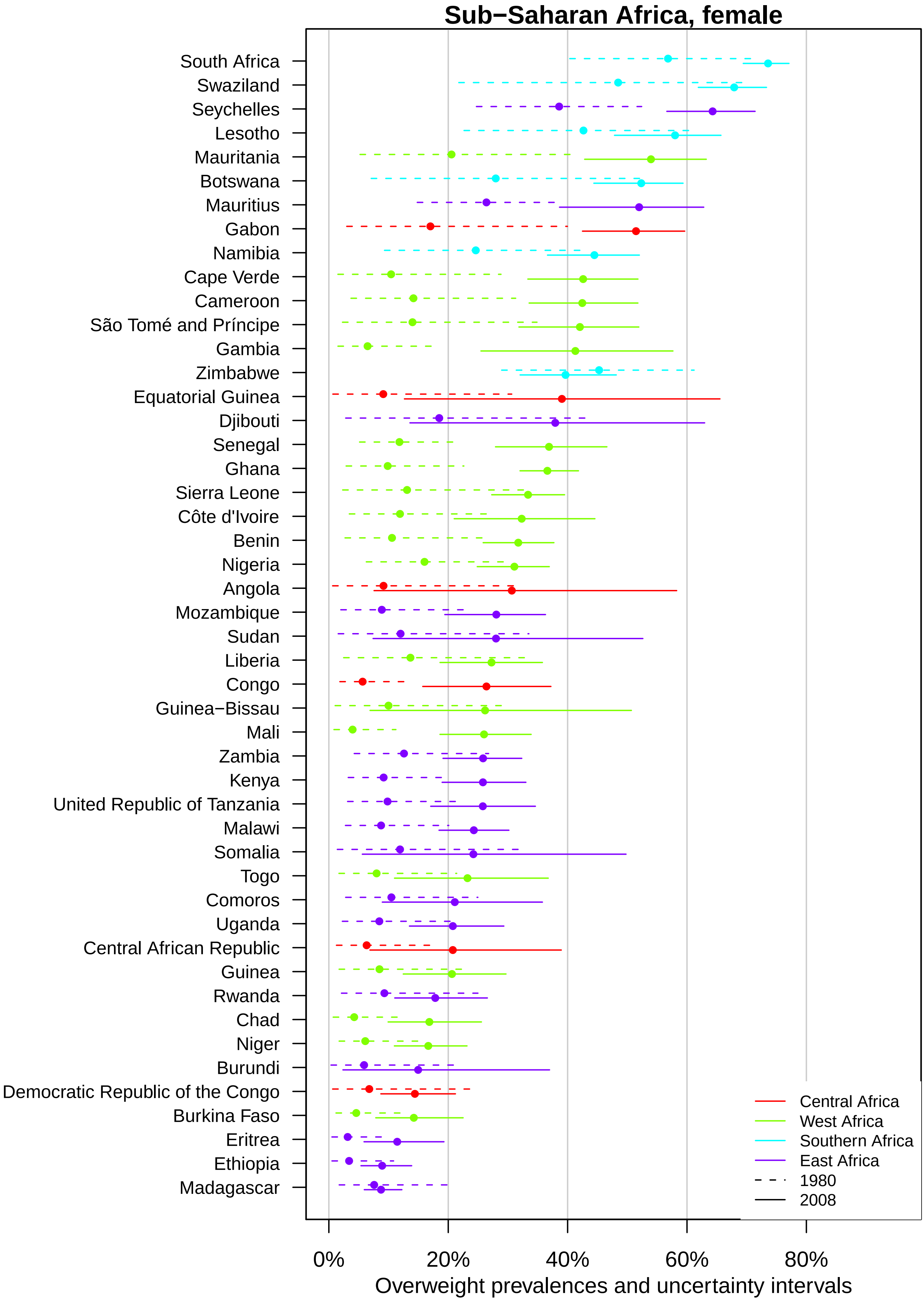

Sub−Saharan Africa, female
South Africa
Swaziland
Seychelles
Lesotho
Mauritania
Botswana
Mauritius
Gabon
Namibia
Cape Verde
Cameroon
São Tomé and Príncipe
Gambia
Zimbabwe
Equatorial Guinea
Djibouti
Senegal
Ghana
Sierra Leone
Côte d'Ivoire
Benin
Nigeria
Angola
Mozambique
Sudan
Liberia
Congo
Guinea−Bissau
Mali
Zambia
Kenya
United Republic of Tanzania
Malawi
Somalia
Togo
Comoros
Uganda
Central African Republic
Guinea
Rwanda
Chad
Niger
Burundi
Democratic Republic of the Congo
Burkina Faso
Eritrea
Ethiopia
Madagascar
Central Africa
West Africa
Southern Africa
East Africa
1980
2008
0%
20%
40%
60%
80%
Overweight prevalences and uncertainty intervals

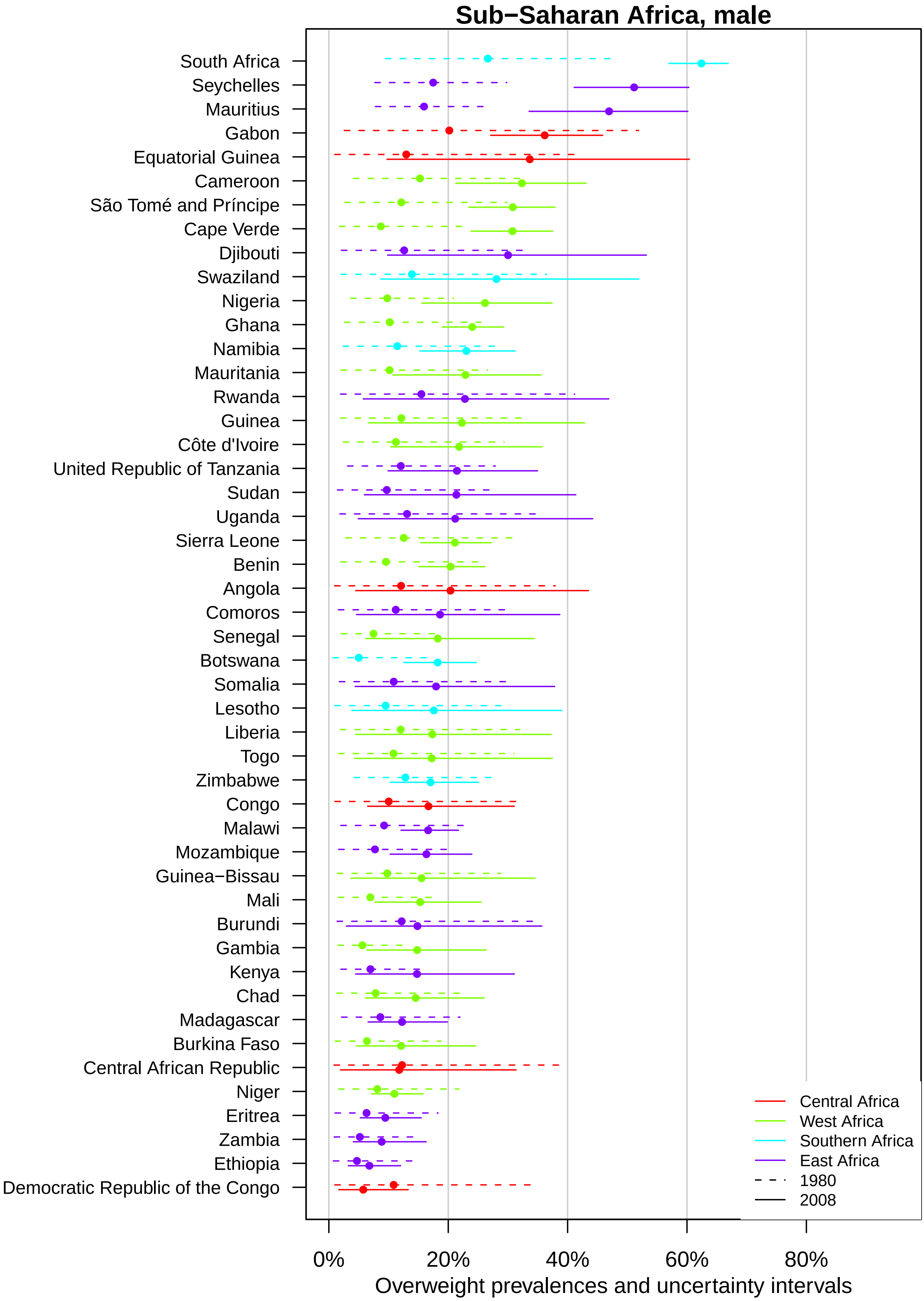
Sub−Saharan Africa, male
South Africa
Seychelles
Mauritius
Gabon
Equatorial Guinea
Cameroon
São Tomé and Príncipe
Cape Verde
Djibouti
Swaziland
Nigeria
Ghana
Namibia
Mauritania
Rwanda
Guinea
Côte d'Ivoire
United Republic of Tanzania
Sudan
Uganda
Sierra Leone
Benin
Angola
Comoros
Senegal
Botswana
Somalia
Lesotho
Liberia
Togo
Zimbabwe
Congo
Malawi
Mozambique
Guinea−Bissau
Mali
Burundi
Gambia
Kenya
Chad
Madagascar
Burkina Faso
Central African Republic
Niger
Eritrea
Zambia
Ethiopia
Democratic Republic of the Congo
Central Africa
West Africa
Southern Africa
East Africa
1980
2008
0%
20%
40%
60%
80%
Overweight prevalences and uncertainty intervals

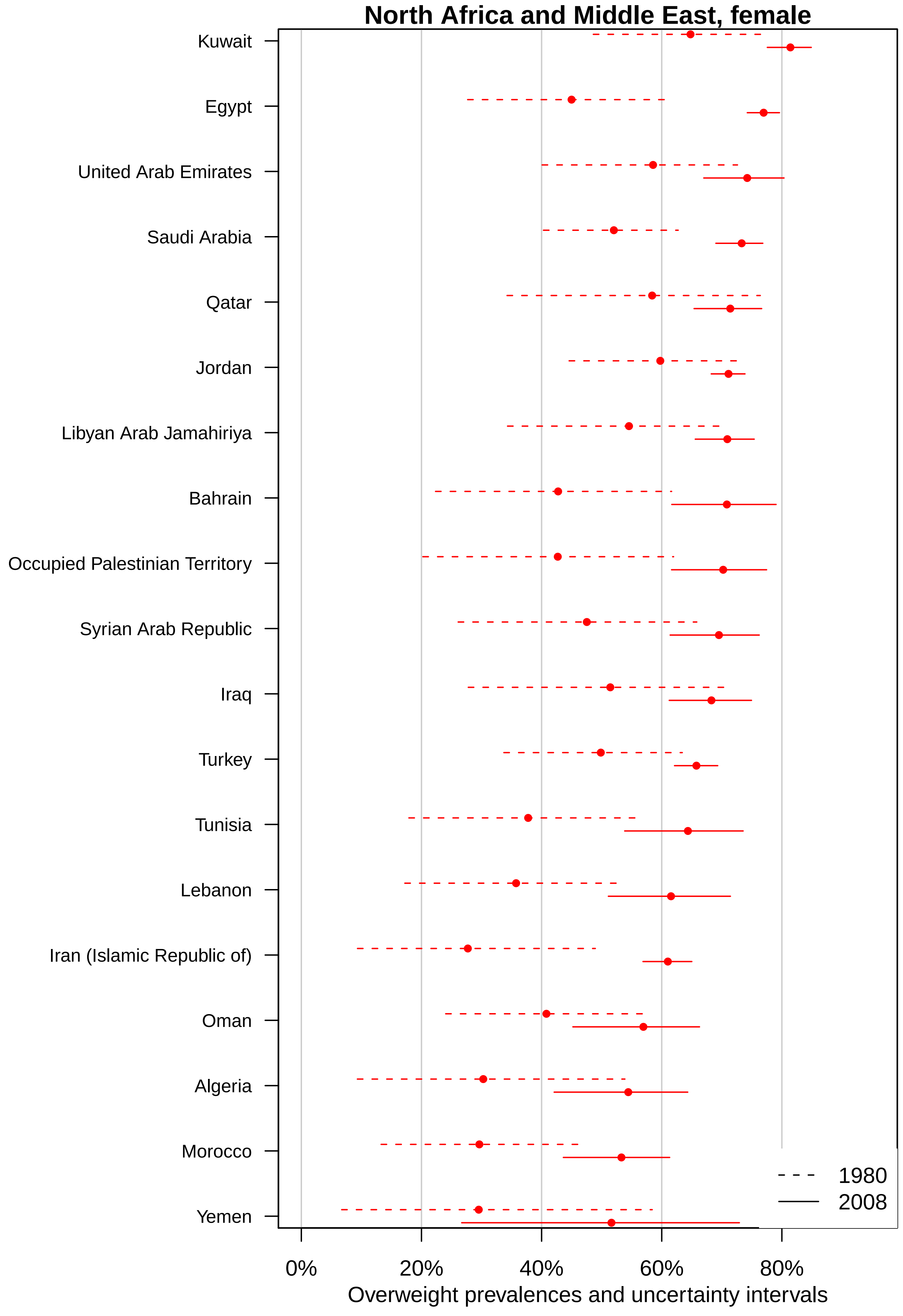
North Africa and Middle East, female
Kuwait
Egypt
United Arab Emirates
Saudi Arabia
Qatar
Jordan
Libyan Arab Jamahiriya
Bahrain
Occupied Palestinian Territory
Syrian Arab Republic
Iraq
Turkey
Tunisia
Lebanon
Iran (Islamic Republic of)
Oman
Algeria
Morocco
Yemen
1980
2008
0%
20%
40%
60%
80%
Overweight prevalences and uncertainty intervals

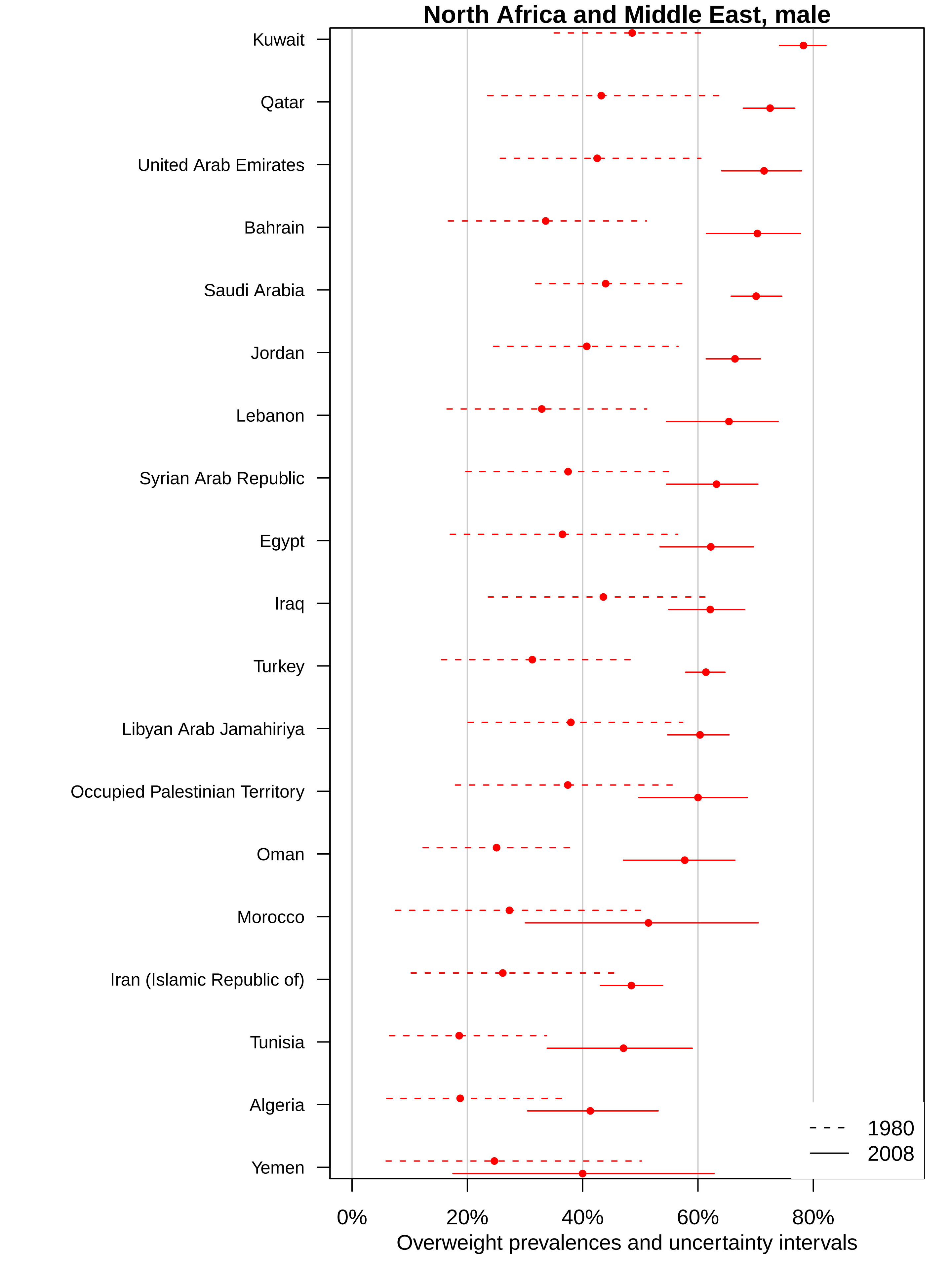
North Africa and Middle East, male
Kuwait
Qatar
United Arab Emirates
Bahrain
Saudi Arabia
Jordan
Lebanon
Syrian Arab Republic
Egypt
Iraq
Turkey
Libyan Arab Jamahiriya
Occupied Palestinian Territory
Oman
Morocco
Iran (Islamic Republic of)
Tunisia
Algeria
Yemen
1980
2008
0%
20%
40%
60%
80%
Overweight prevalences and uncertainty intervals

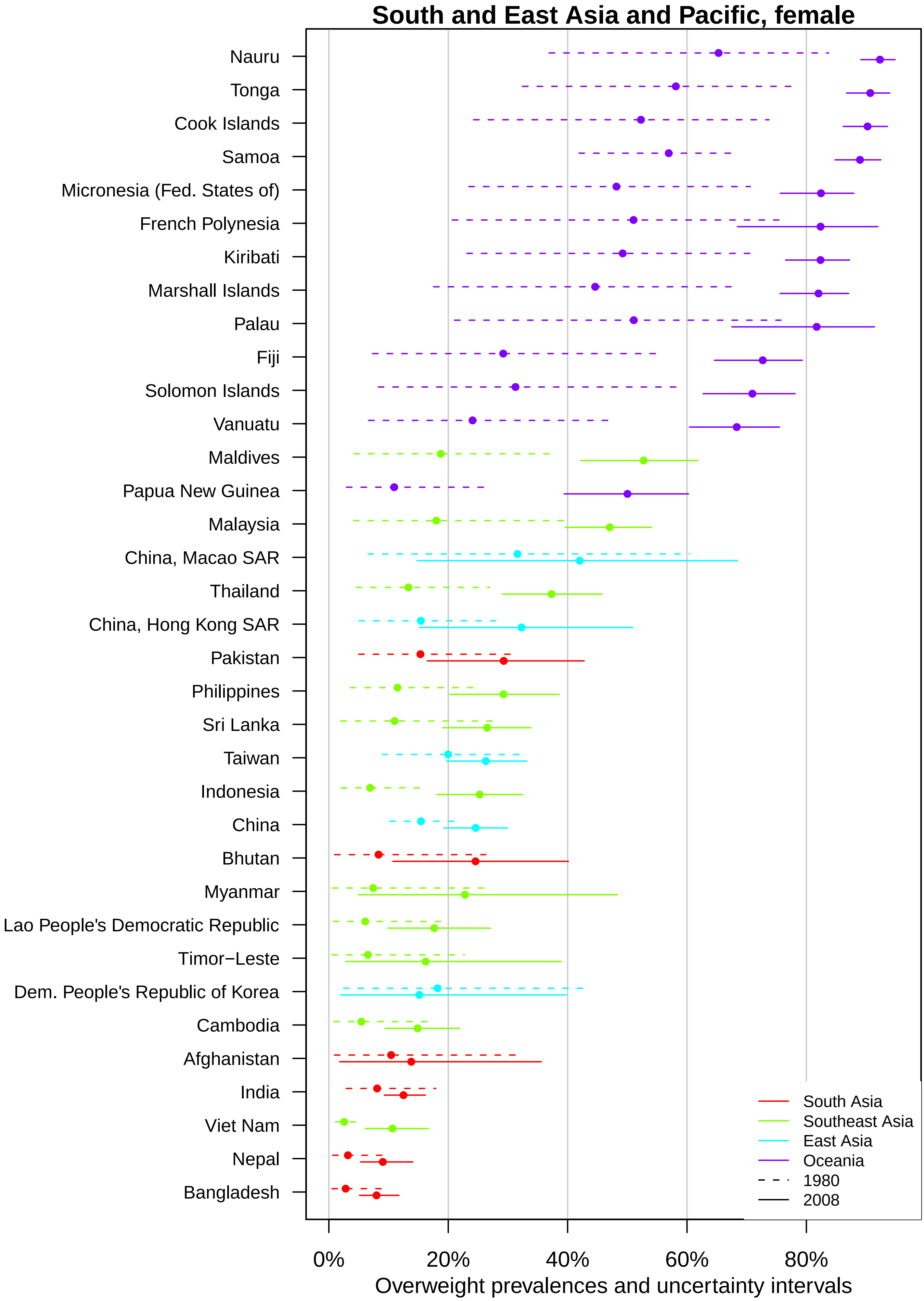
South and East Asia and Pacific, female
Nauru
Tonga
Cook Islands
Samoa
Micronesia (Fed. States of)
French Polynesia
Kiribati
Marshall Islands
Palau
Fiji
Solomon Islands
Vanuatu
Maldives
Papua New Guinea
Malaysia
China, Macao SAR
Thailand
China, Hong Kong SAR
Pakistan
Philippines
Sri Lanka
Taiwan
Indonesia
China
Bhutan
Myanmar
Lao People's Democratic Republic
Timor−Leste
Dem. People's Republic of Korea
Cambodia
Afghanistan
India
Viet Nam
Nepal
Bangladesh
South Asia
Southeast Asia
East Asia
Oceania
1980
2008
0%
20%
40%
60%
80%
Overweight prevalences and uncertainty intervals

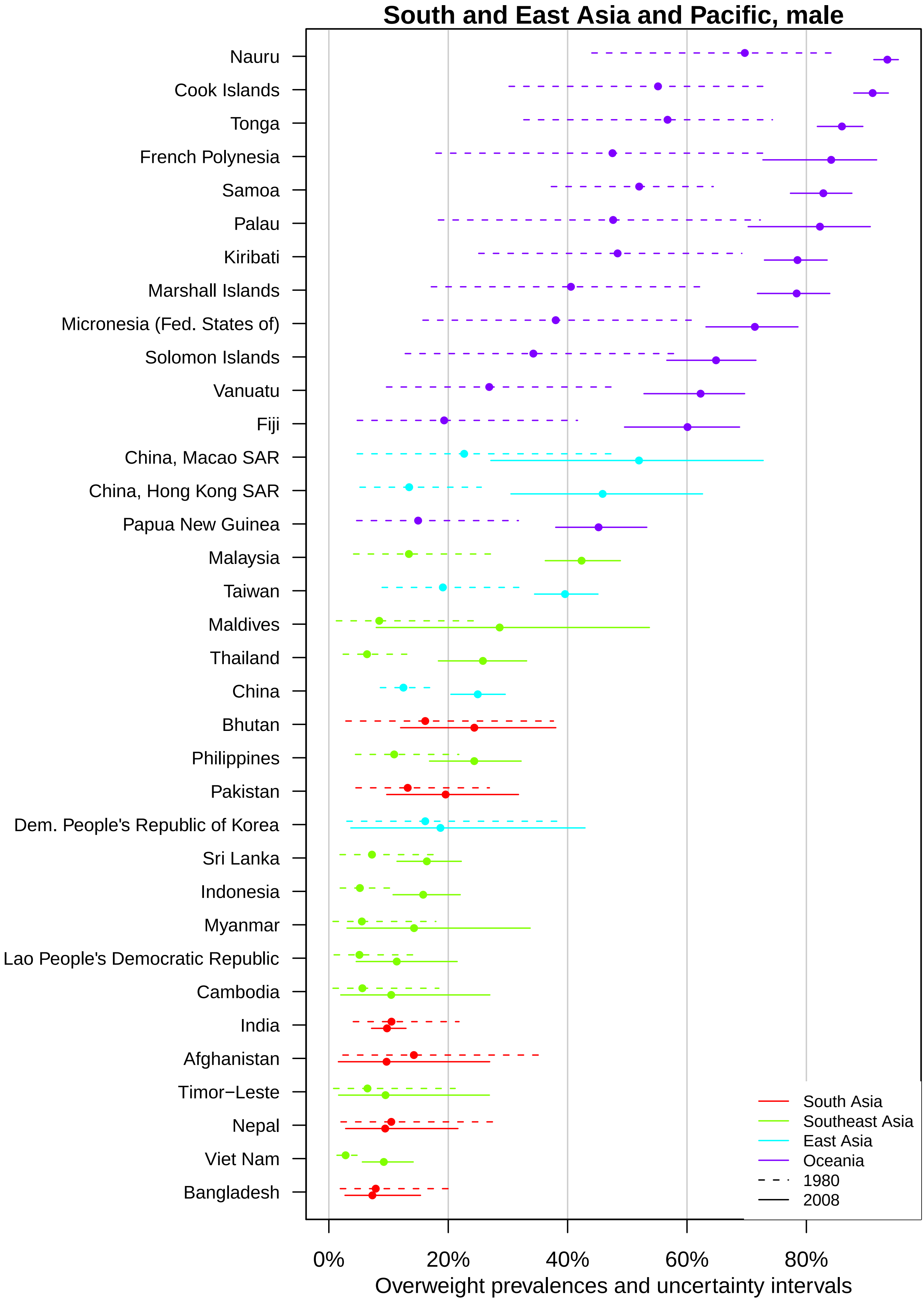
South and East Asia and Pacific, male
Nauru
Cook Islands
Tonga
French Polynesia
Samoa
Palau
Kiribati
Marshall Islands
Micronesia (Fed. States of)
Solomon Islands
Vanuatu
Fiji
China, Macao SAR
China, Hong Kong SAR
Papua New Guinea
Malaysia
Taiwan
Maldives
Thailand
China
Bhutan
Philippines
Pakistan
Dem. People's Republic of Korea
Sri Lanka
Indonesia
Myanmar
Lao People's Democratic Republic
Cambodia
India
Afghanistan
Timor−Leste
Nepal
Viet Nam
Bangladesh
South Asia
Southeast Asia
East Asia
Oceania
1980
2008
0%
20%
40%
60%
80%
Overweight prevalences and uncertainty intervals

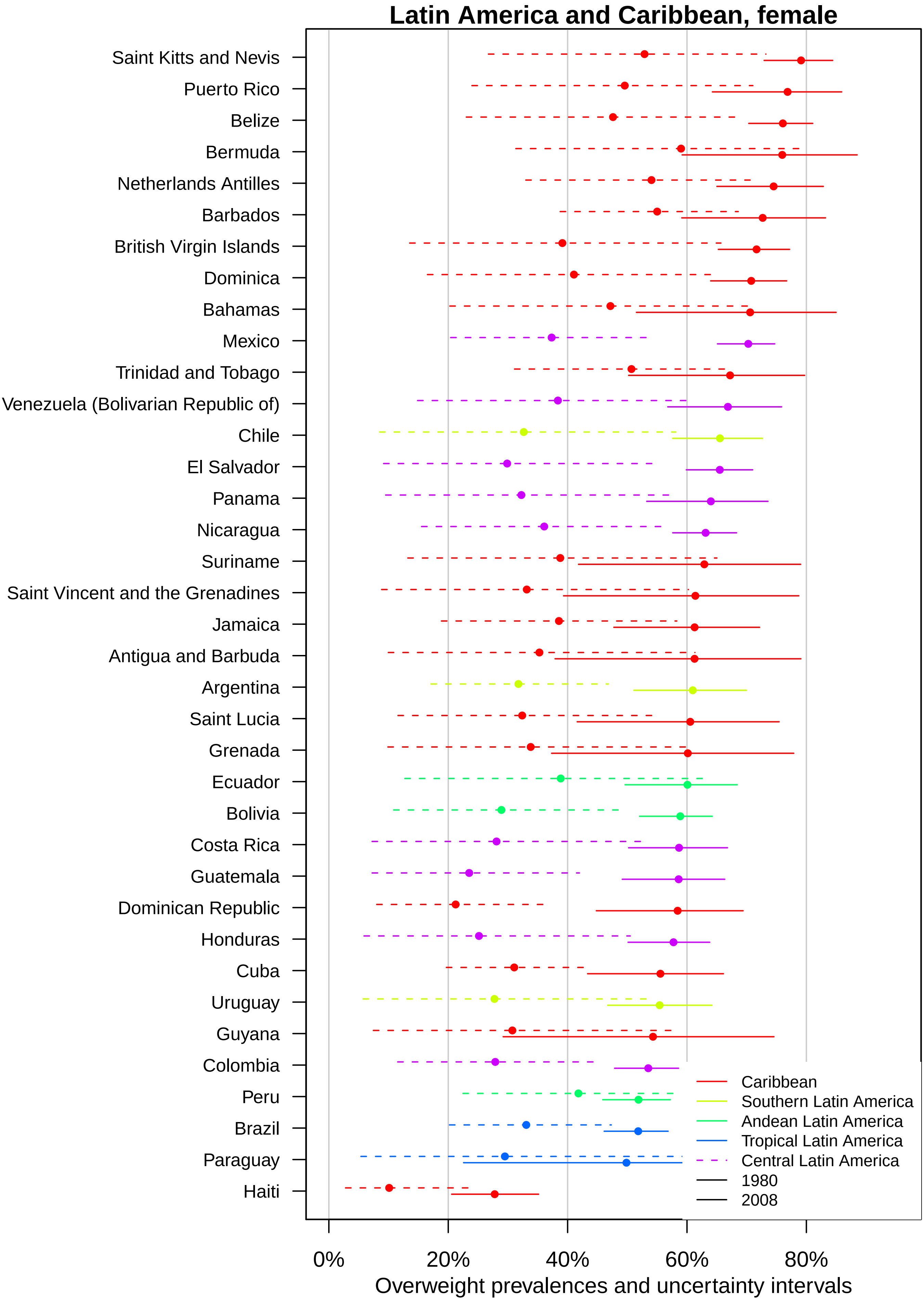

Latin America and Caribbean, female
Saint Kitts and Nevis
Puerto Rico
Belize
Bermuda
Netherlands Antilles
Barbados
British Virgin Islands
Dominica
Bahamas
Mexico
Trinidad and Tobago
Venezuela (Bolivarian Republic of)
Chile
El Salvador
Panama
Nicaragua
Suriname
Saint Vincent and the Grenadines
Jamaica
Antigua and Barbuda
Argentina
Saint Lucia
Grenada
Ecuador
Bolivia
Costa Rica
Guatemala
Dominican Republic
Honduras
Cuba
Uruguay
Guyana
Colombia
Peru
Brazil
Paraguay
Haiti
Caribbean
Southern Latin America
Andean Latin America
Tropical Latin America
Central Latin America
1980
2008
0%
20%
40%
60%
80%
Overweight prevalences and uncertainty intervals

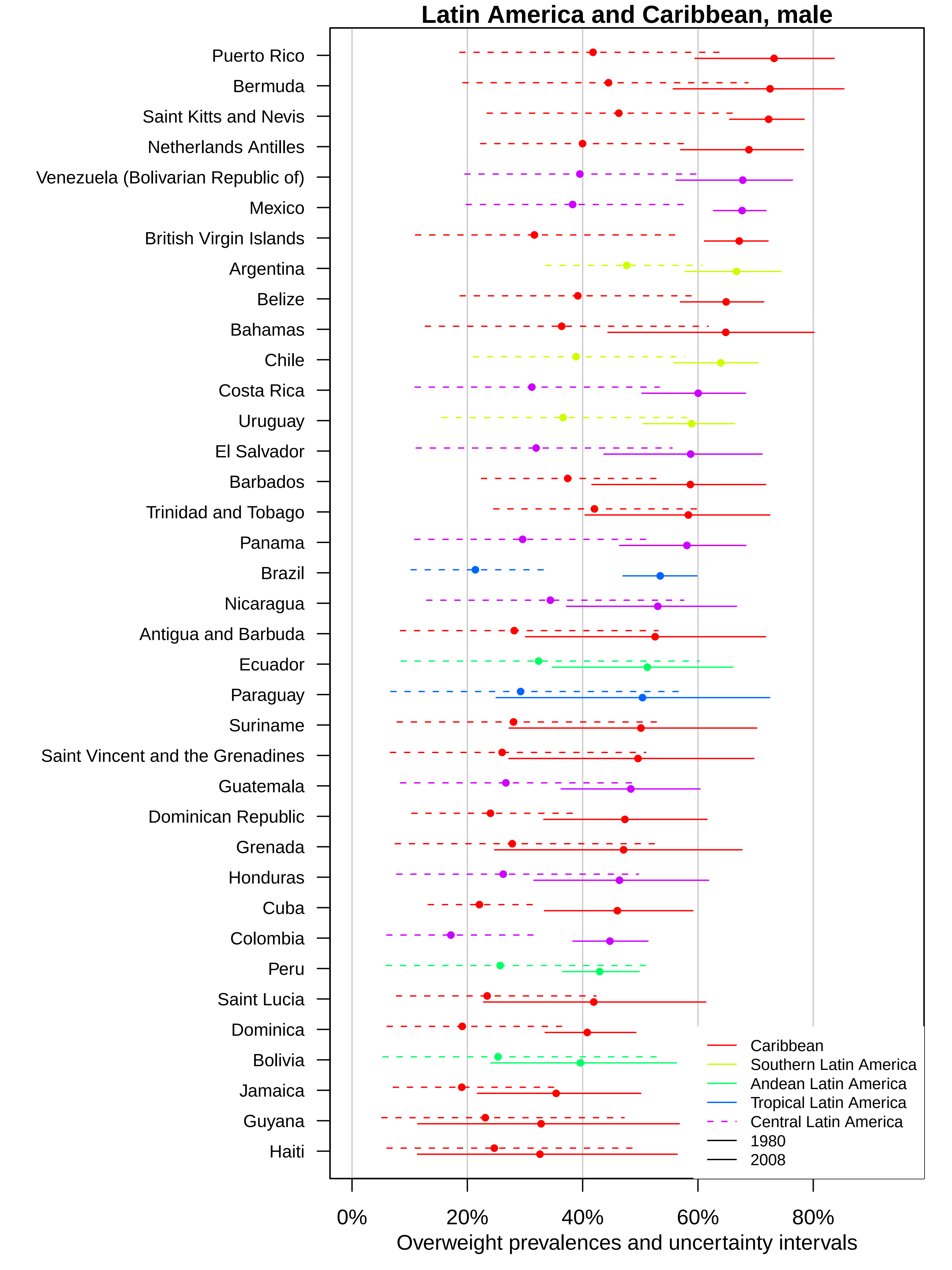
Latin America and Caribbean, male
Puerto Rico
Bermuda
Saint Kitts and Nevis
Netherlands Antilles
Venezuela (Bolivarian Republic of)
Mexico
British Virgin Islands
Argentina
Belize
Bahamas
Chile
Costa Rica
Uruguay
El Salvador
Barbados
Trinidad and Tobago
Panama
Brazil
Nicaragua
Antigua and Barbuda
Ecuador
Paraguay
Suriname
Saint Vincent and the Grenadines
Guatemala
Dominican Republic
Grenada
Honduras
Cuba
Colombia
Peru
Saint Lucia
Dominica
Bolivia
Jamaica
Guyana
Haiti
Caribbean
Southern Latin America
Andean Latin America
Tropical Latin America
Central Latin America
1980
2008
0%
20%
40%
60%
80%
Overweight prevalences and uncertainty intervals

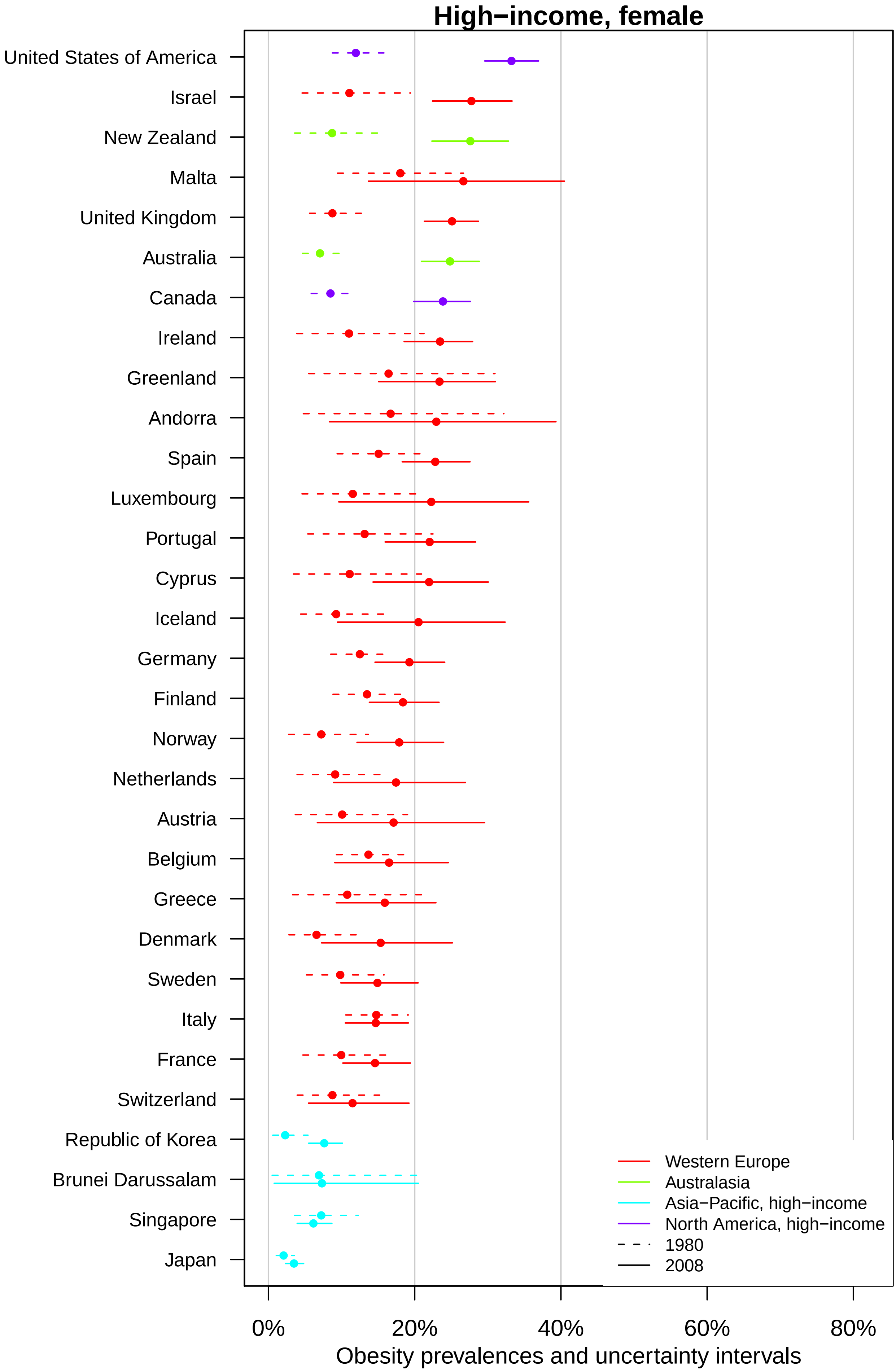

High−income, female
United States of America
Israel
New Zealand
Malta
United Kingdom
Australia
Canada
Ireland
Greenland
Andorra
Spain
Luxembourg
Portugal
Cyprus
Iceland
Germany
Finland
Norway
Netherlands
Austria
Belgium
Greece
Denmark
Sweden
Italy
France
Switzerland
Republic of Korea
Brunei Darussalam
Singapore
Japan
Western Europe
Australasia
Asia−Pacific, high−income
North America, high−income
1980
2008
0%
20%
40%
60%
80%
Obesity prevalences and uncertainty intervals

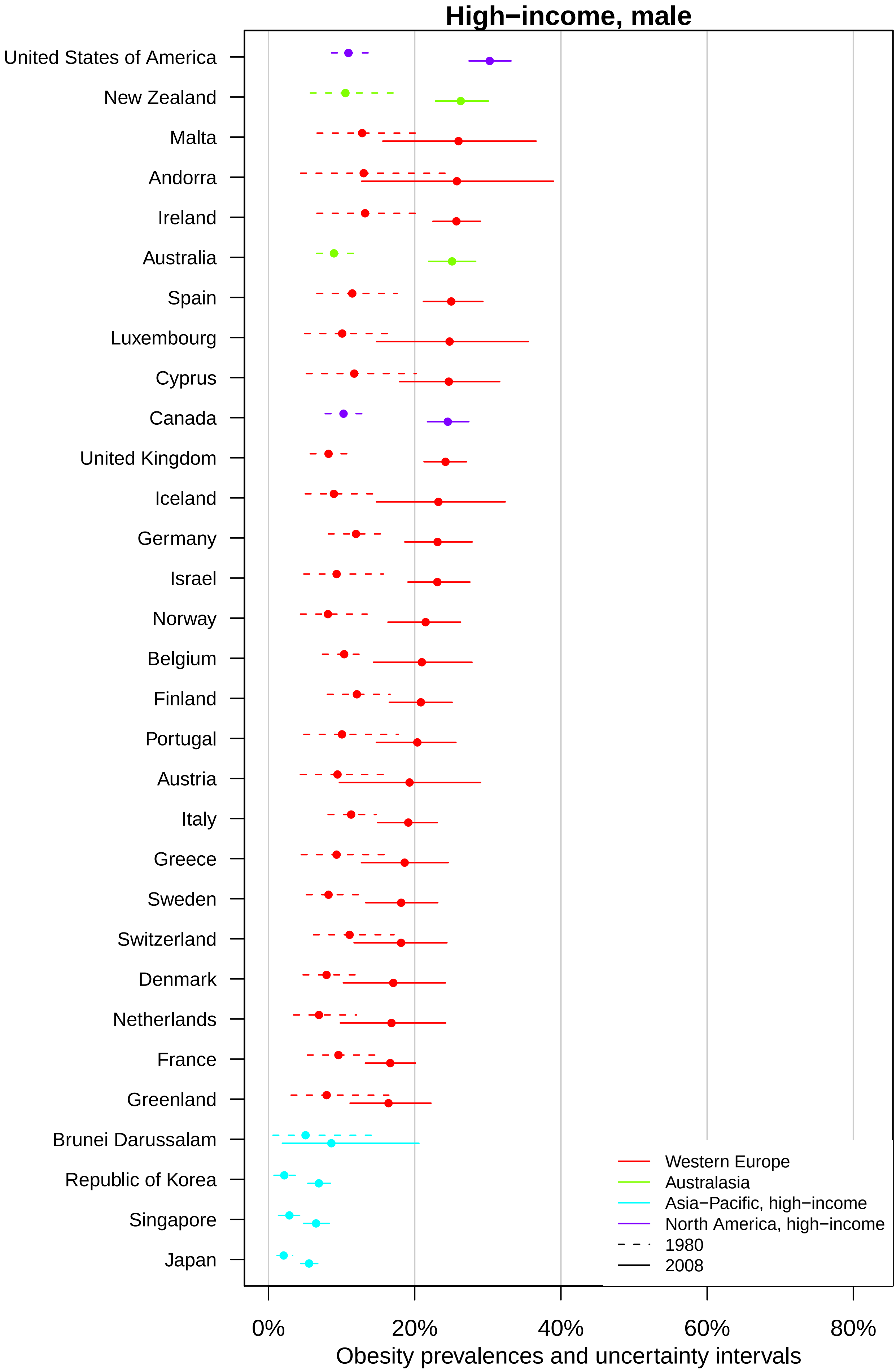

High−income, male
United States of America
New Zealand
Malta
Andorra
Ireland
Australia
Spain
Luxembourg
Cyprus
Canada
United Kingdom
Iceland
Germany
Israel
Norway
Belgium
Finland
Portugal
Austria
Italy
Greece
Sweden
Switzerland
Denmark
Netherlands
France
Greenland
Brunei Darussalam
Republic of Korea
Singapore
Japan
Western Europe
Australasia
Asia−Pacific, high−income
North America, high−income
1980
2008
0%
20%
40%
60%
80%
Obesity prevalences and uncertainty intervals

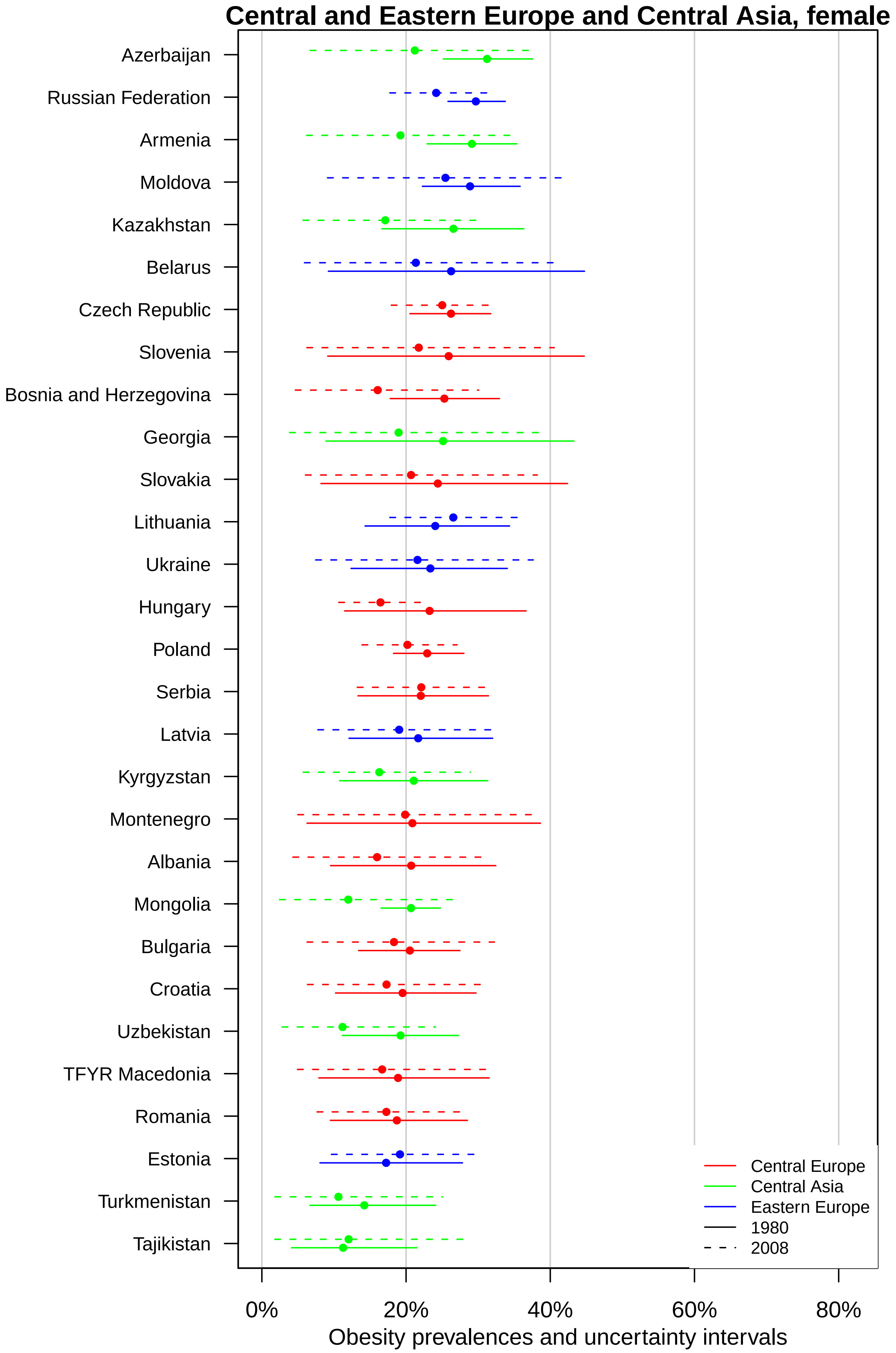

Central and Eastern Europe and Central Asia, female
Azerbaijan
Russian Federation
Armenia
Moldova
Kazakhstan
Belarus
Czech Republic
Slovenia
Bosnia and Herzegovina
Georgia
Slovakia
Lithuania
Ukraine
Hungary
Poland
Serbia
Latvia
Kyrgyzstan
Montenegro
Albania
Mongolia
Bulgaria
Croatia
Uzbekistan
TFYR Macedonia
Romania
Estonia
Turkmenistan
Tajikistan
Central Europe
Central Asia
Eastern Europe
1980
2008
0%
20%
40%
60%
80%
Obesity prevalences and uncertainty intervals

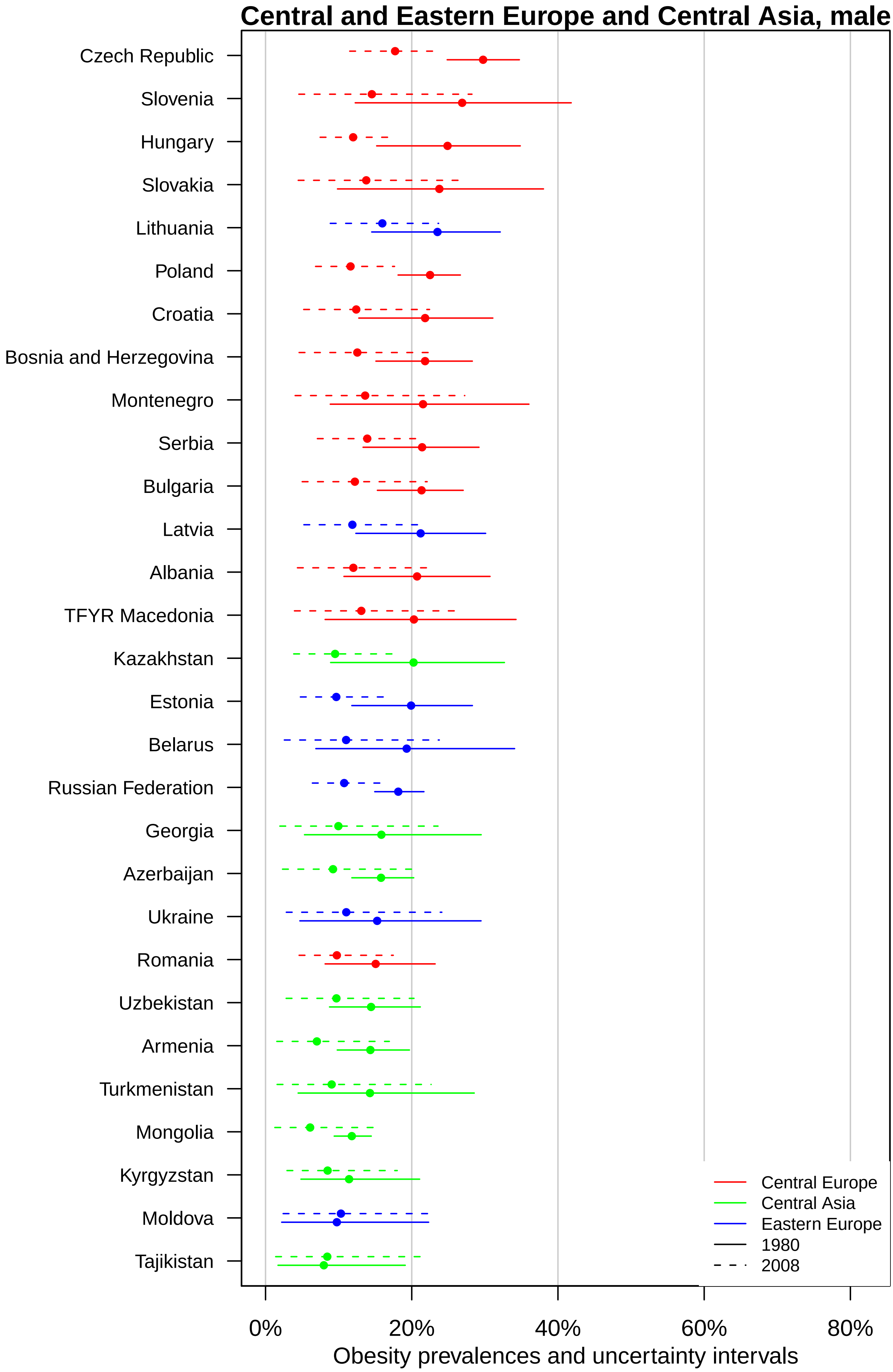

Central and Eastern Europe and Central Asia, male
Czech Republic
Slovenia
Hungary
Slovakia
Lithuania
Poland
Croatia
Bosnia and Herzegovina
Montenegro
Serbia
Bulgaria
Latvia
Albania
TFYR Macedonia
Kazakhstan
Estonia
Belarus
Russian Federation
Georgia
Azerbaijan
Ukraine
Romania
Uzbekistan
Armenia
Turkmenistan
Mongolia
Kyrgyzstan
Moldova
Tajikistan
Central Europe
Central Asia
Eastern Europe
1980
2008
0%
20%
40%
60%
80%
Obesity prevalences and uncertainty intervals

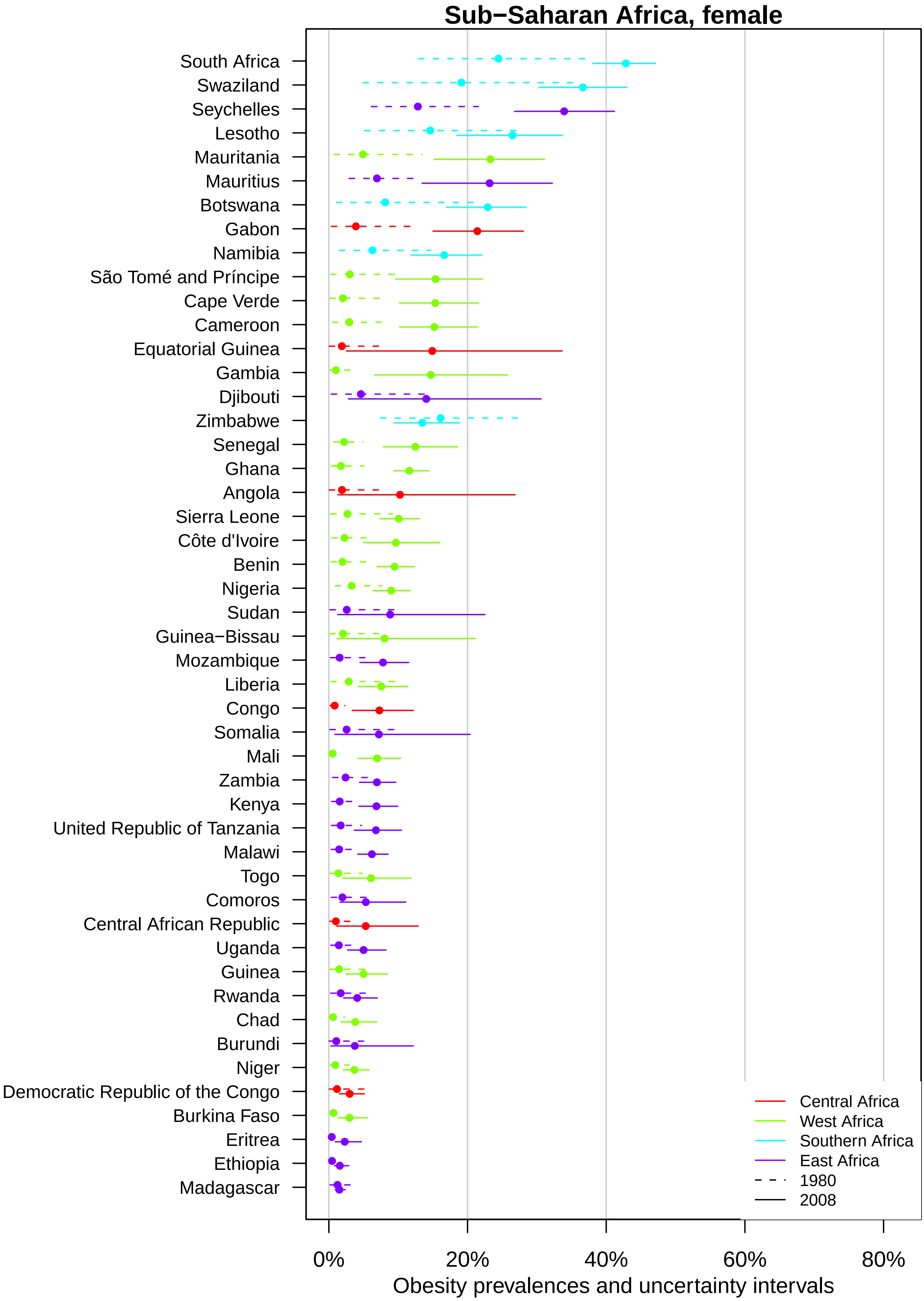
Sub−Saharan Africa, female
South Africa
Swaziland
Seychelles
Lesotho
Mauritania
Mauritius
Botswana
Gabon
Namibia
São Tomé and Príncipe
Cape Verde
Cameroon
Equatorial Guinea
Gambia
Djibouti
Zimbabwe
Senegal
Ghana
Angola
Sierra Leone
Côte d'Ivoire
Benin
Nigeria
Sudan
Guinea−Bissau
Mozambique
Liberia
Congo
Somalia
Mali
Zambia
Kenya
United Republic of Tanzania
Malawi
Togo
Comoros
Central African Republic
Uganda
Guinea
Rwanda
Chad
Burundi
Niger
Democratic Republic of the Congo
Burkina Faso
Eritrea
Ethiopia
Madagascar
Central Africa
West Africa
Southern Africa
East Africa
1980
2008
0%
20%
40%
60%
80%
Obesity prevalences and uncertainty intervals

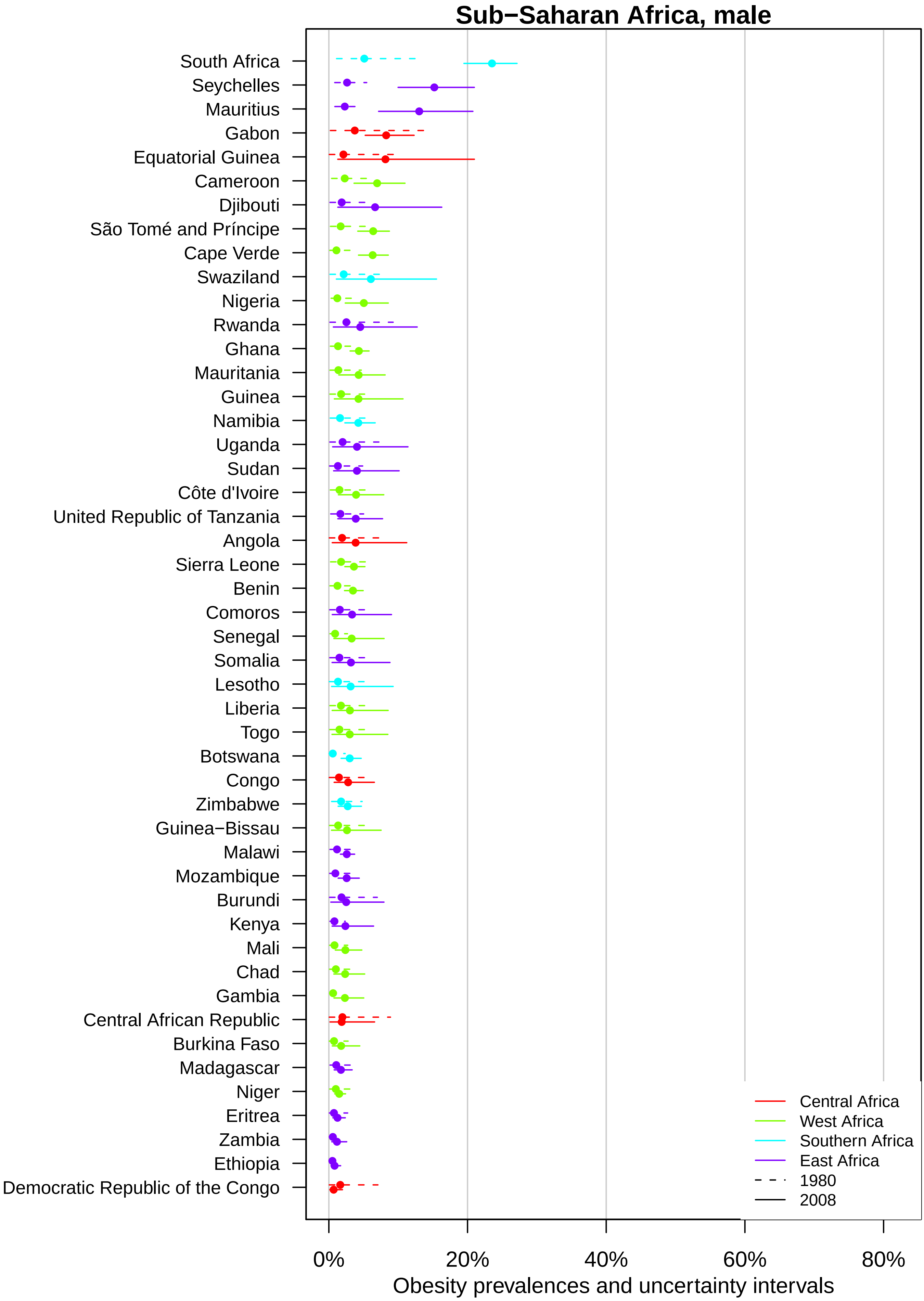

Sub−Saharan Africa, male
South Africa
Seychelles
Mauritius
Gabon
Equatorial Guinea
Cameroon
Djibouti
São Tomé and Príncipe
Cape Verde
Swaziland
Nigeria
Rwanda
Ghana
Mauritania
Guinea
Namibia
Uganda
Sudan
Côte d'Ivoire
United Republic of Tanzania
Angola
Sierra Leone
Benin
Comoros
Senegal
Somalia
Lesotho
Liberia
Togo
Botswana
Congo
Zimbabwe
Guinea−Bissau
Malawi
Mozambique
Burundi
Kenya
Mali
Chad
Gambia
Central African Republic
Burkina Faso
Madagascar
Niger
Eritrea
Zambia
Ethiopia
Democratic Republic of the Congo
Central Africa
West Africa
Southern Africa
East Africa
1980
2008
0%
20%
40%
60%
80%
Obesity prevalences and uncertainty intervals

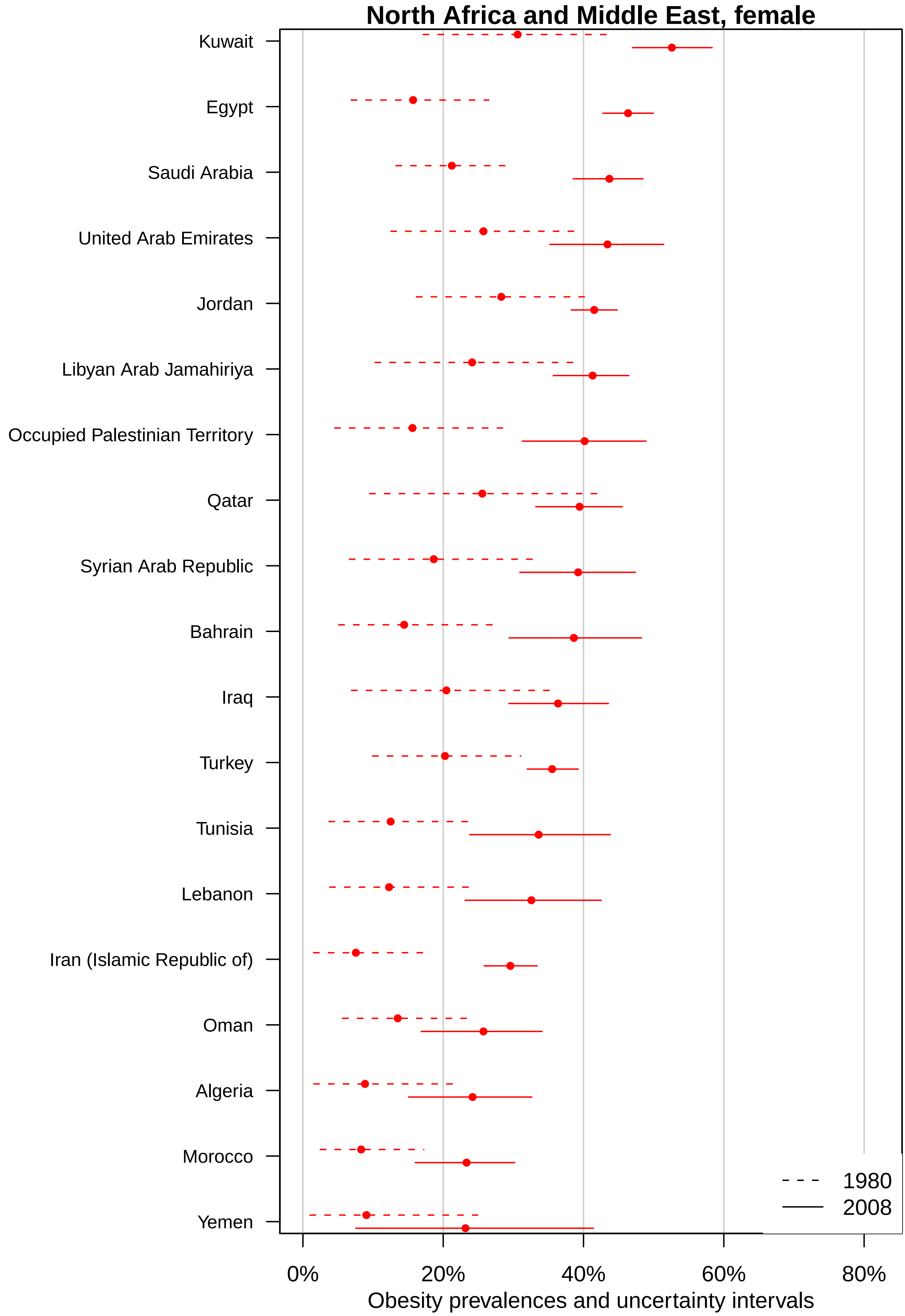
North Africa and Middle East, female
Kuwait
Egypt
Saudi Arabia
United Arab Emirates
Jordan
Libyan Arab Jamahiriya
Occupied Palestinian Territory
Qatar
Syrian Arab Republic
Bahrain
Iraq
Turkey
Tunisia
Lebanon
Iran (Islamic Republic of)
Oman
Algeria
Morocco
Yemen
1980
2008
0%
20%
40%
60%
80%
Obesity prevalences and uncertainty intervals

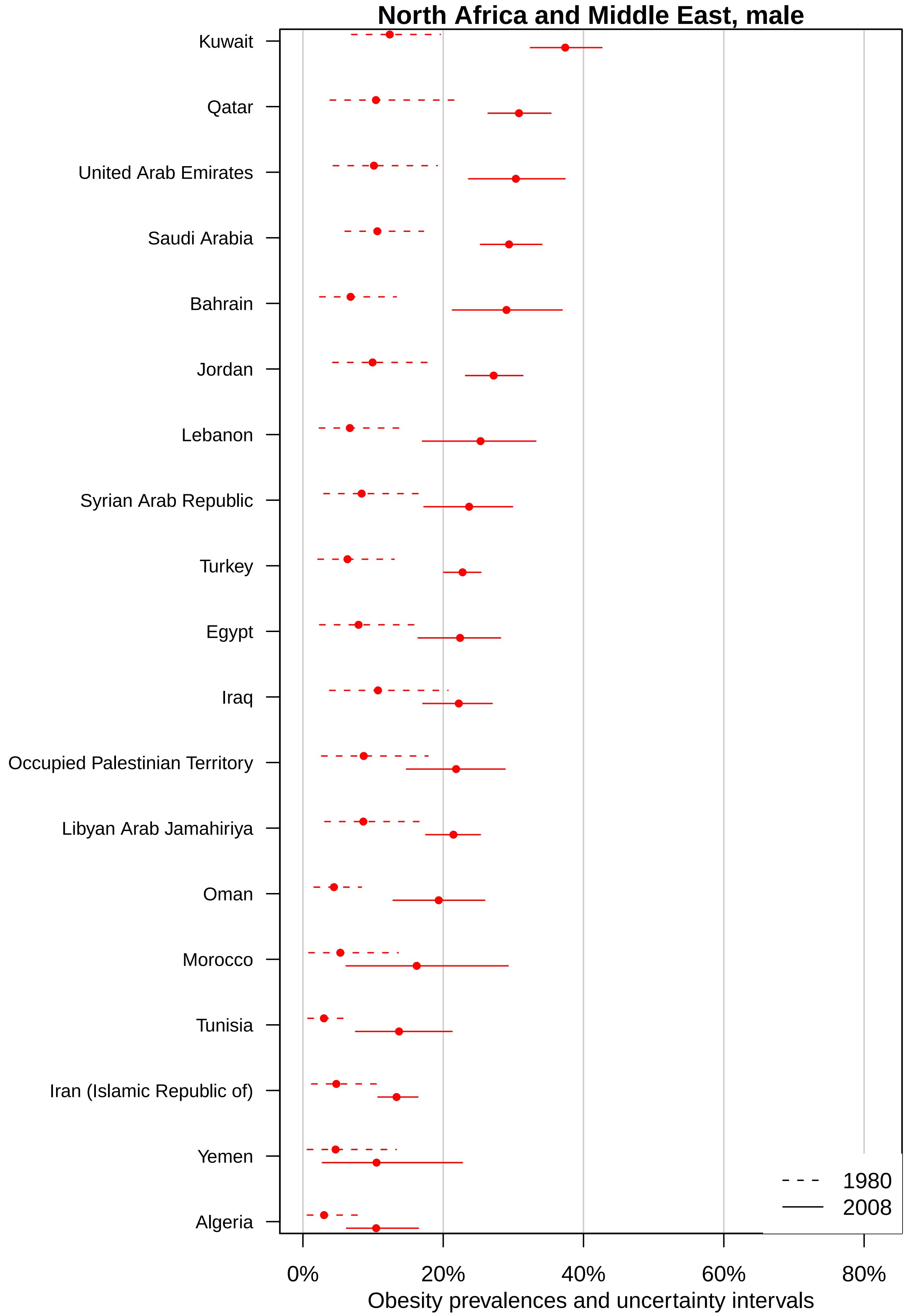
North Africa and Middle East, male
Kuwait
Qatar
United Arab Emirates
Saudi Arabia
Bahrain
Jordan
Lebanon
Syrian Arab Republic
Turkey
Egypt
Iraq
Occupied Palestinian Territory
Libyan Arab Jamahiriya
Oman
Morocco
Tunisia
Iran (Islamic Republic of)
Yemen
Algeria
1980
2008
0%
20%
40%
60%
80%
Obesity prevalences and uncertainty intervals

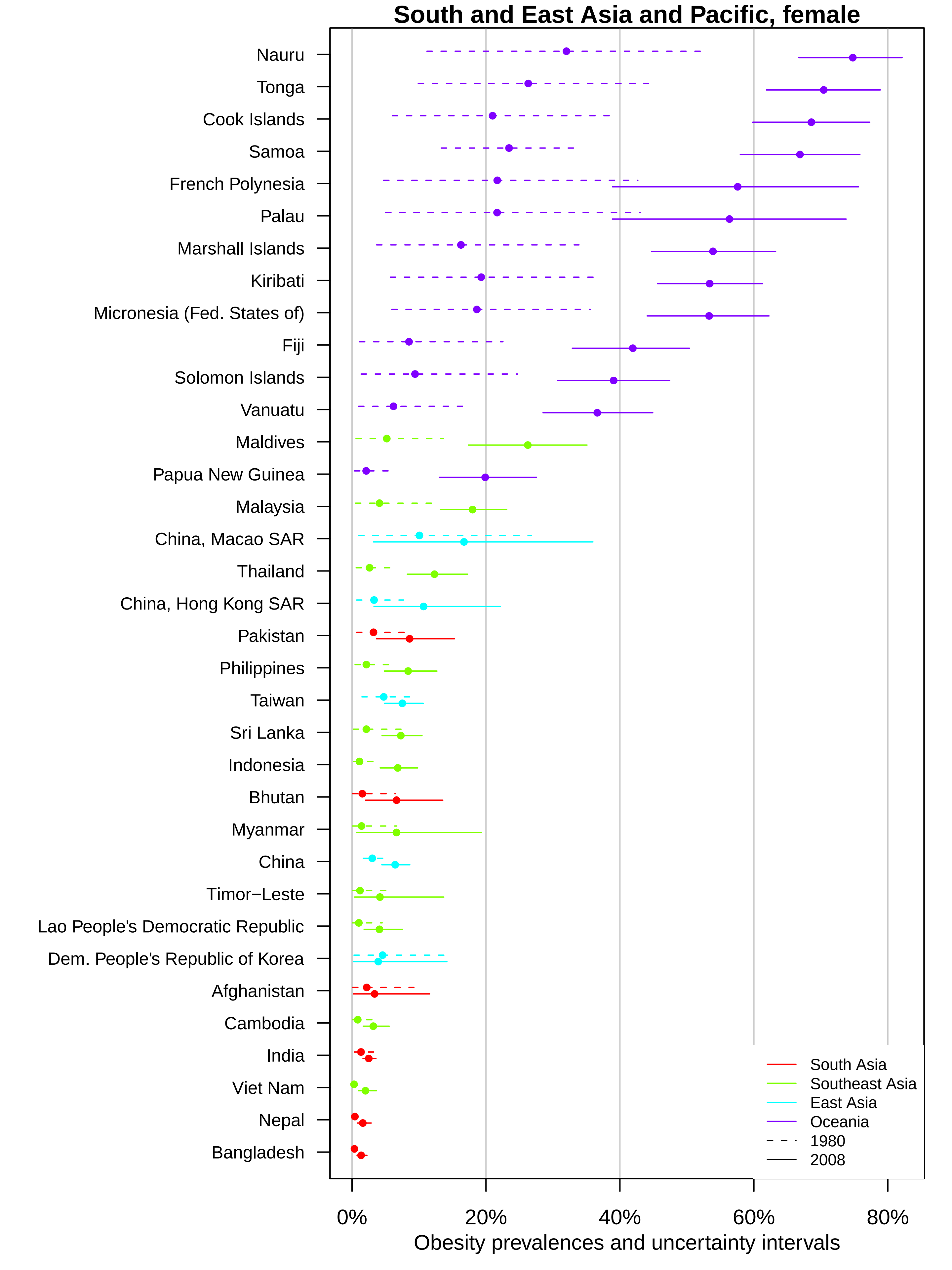
South and East Asia and Pacific, female
Nauru
Tonga
Cook Islands
Samoa
French Polynesia
Palau
Marshall Islands
Kiribati
Micronesia (Fed. States of)
Fiji
Solomon Islands
Vanuatu
Maldives
Papua New Guinea
Malaysia
China, Macao SAR
Thailand
China, Hong Kong SAR
Pakistan
Philippines
Taiwan
Sri Lanka
Indonesia
Bhutan
Myanmar
China
Timor−Leste
Lao People's Democratic Republic
Dem. People's Republic of Korea
Afghanistan
Cambodia
India
Viet Nam
Nepal
Bangladesh
0%
20%
40%
60%
80%
Obesity prevalences and uncertainty intervals
South Asia
Southeast Asia
East Asia
Oceania
1980
2008

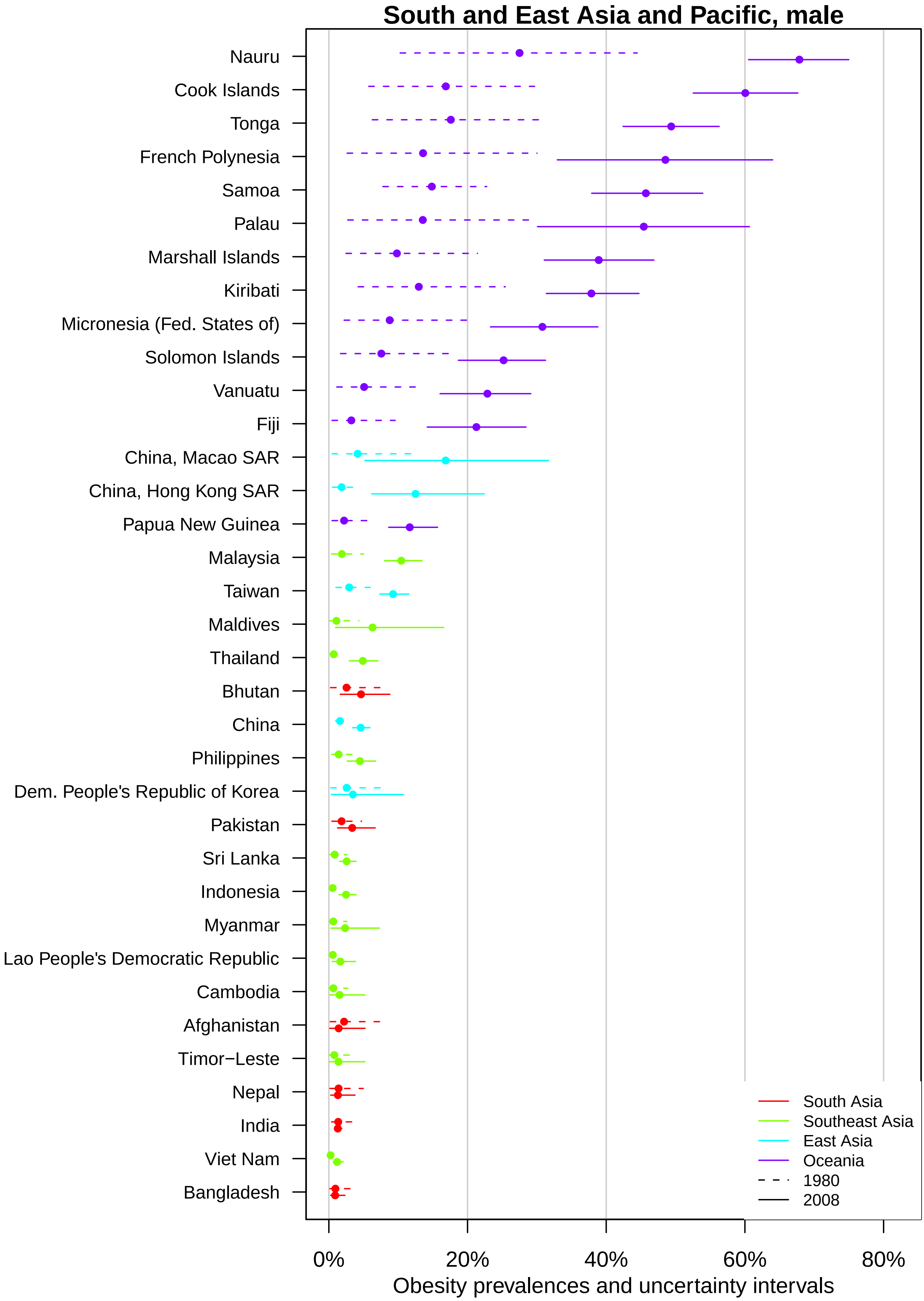
South and East Asia and Pacific, male
Nauru
Cook Islands
Tonga
French Polynesia
Samoa
Palau
Marshall Islands
Kiribati
Micronesia (Fed. States of)
Solomon Islands
Vanuatu
Fiji
China, Macao SAR
China, Hong Kong SAR
Papua New Guinea
Malaysia
Taiwan
Maldives
Thailand
Bhutan
China
Philippines
Dem. People's Republic of Korea
Pakistan
Sri Lanka
Indonesia
Myanmar
Lao People's Democratic Republic
Cambodia
Afghanistan
Timor−Leste
Nepal
India
Viet Nam
Bangladesh
South Asia
Southeast Asia
East Asia
Oceania
1980
2008
0%
20%
40%
60%
80%
Obesity prevalences and uncertainty intervals

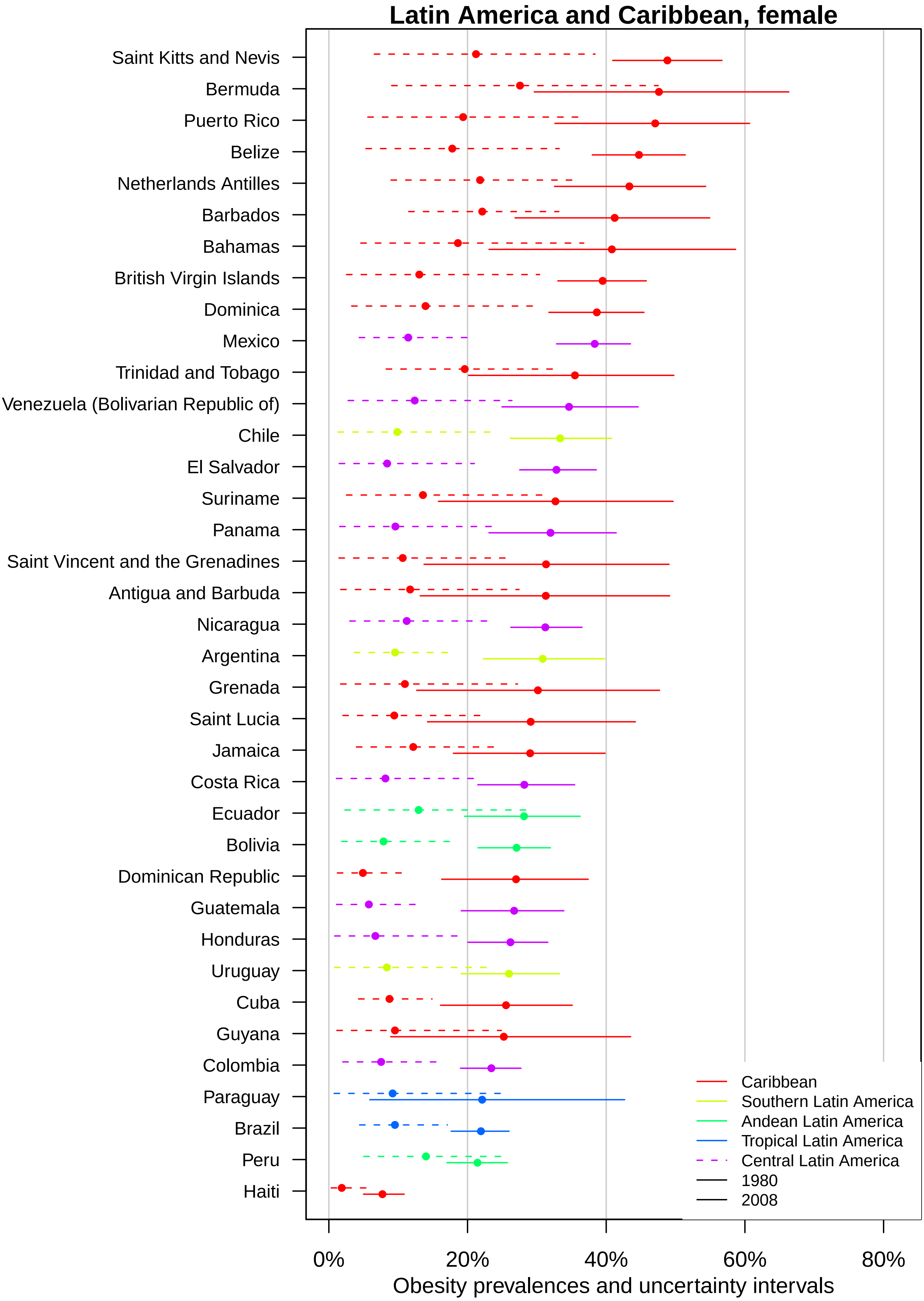

Latin America and Caribbean, female
Saint Kitts and Nevis
Bermuda
Puerto Rico
Belize
Netherlands Antilles
Barbados
Bahamas
British Virgin Islands
Dominica
Mexico
Trinidad and Tobago
Venezuela (Bolivarian Republic of)
Chile
El Salvador
Suriname
Panama
Saint Vincent and the Grenadines
Antigua and Barbuda
Nicaragua
Argentina
Grenada
Saint Lucia
Jamaica
Costa Rica
Ecuador
Bolivia
Dominican Republic
Guatemala
Honduras
Uruguay
Cuba
Guyana
Colombia
Paraguay
Brazil
Peru
Haiti
Caribbean
Southern Latin America
Andean Latin America
Tropical Latin America
Central Latin America
1980
2008
0%
20%
40%
60%
80%
Obesity prevalences and uncertainty intervals

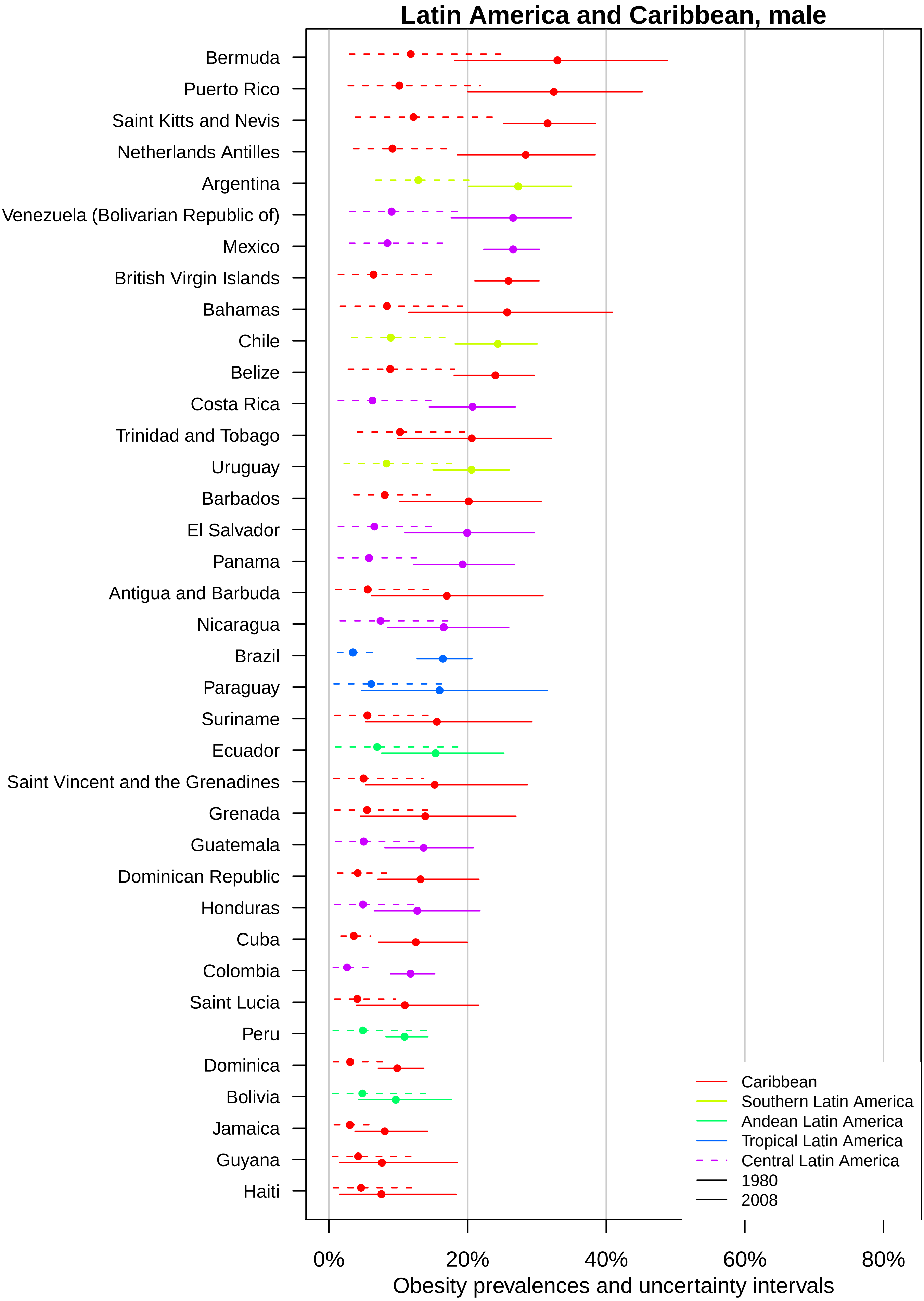
Latin America and Caribbean, male
Bermuda
Puerto Rico
Saint Kitts and Nevis
Netherlands Antilles
Argentina
Venezuela (Bolivarian Republic of)
Mexico
British Virgin Islands
Bahamas
Chile
Belize
Costa Rica
Trinidad and Tobago
Uruguay
Barbados
El Salvador
Panama
Antigua and Barbuda
Nicaragua
Brazil
Paraguay
Suriname
Ecuador
Saint Vincent and the Grenadines
Grenada
Guatemala
Dominican Republic
Honduras
Cuba
Colombia
Saint Lucia
Peru
Dominica
Bolivia
Jamaica
Guyana
Haiti
Caribbean
Southern Latin America
Andean Latin America
Tropical Latin America
Central Latin America
1980
2008
0%
20%
40%
60%
80%
Obesity prevalences and uncertainty intervals
