# Supplementary material for: National, regional, and global trends in adult overweight and obesity prevalences
Source: Popul Health Metr. 2012 Nov 20;10:22. doi: 10.1186/1478-7954-10-22 (PMC3543235; doi:10.1186/1478-7954-10-22)

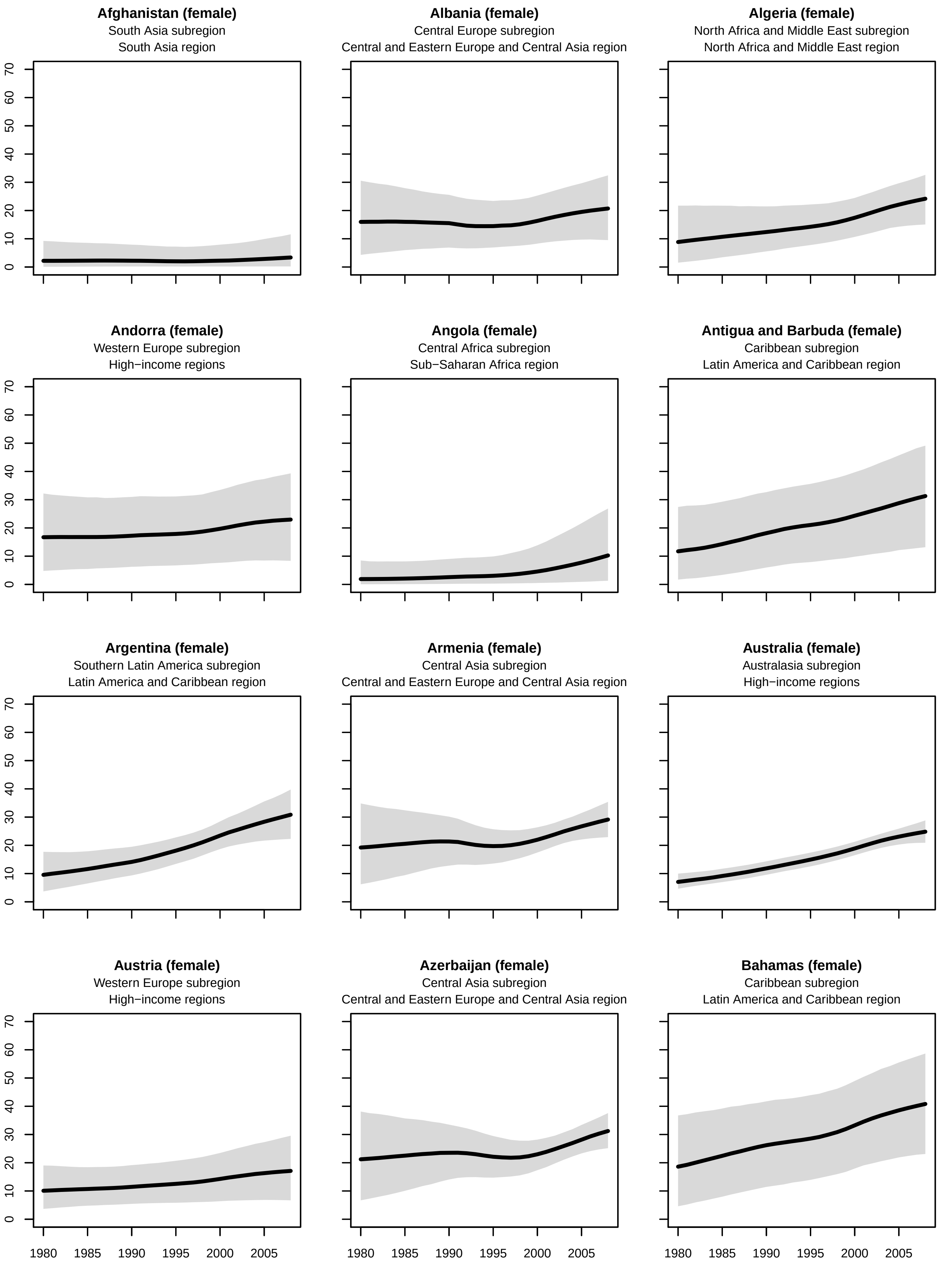
Afghanistan (female)
South Asia subregion
South Asia region
Albania (female)
Central Europe subregion
Central and Eastern Europe and Central Asia region
Algeria (female)
North Africa and Middle East subregion
North Africa and Middle East region
Andorra (female)
Western Europe subregion
High−income regions
Angola (female)
Central Africa subregion
Sub−Saharan Africa region
Antigua and Barbuda (female)
Caribbean subregion
Latin America and Caribbean region
Argentina (female)
Southern Latin America subregion
Latin America and Caribbean region
Armenia (female)
Central Asia subregion
Central and Eastern Europe and Central Asia region
Australia (female)
Australasia subregion
High−income regions
Austria (female)
Western Europe subregion
High−income regions
Azerbaijan (female)
Central Asia subregion
Central and Eastern Europe and Central Asia region
Bahamas (female)
Caribbean subregion
Latin America and Caribbean region
0
10
20
30
40
50
60
70
1980
1985
1990
1995
2000
2005

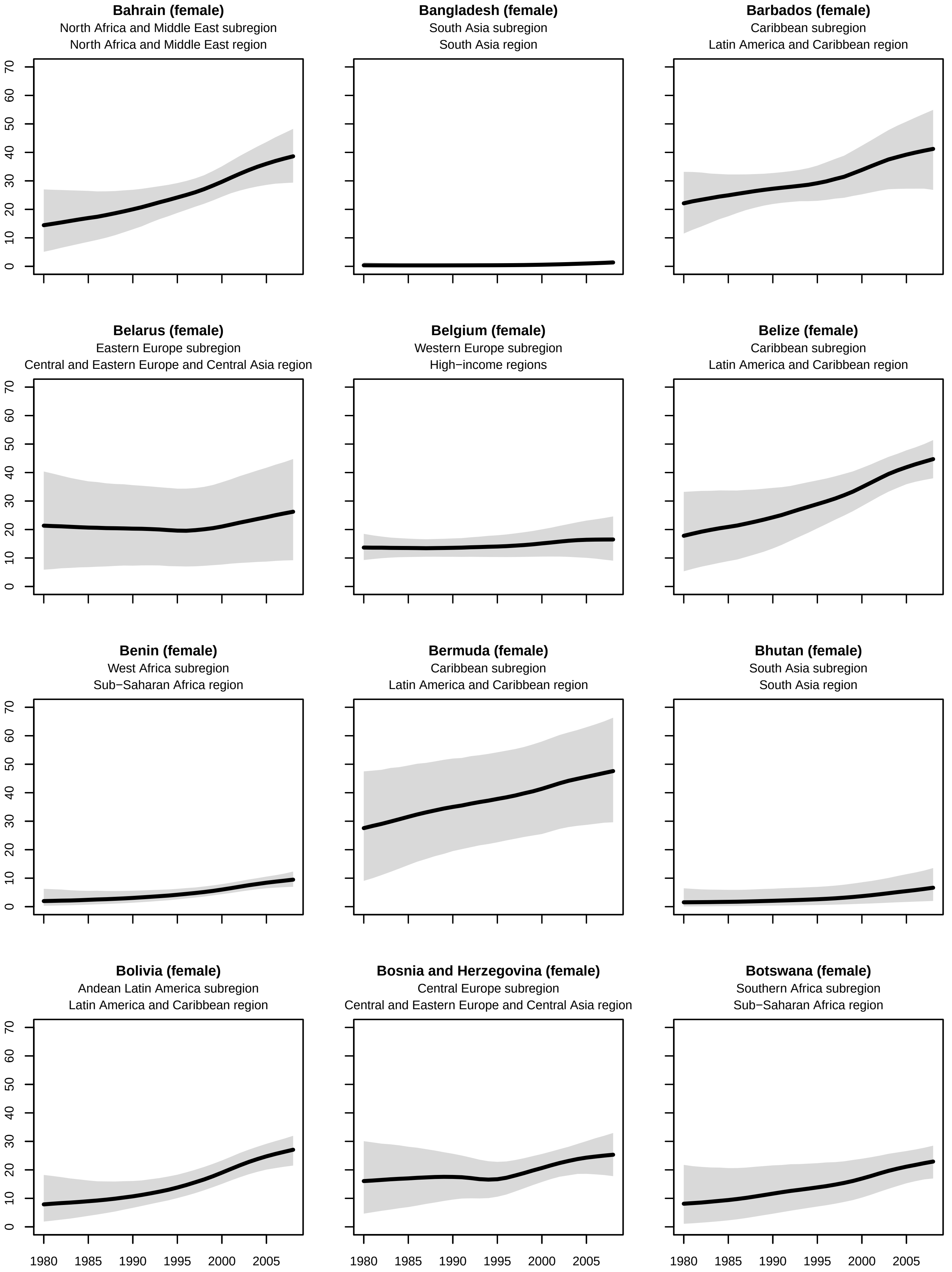
Bahrain (female)
North Africa and Middle East subregion
North Africa and Middle East region
Bangladesh (female)
South Asia subregion
South Asia region
Barbados (female)
Caribbean subregion
Latin America and Caribbean region
Belarus (female)
Eastern Europe subregion
Central and Eastern Europe and Central Asia region
Belgium (female)
Western Europe subregion
High−income regions
Belize (female)
Caribbean subregion
Latin America and Caribbean region
Benin (female)
West Africa subregion
Sub−Saharan Africa region
Bermuda (female)
Caribbean subregion
Latin America and Caribbean region
Bhutan (female)
South Asia subregion
South Asia region
Bolivia (female)
Andean Latin America subregion
Latin America and Caribbean region
Bosnia and Herzegovina (female)
Central Europe subregion
Central and Eastern Europe and Central Asia region
Botswana (female)
Southern Africa subregion
Sub−Saharan Africa region
0 10 20 30 40 50 60 70
1980 1985 1990 1995 2000 2005

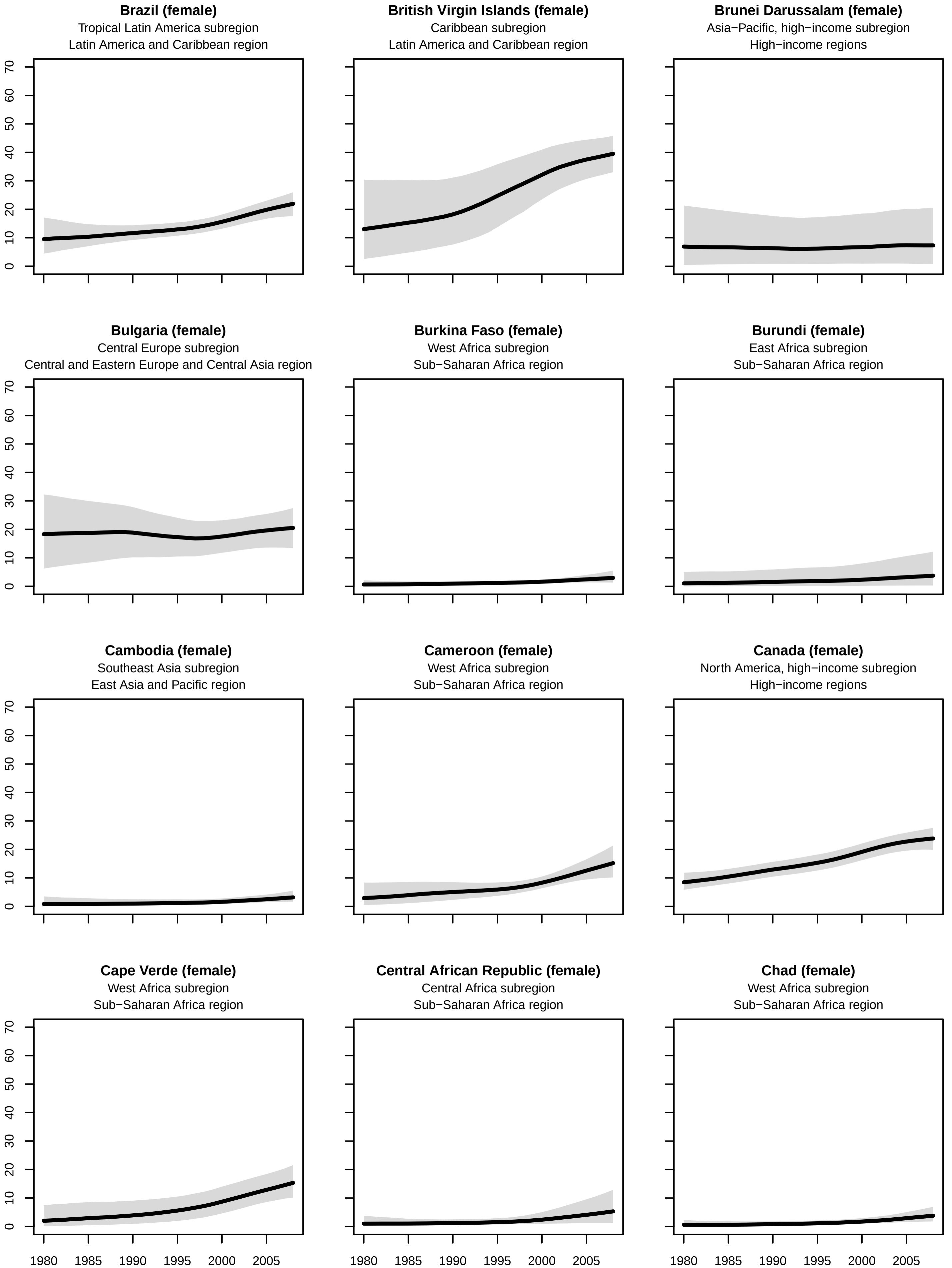
Brazil (female)
Tropical Latin America subregion
Latin America and Caribbean region
British Virgin Islands (female)
Caribbean subregion
Latin America and Caribbean region
Brunei Darussalam (female)
Asia−Pacific, high−income subregion
High−income regions
Bulgaria (female)
Central Europe subregion
Central and Eastern Europe and Central Asia region
Burkina Faso (female)
West Africa subregion
Sub−Saharan Africa region
Burundi (female)
East Africa subregion
Sub−Saharan Africa region
Cambodia (female)
Southeast Asia subregion
East Asia and Pacific region
Cameroon (female)
West Africa subregion
Sub−Saharan Africa region
Canada (female)
North America, high−income subregion
High−income regions
Cape Verde (female)
West Africa subregion
Sub−Saharan Africa region
Central African Republic (female)
Central Africa subregion
Sub−Saharan Africa region
Chad (female)
West Africa subregion
Sub−Saharan Africa region
0
10
20
30
40
50
60
70
1980
1985
1990
1995
2000
2005

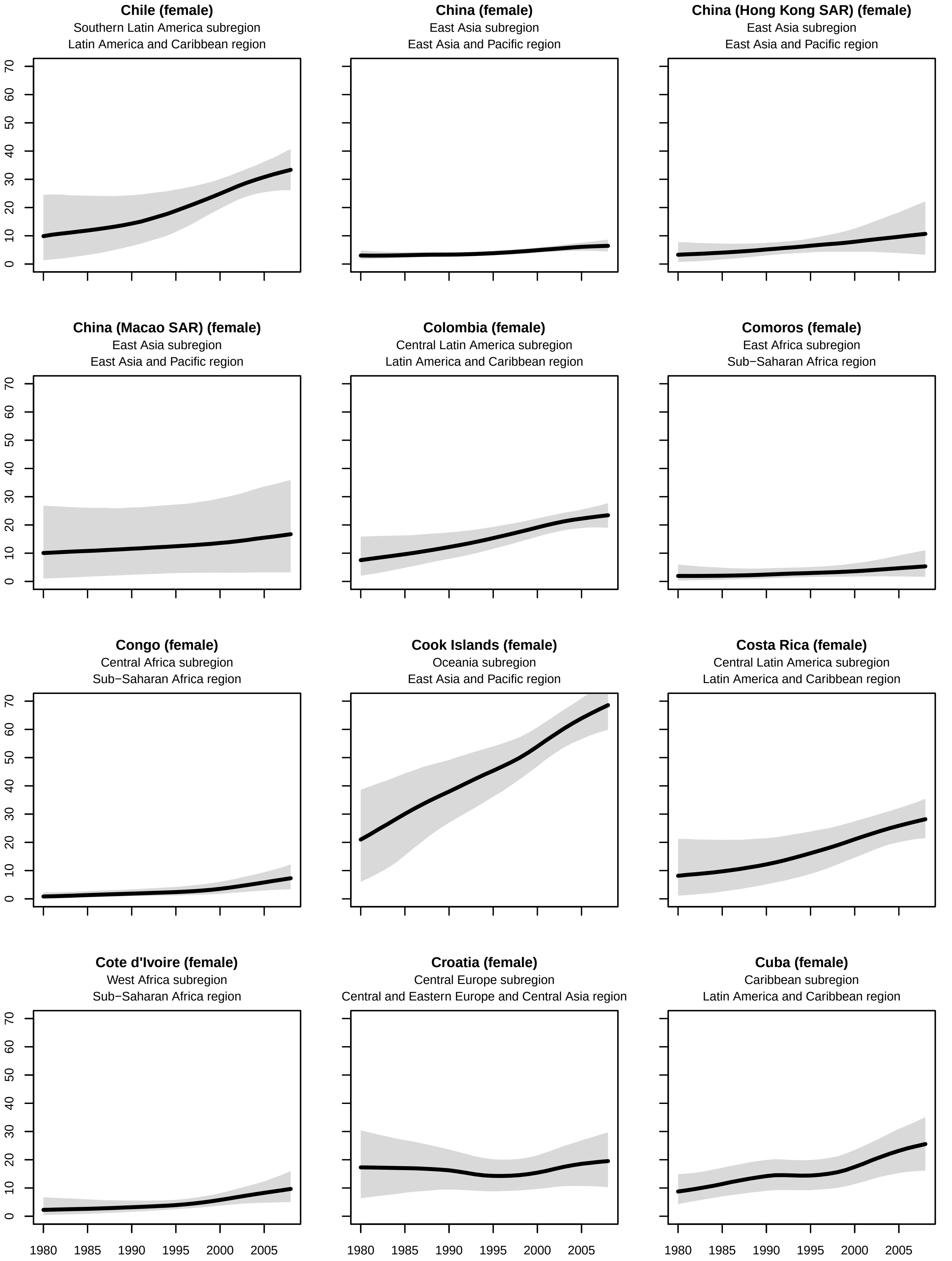

Chile (female)
Southern Latin America subregion
Latin America and Caribbean region
China (female)
East Asia subregion
East Asia and Pacific region
China (Hong Kong SAR) (female)
East Asia subregion
East Asia and Pacific region
China (Macao SAR) (female)
East Asia subregion
East Asia and Pacific region
Colombia (female)
Central Latin America subregion
Latin America and Caribbean region
Comoros (female)
East Africa subregion
Sub−Saharan Africa region
Congo (female)
Central Africa subregion
Sub−Saharan Africa region
Cook Islands (female)
Oceania subregion
East Asia and Pacific region
Costa Rica (female)
Central Latin America subregion
Latin America and Caribbean region
Cote d'Ivoire (female)
West Africa subregion
Sub−Saharan Africa region
Croatia (female)
Central Europe subregion
Central and Eastern Europe and Central Asia region
Cuba (female)
Caribbean subregion
Latin America and Caribbean region
0
10
20
30
40
50
60
70
1980
1985
1990
1995
2000
2005

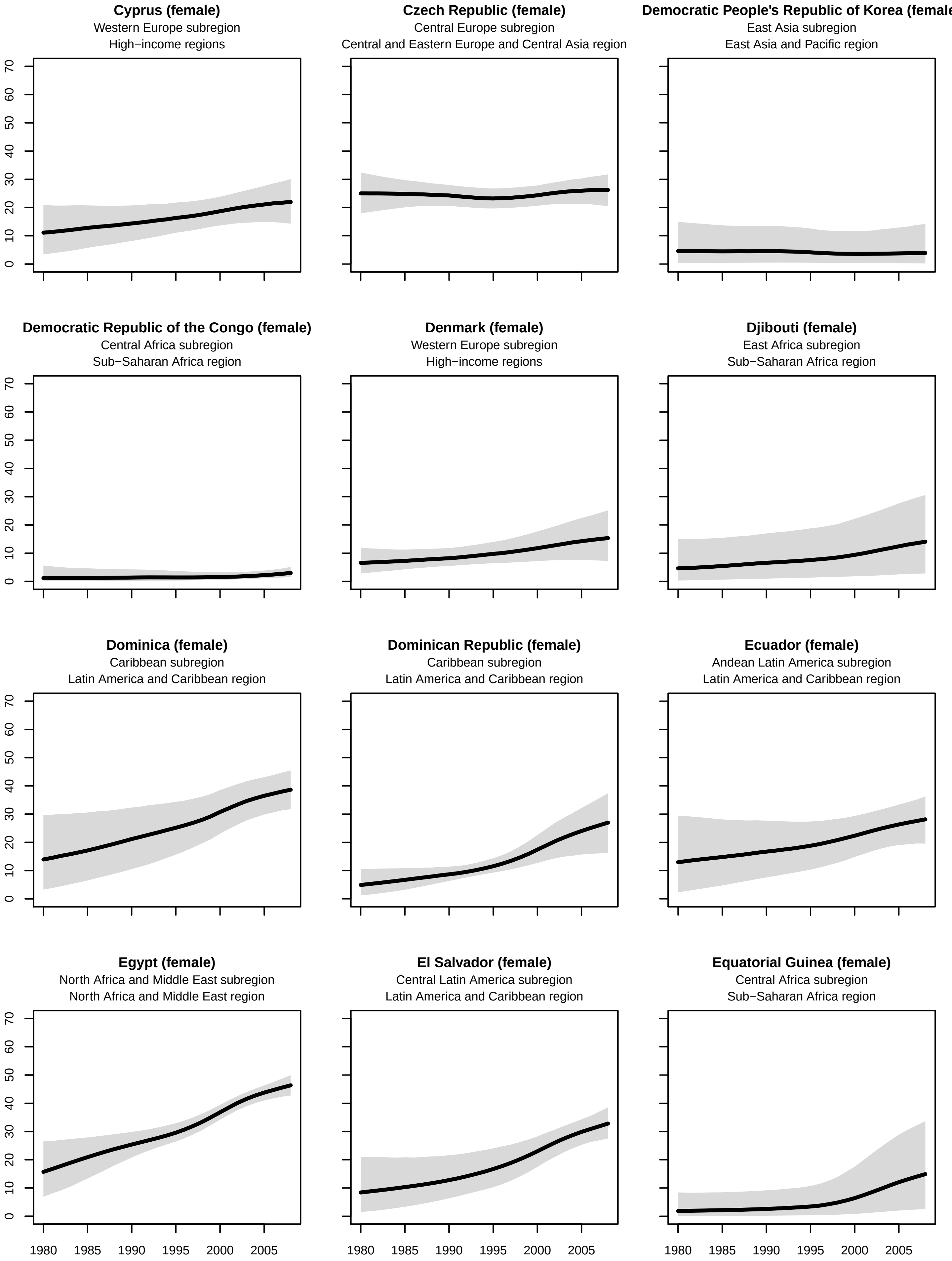
Cyprus (female)
Western Europe subregion
High−income regions
Czech Republic (female)
Central Europe subregion
Central and Eastern Europe and Central Asia region
Democratic People's Republic of Korea (female
East Asia subregion
East Asia and Pacific region
Democratic Republic of the Congo (female)
Central Africa subregion
Sub−Saharan Africa region
Denmark (female)
Western Europe subregion
High−income regions
Djibouti (female)
East Africa subregion
Sub−Saharan Africa region
Dominica (female)
Caribbean subregion
Latin America and Caribbean region
Dominican Republic (female)
Caribbean subregion
Latin America and Caribbean region
Ecuador (female)
Andean Latin America subregion
Latin America and Caribbean region
Egypt (female)
North Africa and Middle East subregion
North Africa and Middle East region
El Salvador (female)
Central Latin America subregion
Latin America and Caribbean region
Equatorial Guinea (female)
Central Africa subregion
Sub−Saharan Africa region
0 10 20 30 40 50 60 70
1980 1985 1990 1995 2000 2005

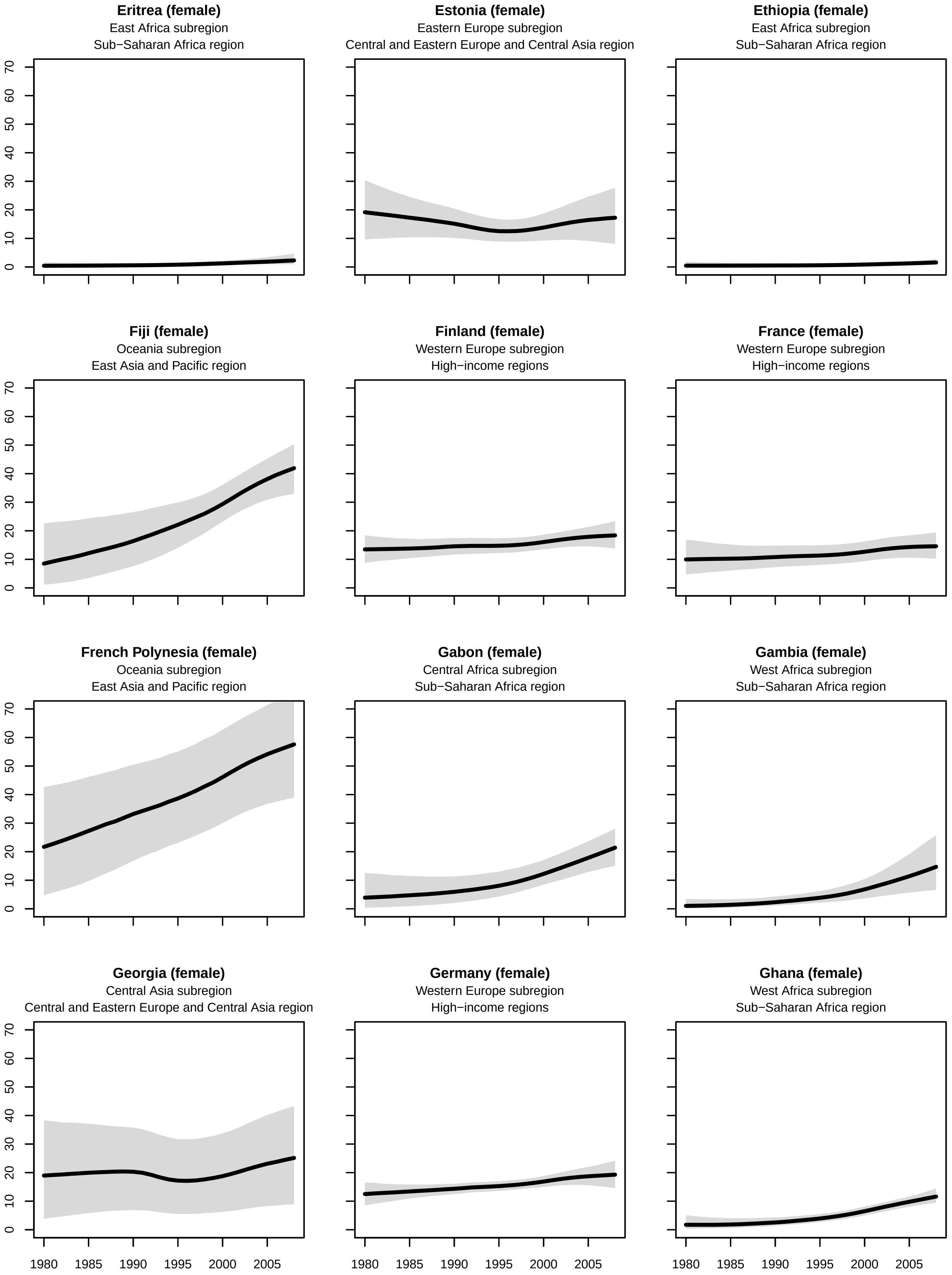
Eritrea (female)
East Africa subregion
Sub−Saharan Africa region
Estonia (female)
Eastern Europe subregion
Central and Eastern Europe and Central Asia region
Ethiopia (female)
East Africa subregion
Sub−Saharan Africa region
Fiji (female)
Oceania subregion
East Asia and Pacific region
Finland (female)
Western Europe subregion
High−income regions
France (female)
Western Europe subregion
High−income regions
French Polynesia (female)
Oceania subregion
East Asia and Pacific region
Gabon (female)
Central Africa subregion
Sub−Saharan Africa region
Gambia (female)
West Africa subregion
Sub−Saharan Africa region
Georgia (female)
Central Asia subregion
Central and Eastern Europe and Central Asia region
Germany (female)
Western Europe subregion
High−income regions
Ghana (female)
West Africa subregion
Sub−Saharan Africa region
0 10 20 30 40 50 60 70
1980 1985 1990 1995 2000 2005

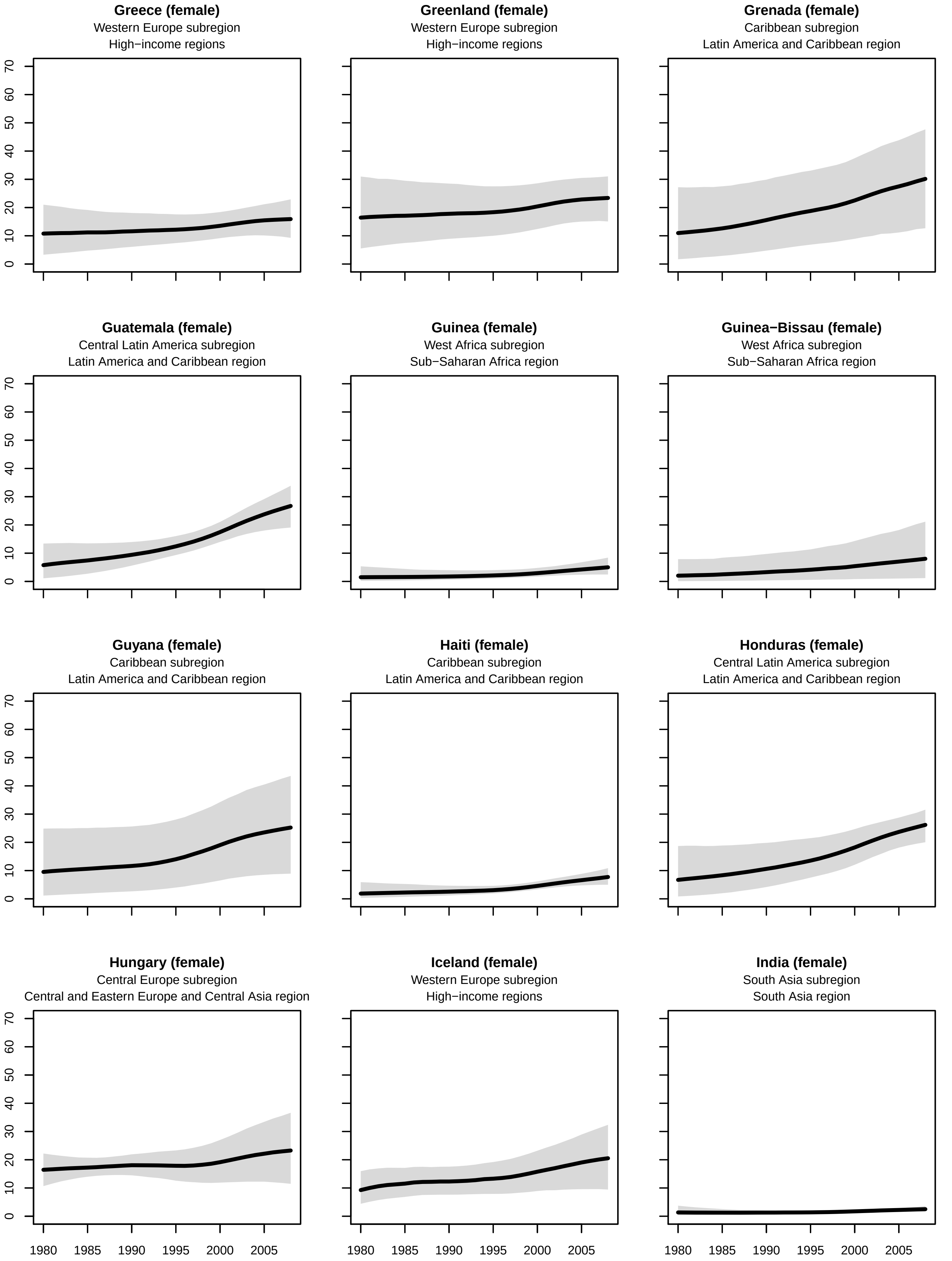
Greece (female)
Western Europe subregion
High−income regions
Greenland (female)
Western Europe subregion
High−income regions
Grenada (female)
Caribbean subregion
Latin America and Caribbean region
Guatemala (female)
Central Latin America subregion
Latin America and Caribbean region
Guinea (female)
West Africa subregion
Sub−Saharan Africa region
Guinea−Bissau (female)
West Africa subregion
Sub−Saharan Africa region
Guyana (female)
Caribbean subregion
Latin America and Caribbean region
Haiti (female)
Caribbean subregion
Latin America and Caribbean region
Honduras (female)
Central Latin America subregion
Latin America and Caribbean region
Hungary (female)
Central Europe subregion
Central and Eastern Europe and Central Asia region
Iceland (female)
Western Europe subregion
High−income regions
India (female)
South Asia subregion
South Asia region
0
10
20
30
40
50
60
70
1980
1985
1990
1995
2000
2005

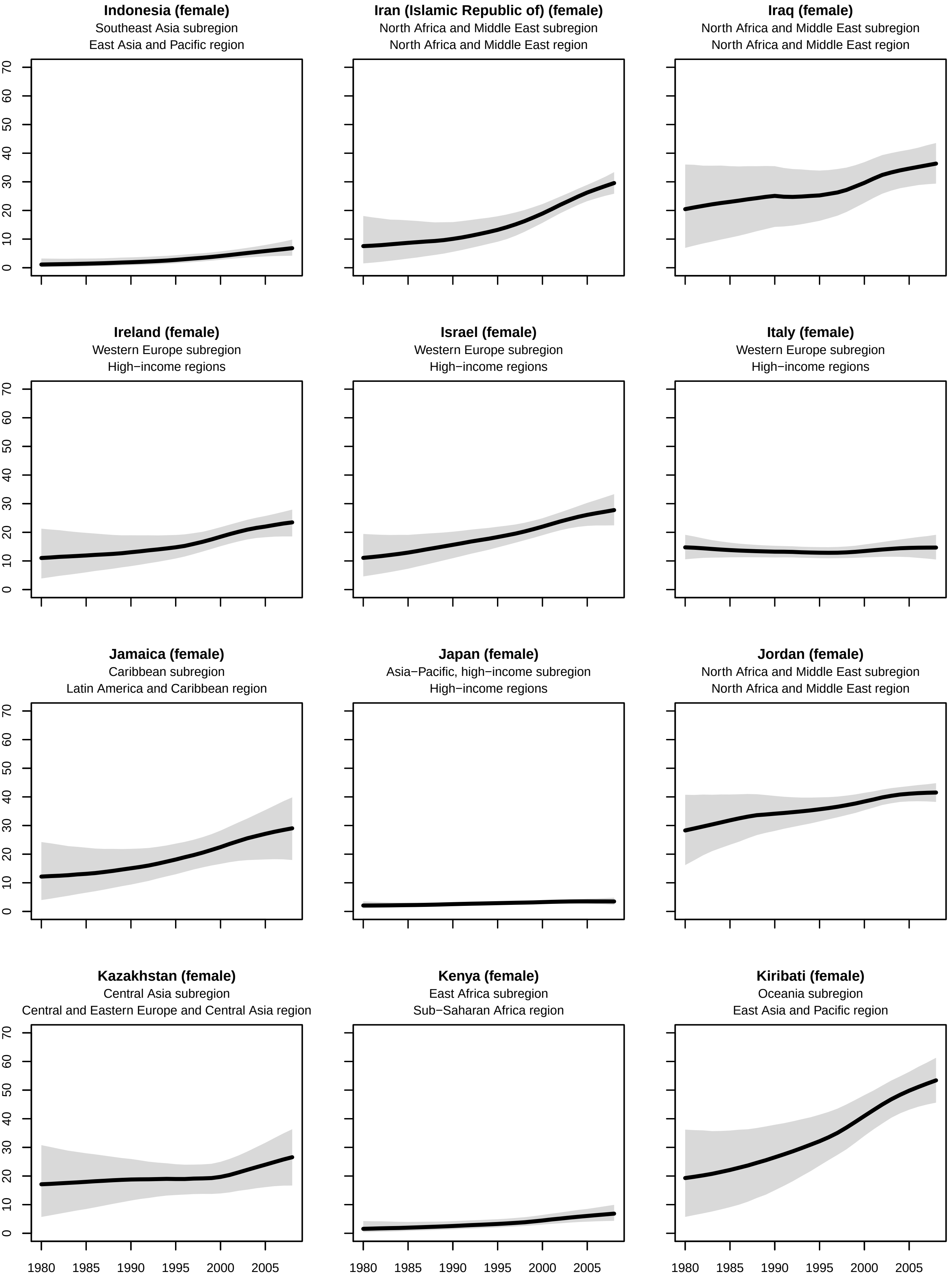
Indonesia (female)
Southeast Asia subregion
East Asia and Pacific region
Iran (Islamic Republic of) (female)
North Africa and Middle East subregion
North Africa and Middle East region
Iraq (female)
North Africa and Middle East subregion
North Africa and Middle East region
Ireland (female)
Western Europe subregion
High−income regions
Israel (female)
Western Europe subregion
High−income regions
Italy (female)
Western Europe subregion
High−income regions
Jamaica (female)
Caribbean subregion
Latin America and Caribbean region
Japan (female)
Asia−Pacific, high−income subregion
High−income regions
Jordan (female)
North Africa and Middle East subregion
North Africa and Middle East region
Kazakhstan (female)
Central Asia subregion
Central and Eastern Europe and Central Asia region
Kenya (female)
East Africa subregion
Sub−Saharan Africa region
Kiribati (female)
Oceania subregion
East Asia and Pacific region
0
10
20
30
40
50
60
70
1980
1985
1990
1995
2000
2005

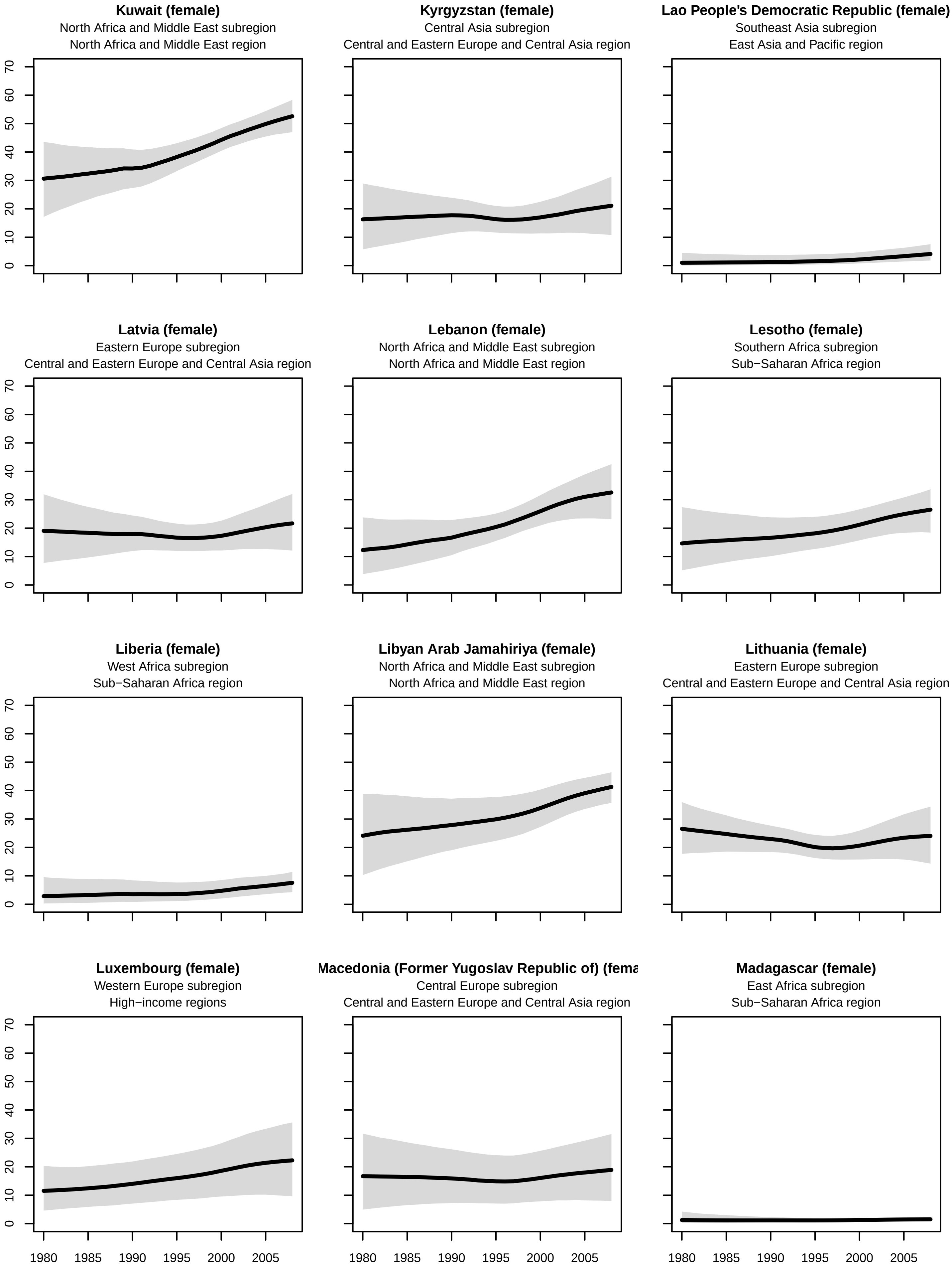
Kuwait (female)
North Africa and Middle East subregion
North Africa and Middle East region
Kyrgyzstan (female)
Central Asia subregion
Central and Eastern Europe and Central Asia region
Lao People's Democratic Republic (female)
Southeast Asia subregion
East Asia and Pacific region
Latvia (female)
Eastern Europe subregion
Central and Eastern Europe and Central Asia region
Lebanon (female)
North Africa and Middle East subregion
North Africa and Middle East region
Lesotho (female)
Southern Africa subregion
Sub−Saharan Africa region
Liberia (female)
West Africa subregion
Sub−Saharan Africa region
Libyan Arab Jamahiriya (female)
North Africa and Middle East subregion
North Africa and Middle East region
Lithuania (female)
Eastern Europe subregion
Central and Eastern Europe and Central Asia region
Luxembourg (female)
Western Europe subregion
High−income regions
Macedonia (Former Yugoslav Republic of) (fema
Central Europe subregion
Central and Eastern Europe and Central Asia region
Madagascar (female)
East Africa subregion
Sub−Saharan Africa region
0
10
20
30
40
50
60
70
1980
1985
1990
1995
2000
2005

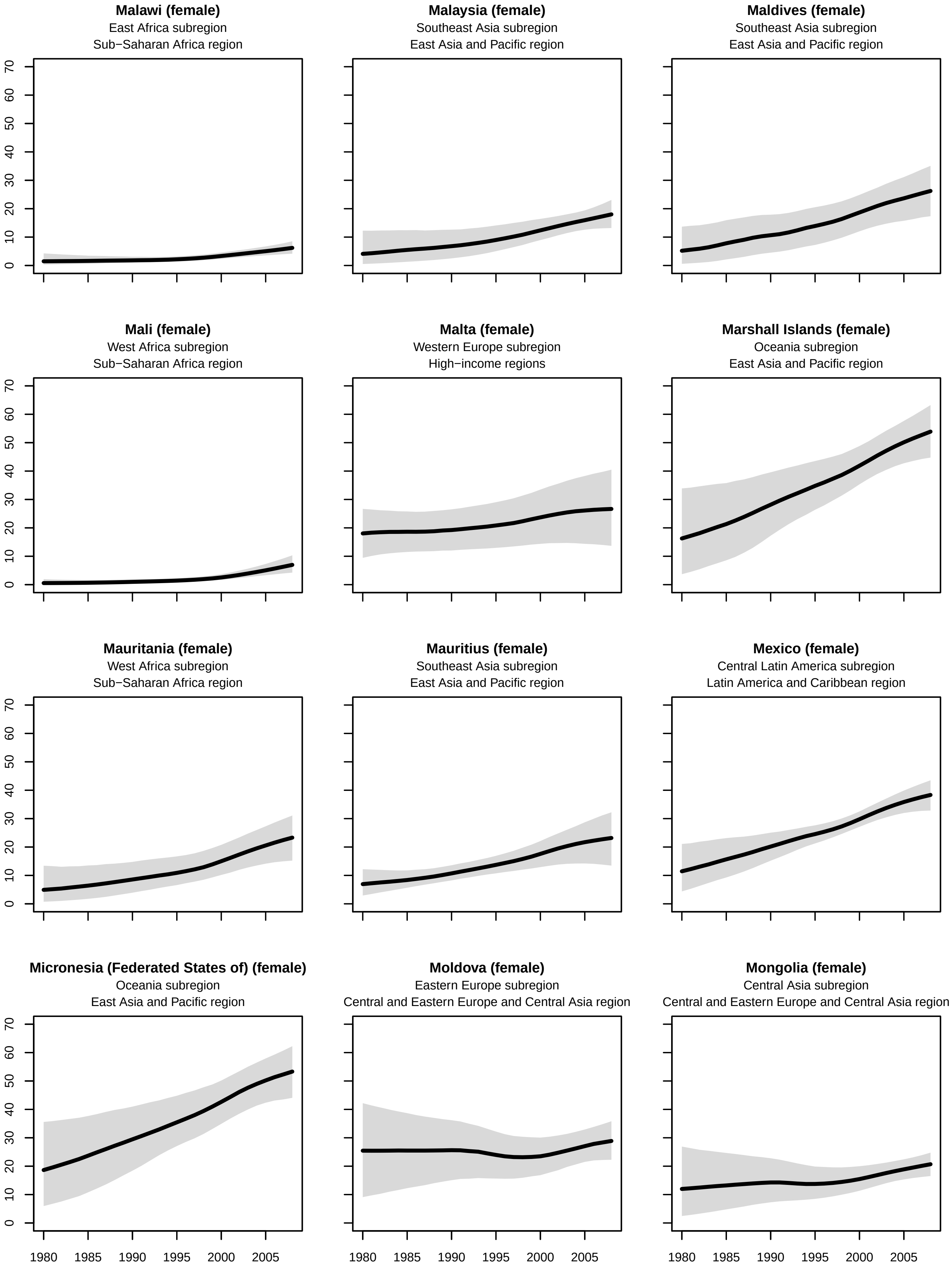
Malawi (female)
East Africa subregion
Sub−Saharan Africa region
Malaysia (female)
Southeast Asia subregion
East Asia and Pacific region
Maldives (female)
Southeast Asia subregion
East Asia and Pacific region
Mali (female)
West Africa subregion
Sub−Saharan Africa region
Malta (female)
Western Europe subregion
High−income regions
Marshall Islands (female)
Oceania subregion
East Asia and Pacific region
Mauritania (female)
West Africa subregion
Sub−Saharan Africa region
Mauritius (female)
Southeast Asia subregion
East Asia and Pacific region
Mexico (female)
Central Latin America subregion
Latin America and Caribbean region
Micronesia (Federated States of) (female)
Oceania subregion
East Asia and Pacific region
Moldova (female)
Eastern Europe subregion
Central and Eastern Europe and Central Asia region
Mongolia (female)
Central Asia subregion
Central and Eastern Europe and Central Asia region
0
10
20
30
40
50
60
70
1980
1985
1990
1995
2000
2005

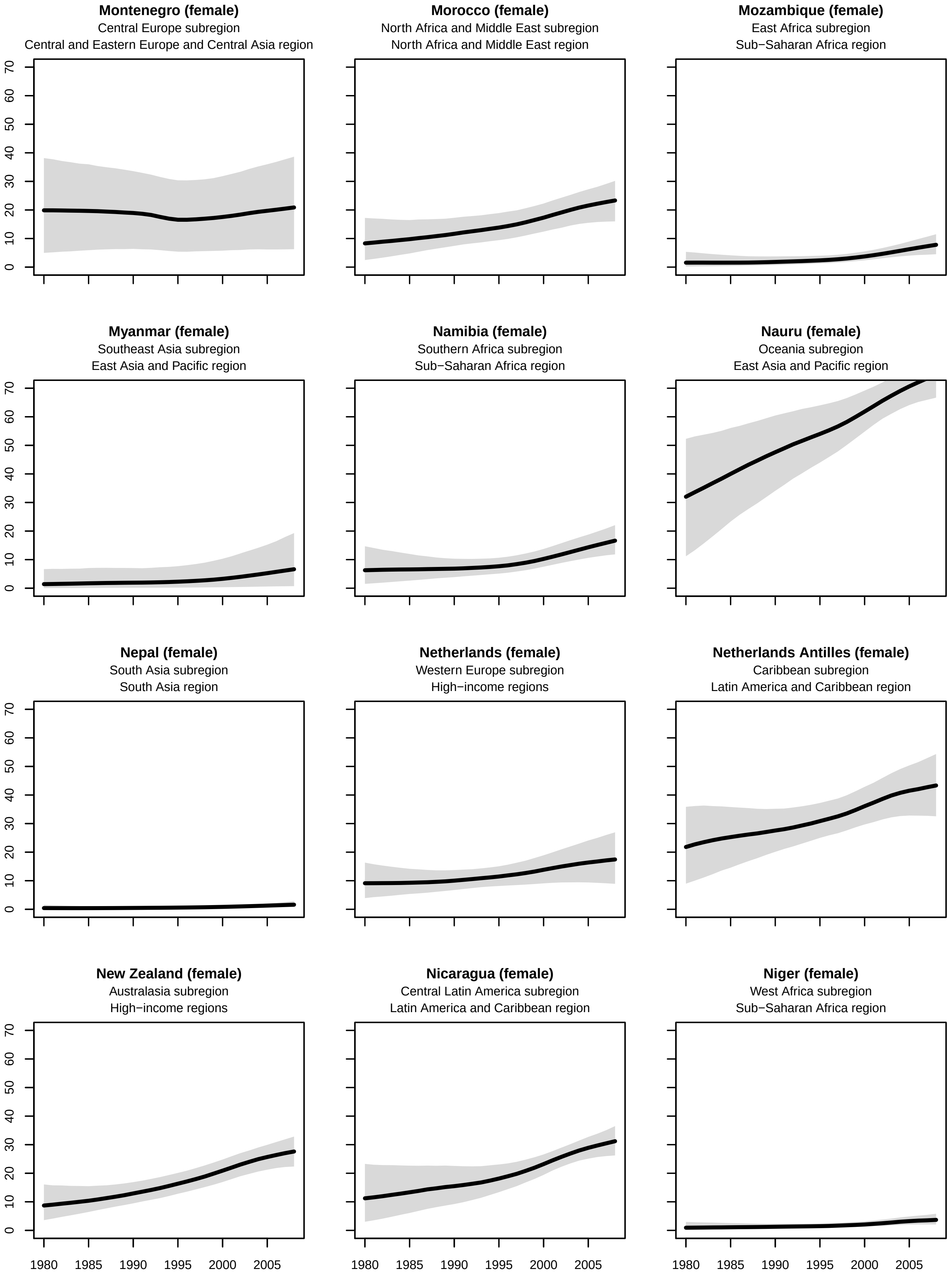
Montenegro (female)
Central Europe subregion
Central and Eastern Europe and Central Asia region
Morocco (female)
North Africa and Middle East subregion
North Africa and Middle East region
Mozambique (female)
East Africa subregion
Sub−Saharan Africa region
Myanmar (female)
Southeast Asia subregion
East Asia and Pacific region
Namibia (female)
Southern Africa subregion
Sub−Saharan Africa region
Nauru (female)
Oceania subregion
East Asia and Pacific region
Nepal (female)
South Asia subregion
South Asia region
Netherlands (female)
Western Europe subregion
High−income regions
Netherlands Antilles (female)
Caribbean subregion
Latin America and Caribbean region
New Zealand (female)
Australasia subregion
High−income regions
Nicaragua (female)
Central Latin America subregion
Latin America and Caribbean region
Niger (female)
West Africa subregion
Sub−Saharan Africa region
0 10 20 30 40 50 60 70
1980 1985 1990 1995 2000 2005

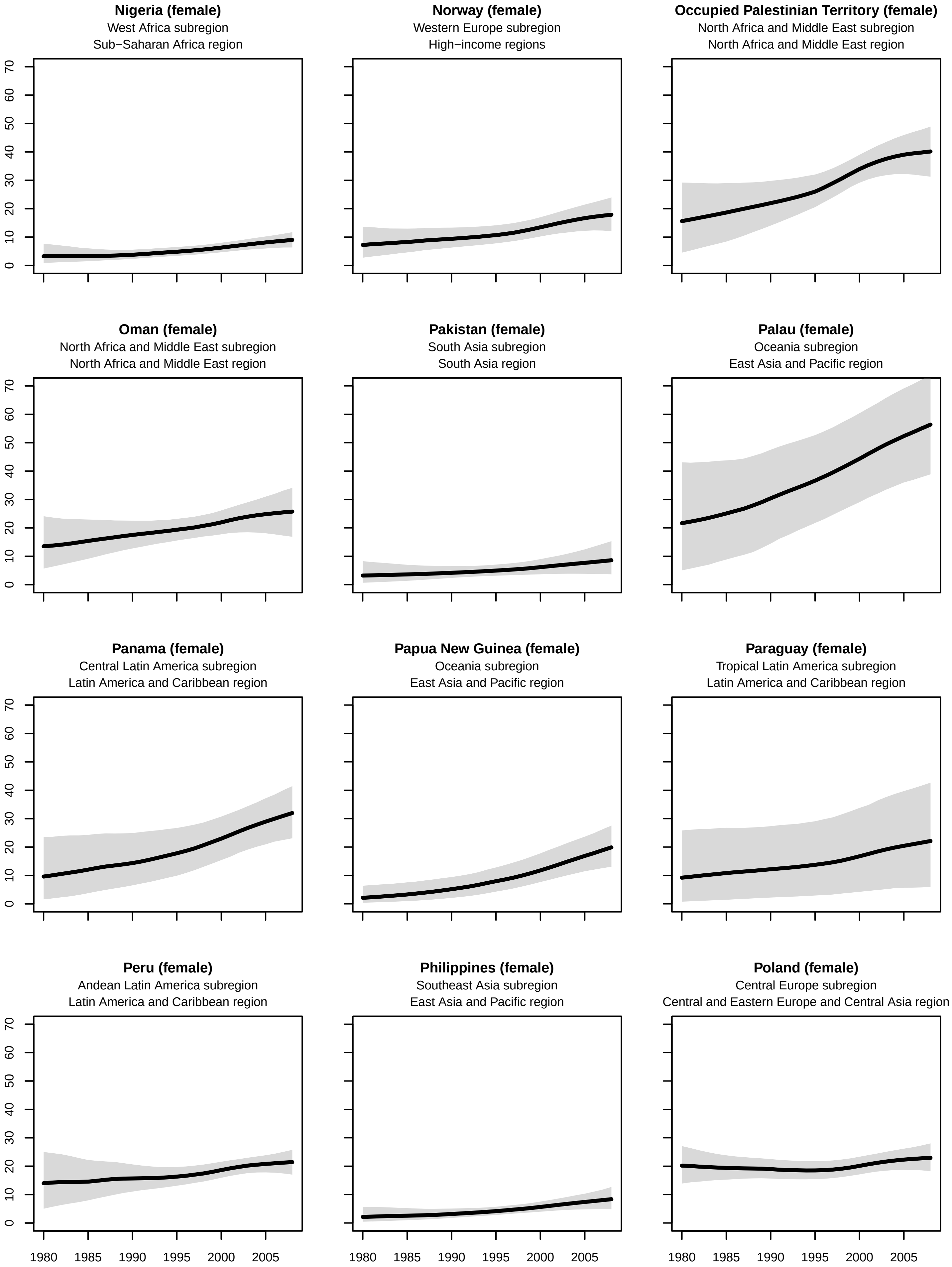
Nigeria (female)
West Africa subregion
Sub−Saharan Africa region
Norway (female)
Western Europe subregion
High−income regions
Occupied Palestinian Territory (female)
North Africa and Middle East subregion
North Africa and Middle East region
Oman (female)
North Africa and Middle East subregion
North Africa and Middle East region
Pakistan (female)
South Asia subregion
South Asia region
Palau (female)
Oceania subregion
East Asia and Pacific region
Panama (female)
Central Latin America subregion
Latin America and Caribbean region
Papua New Guinea (female)
Oceania subregion
East Asia and Pacific region
Paraguay (female)
Tropical Latin America subregion
Latin America and Caribbean region
Peru (female)
Andean Latin America subregion
Latin America and Caribbean region
Philippines (female)
Southeast Asia subregion
East Asia and Pacific region
Poland (female)
Central Europe subregion
Central and Eastern Europe and Central Asia region
0
10
20
30
40
50
60
70
1980
1985
1990
1995
2000
2005

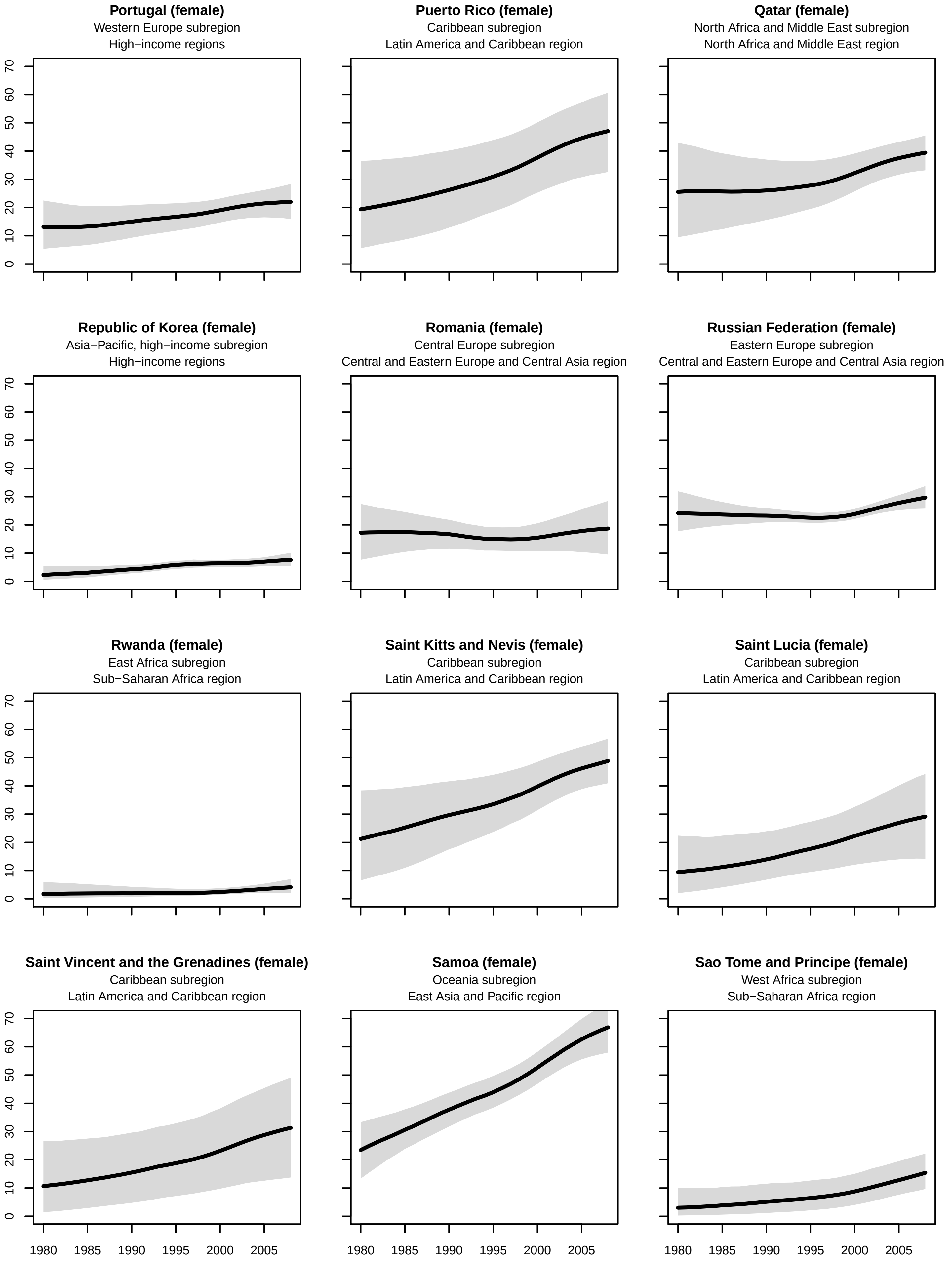
Portugal (female)
Western Europe subregion
High−income regions
Puerto Rico (female)
Caribbean subregion
Latin America and Caribbean region
Qatar (female)
North Africa and Middle East subregion
North Africa and Middle East region
Republic of Korea (female)
Asia−Pacific, high−income subregion
High−income regions
Romania (female)
Central Europe subregion
Central and Eastern Europe and Central Asia region
Russian Federation (female)
Eastern Europe subregion
Central and Eastern Europe and Central Asia region
Rwanda (female)
East Africa subregion
Sub−Saharan Africa region
Saint Kitts and Nevis (female)
Caribbean subregion
Latin America and Caribbean region
Saint Lucia (female)
Caribbean subregion
Latin America and Caribbean region
Saint Vincent and the Grenadines (female)
Caribbean subregion
Latin America and Caribbean region
Samoa (female)
Oceania subregion
East Asia and Pacific region
Sao Tome and Principe (female)
West Africa subregion
Sub−Saharan Africa region
0 10 20 30 40 50 60 70
1980 1985 1990 1995 2000 2005

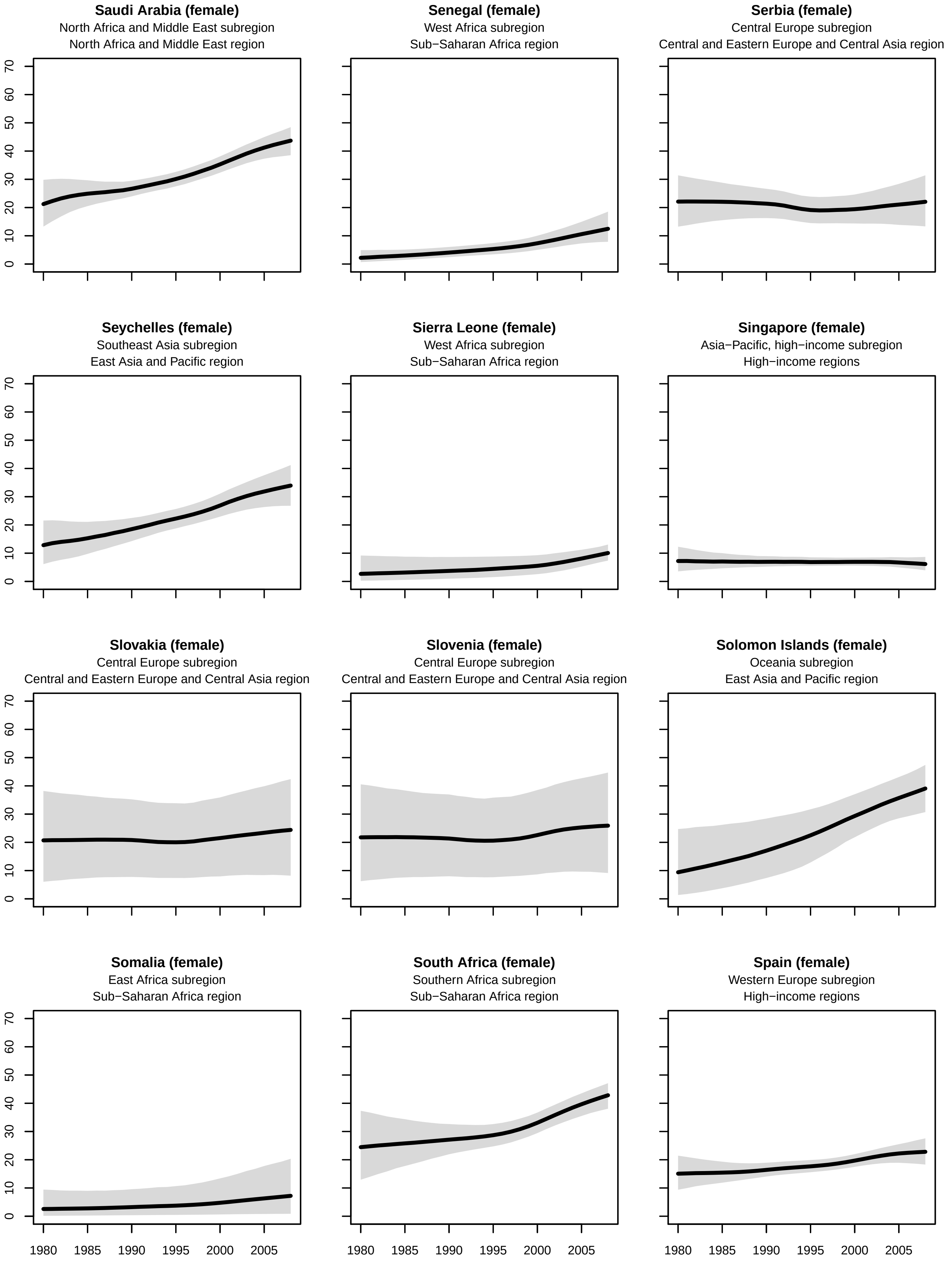
Saudi Arabia (female)
North Africa and Middle East subregion
North Africa and Middle East region
Senegal (female)
West Africa subregion
Sub−Saharan Africa region
Serbia (female)
Central Europe subregion
Central and Eastern Europe and Central Asia region
Seychelles (female)
Southeast Asia subregion
East Asia and Pacific region
Sierra Leone (female)
West Africa subregion
Sub−Saharan Africa region
Singapore (female)
Asia−Pacific, high−income subregion
High−income regions
Slovakia (female)
Central Europe subregion
Central and Eastern Europe and Central Asia region
Slovenia (female)
Central Europe subregion
Central and Eastern Europe and Central Asia region
Solomon Islands (female)
Oceania subregion
East Asia and Pacific region
Somalia (female)
East Africa subregion
Sub−Saharan Africa region
South Africa (female)
Southern Africa subregion
Sub−Saharan Africa region
Spain (female)
Western Europe subregion
High−income regions
0
10
20
30
40
50
60
70
1980
1985
1990
1995
2000
2005

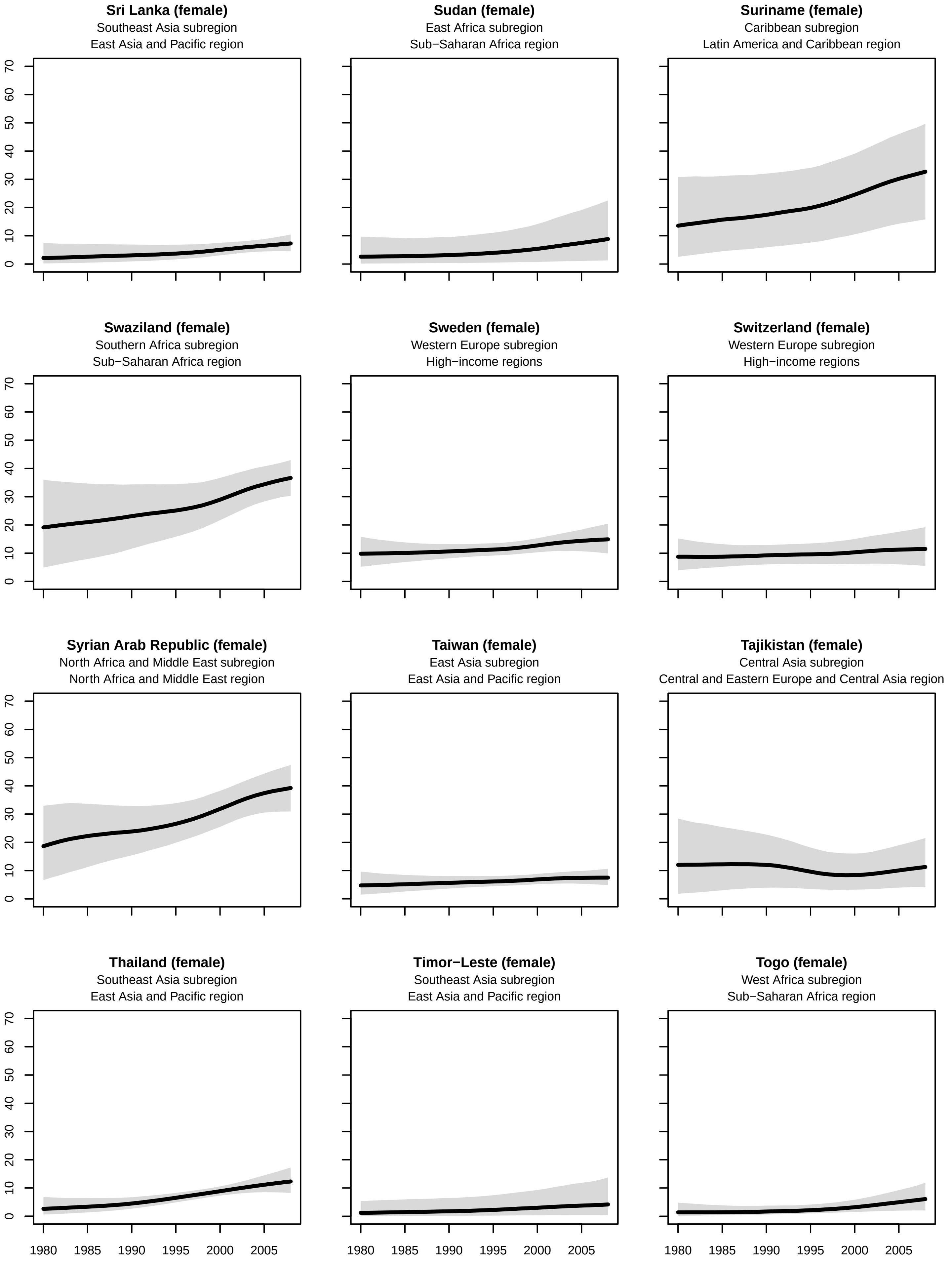

Sri Lanka (female)
Southeast Asia subregion
East Asia and Pacific region
Sudan (female)
East Africa subregion
Sub−Saharan Africa region
Suriname (female)
Caribbean subregion
Latin America and Caribbean region
Swaziland (female)
Southern Africa subregion
Sub−Saharan Africa region
Sweden (female)
Western Europe subregion
High−income regions
Switzerland (female)
Western Europe subregion
High−income regions
Syrian Arab Republic (female)
North Africa and Middle East subregion
North Africa and Middle East region
Taiwan (female)
East Asia subregion
East Asia and Pacific region
Tajikistan (female)
Central Asia subregion
Central and Eastern Europe and Central Asia region
Thailand (female)
Southeast Asia subregion
East Asia and Pacific region
Timor−Leste (female)
Southeast Asia subregion
East Asia and Pacific region
Togo (female)
West Africa subregion
Sub−Saharan Africa region
0
10
20
30
40
50
60
70
1980
1985
1990
1995
2000
2005

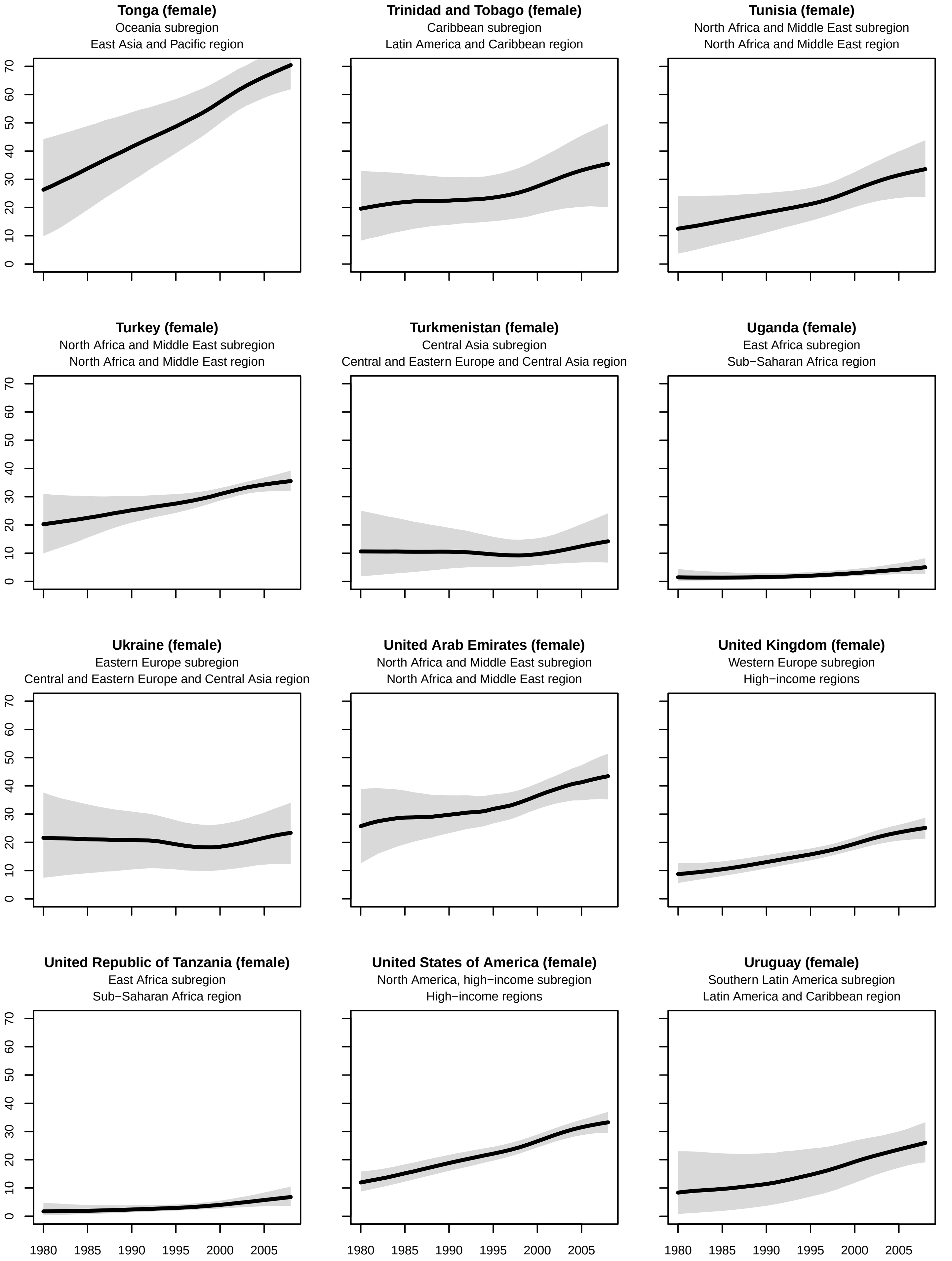
Tonga (female)
Oceania subregion
East Asia and Pacific region
Trinidad and Tobago (female)
Caribbean subregion
Latin America and Caribbean region
Tunisia (female)
North Africa and Middle East subregion
North Africa and Middle East region
Turkey (female)
North Africa and Middle East subregion
North Africa and Middle East region
Turkmenistan (female)
Central Asia subregion
Central and Eastern Europe and Central Asia region
Uganda (female)
East Africa subregion
Sub−Saharan Africa region
Ukraine (female)
Eastern Europe subregion
Central and Eastern Europe and Central Asia region
United Arab Emirates (female)
North Africa and Middle East subregion
North Africa and Middle East region
United Kingdom (female)
Western Europe subregion
High−income regions
United Republic of Tanzania (female)
East Africa subregion
Sub−Saharan Africa region
United States of America (female)
North America, high−income subregion
High−income regions
Uruguay (female)
Southern Latin America subregion
Latin America and Caribbean region
0 10 20 30 40 50 60 70
1980 1985 1990 1995 2000 2005

Uzbekistan (female)
Central Asia subregion
Central and Eastern Europe and Central Asia region

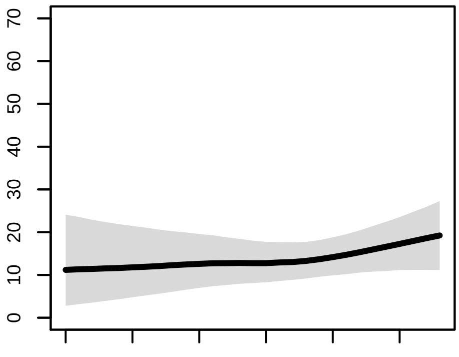

Vanuatu (female)
Oceania subregion
East Asia and Pacific region

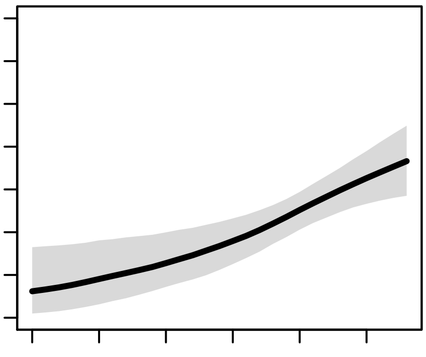

Venezuela (Bolivarian Republic of) (female)
Central Latin America subregion
Latin America and Caribbean region

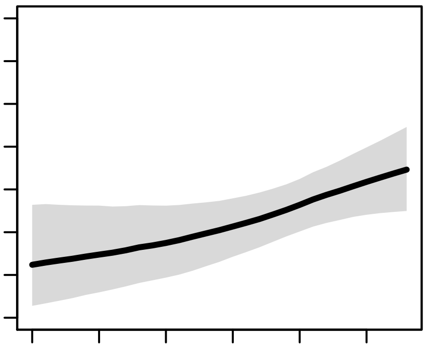

Viet Nam (female)
Southeast Asia subregion
East Asia and Pacific region

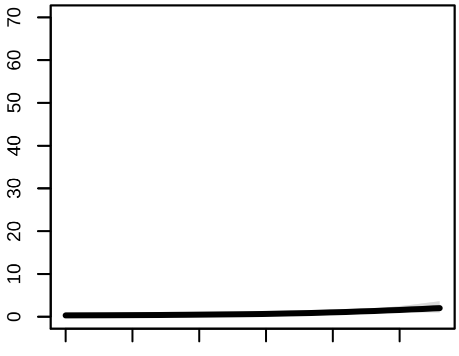

Yemen (female)
North Africa and Middle East subregion
North Africa and Middle East region

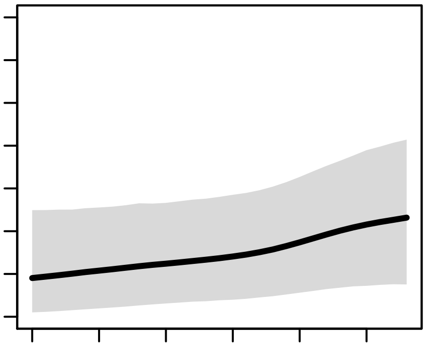

Zambia (female)
East Africa subregion
Sub−Saharan Africa region

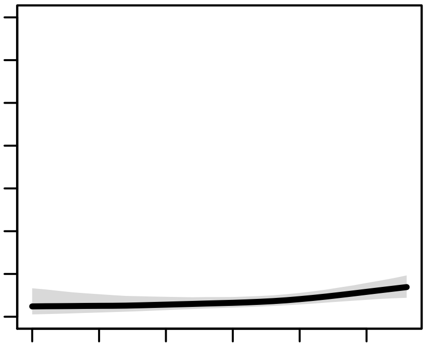

Zimbabwe (female)
Southern Africa subregion
Sub−Saharan Africa region

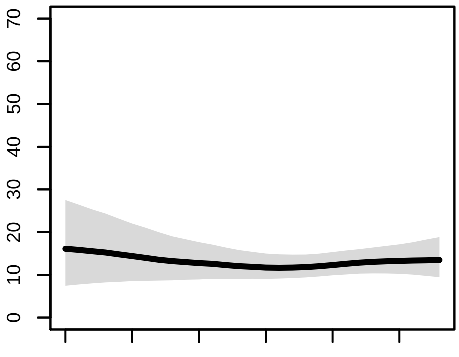

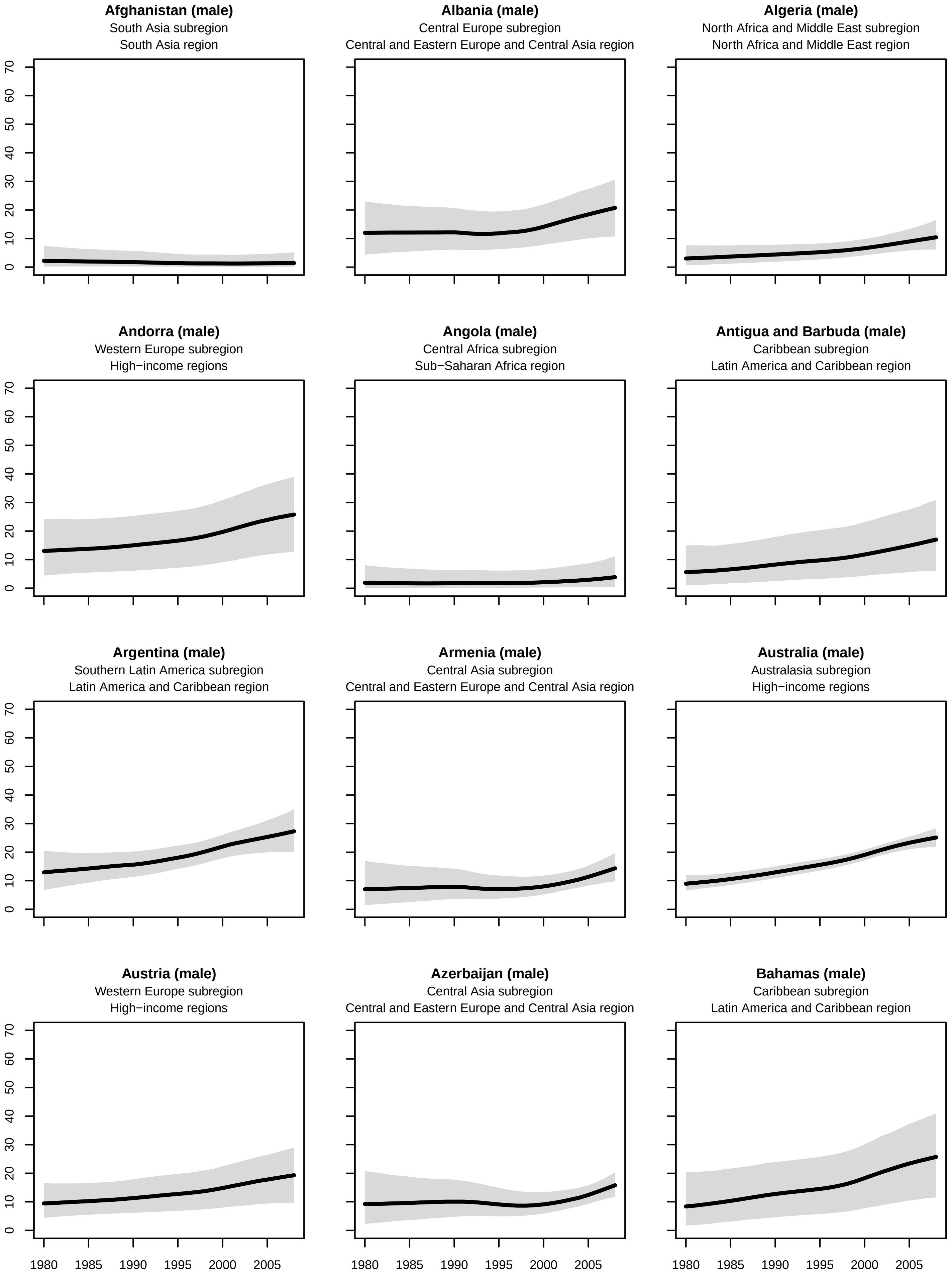
Afghanistan (male)
South Asia subregion
South Asia region
Albania (male)
Central Europe subregion
Central and Eastern Europe and Central Asia region
Algeria (male)
North Africa and Middle East subregion
North Africa and Middle East region
Andorra (male)
Western Europe subregion
High−income regions
Angola (male)
Central Africa subregion
Sub−Saharan Africa region
Antigua and Barbuda (male)
Caribbean subregion
Latin America and Caribbean region
Argentina (male)
Southern Latin America subregion
Latin America and Caribbean region
Armenia (male)
Central Asia subregion
Central and Eastern Europe and Central Asia region
Australia (male)
Australasia subregion
High−income regions
Austria (male)
Western Europe subregion
High−income regions
Azerbaijan (male)
Central Asia subregion
Central and Eastern Europe and Central Asia region
Bahamas (male)
Caribbean subregion
Latin America and Caribbean region
0 10 20 30 40 50 60 70
1980 1985 1990 1995 2000 2005

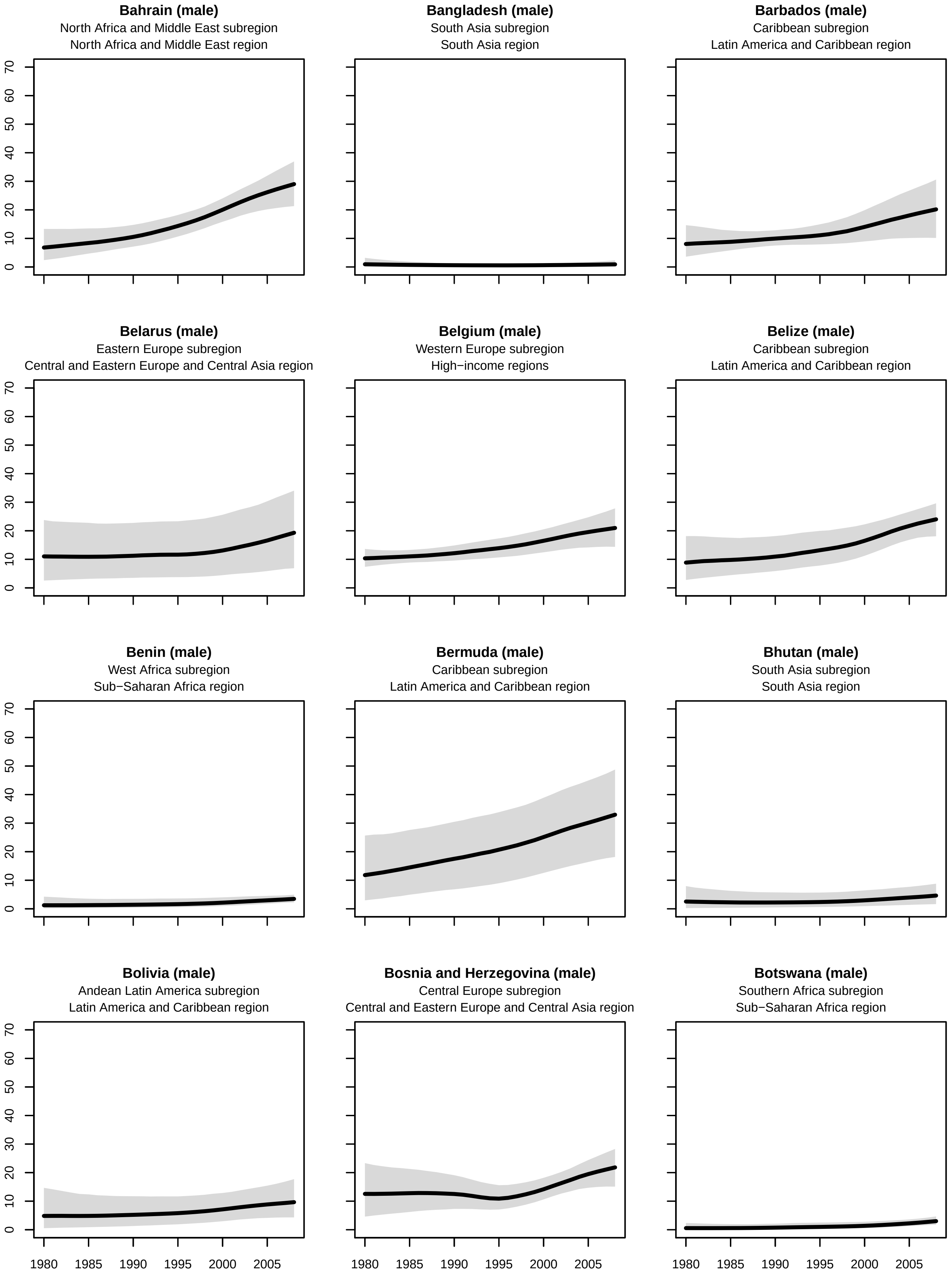

Bahrain (male)
North Africa and Middle East subregion
North Africa and Middle East region
Bangladesh (male)
South Asia subregion
South Asia region
Barbados (male)
Caribbean subregion
Latin America and Caribbean region
Belarus (male)
Eastern Europe subregion
Central and Eastern Europe and Central Asia region
Belgium (male)
Western Europe subregion
High−income regions
Belize (male)
Caribbean subregion
Latin America and Caribbean region
Benin (male)
West Africa subregion
Sub−Saharan Africa region
Bermuda (male)
Caribbean subregion
Latin America and Caribbean region
Bhutan (male)
South Asia subregion
South Asia region
Bolivia (male)
Andean Latin America subregion
Latin America and Caribbean region
Bosnia and Herzegovina (male)
Central Europe subregion
Central and Eastern Europe and Central Asia region
Botswana (male)
Southern Africa subregion
Sub−Saharan Africa region
0
10
20
30
40
50
60
70
1980
1985
1990
1995
2000
2005

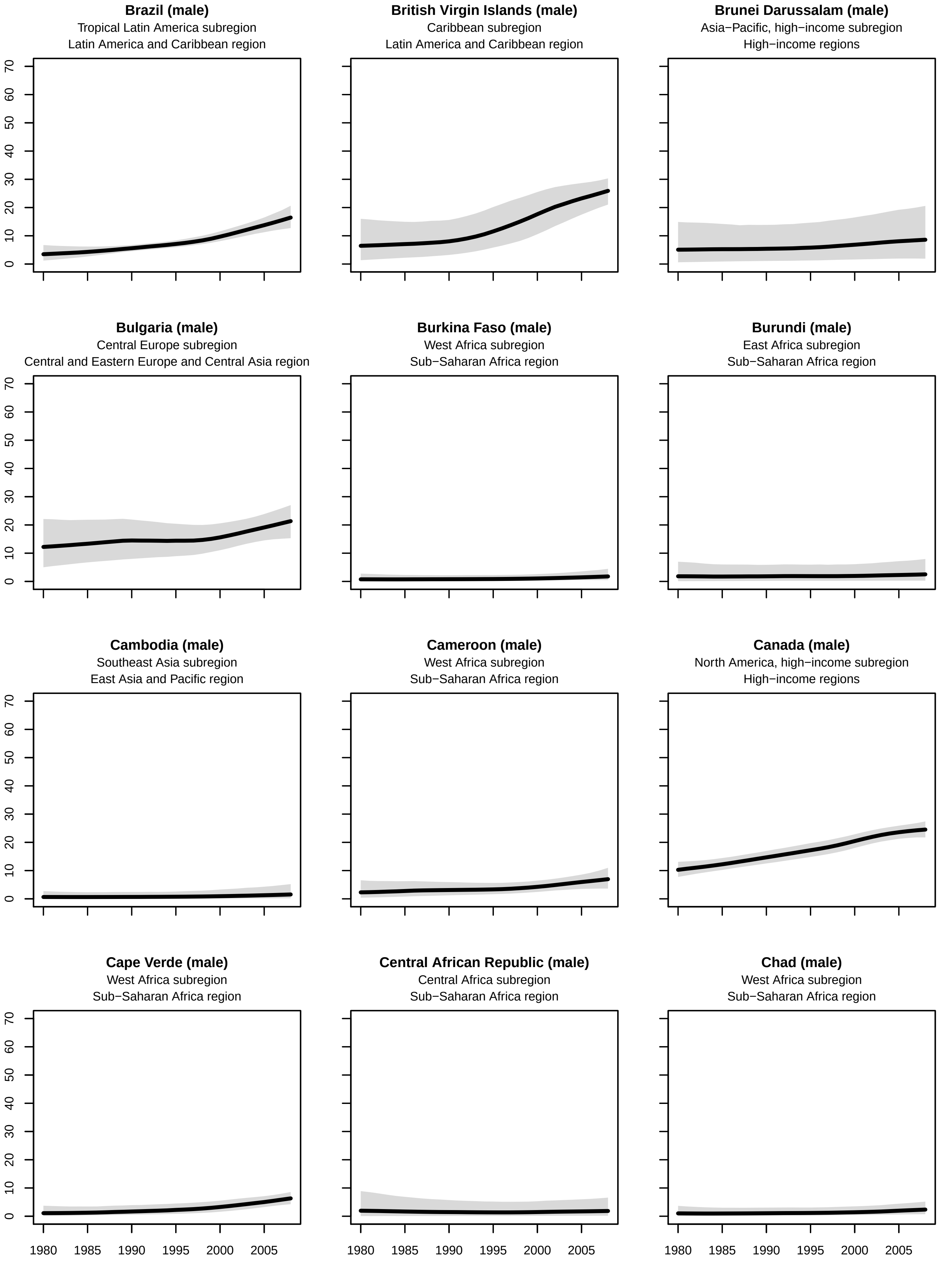

Brazil (male)
Tropical Latin America subregion
Latin America and Caribbean region
British Virgin Islands (male)
Caribbean subregion
Latin America and Caribbean region
Brunei Darussalam (male)
Asia−Pacific, high−income subregion
High−income regions
Bulgaria (male)
Central Europe subregion
Central and Eastern Europe and Central Asia region
Burkina Faso (male)
West Africa subregion
Sub−Saharan Africa region
Burundi (male)
East Africa subregion
Sub−Saharan Africa region
Cambodia (male)
Southeast Asia subregion
East Asia and Pacific region
Cameroon (male)
West Africa subregion
Sub−Saharan Africa region
Canada (male)
North America, high−income subregion
High−income regions
Cape Verde (male)
West Africa subregion
Sub−Saharan Africa region
Central African Republic (male)
Central Africa subregion
Sub−Saharan Africa region
Chad (male)
West Africa subregion
Sub−Saharan Africa region
0 10 20 30 40 50 60 70
1980 1985 1990 1995 2000 2005

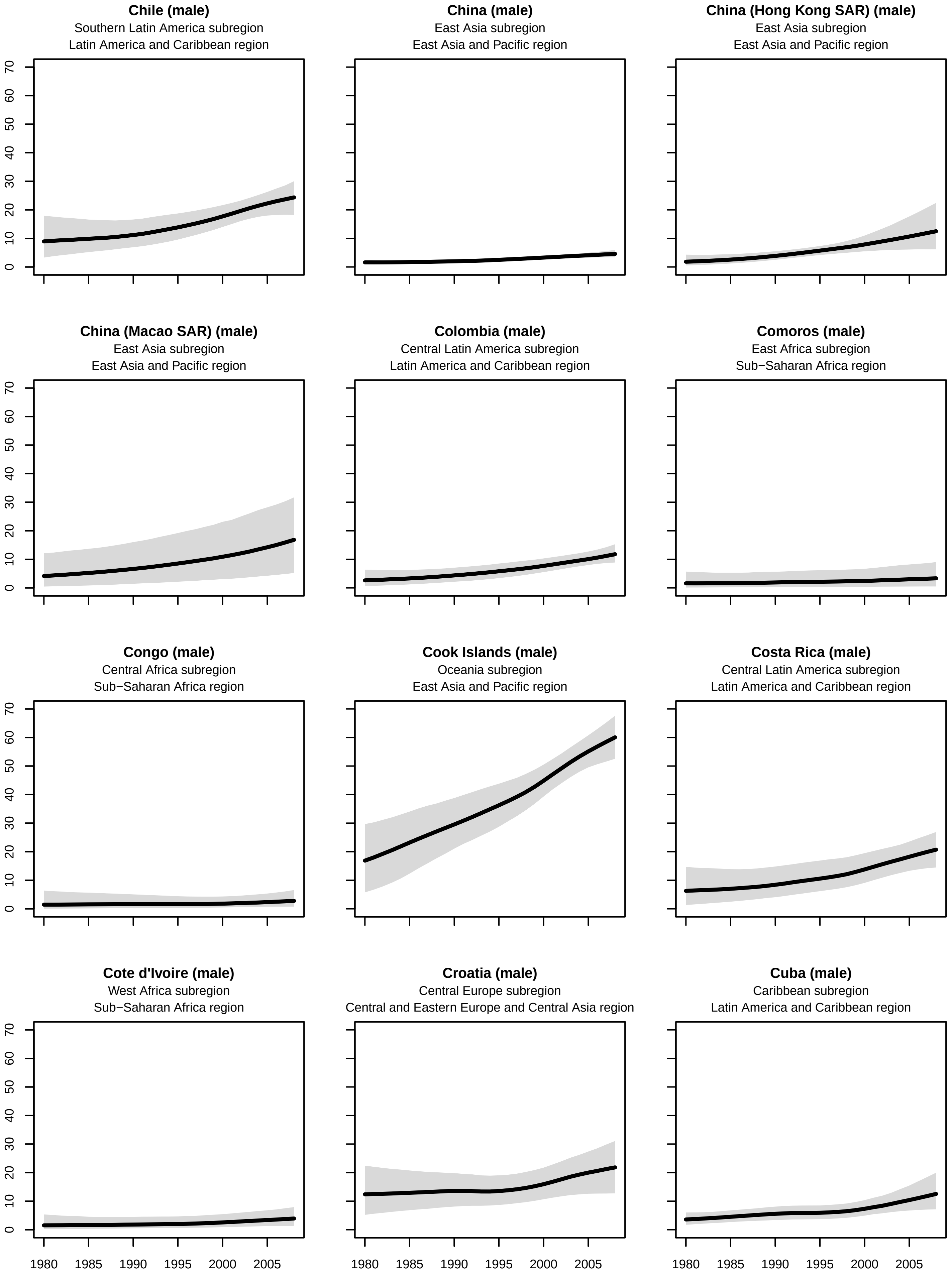
Chile (male)
Southern Latin America subregion
Latin America and Caribbean region
China (male)
East Asia subregion
East Asia and Pacific region
China (Hong Kong SAR) (male)
East Asia subregion
East Asia and Pacific region
China (Macao SAR) (male)
East Asia subregion
East Asia and Pacific region
Colombia (male)
Central Latin America subregion
Latin America and Caribbean region
Comoros (male)
East Africa subregion
Sub−Saharan Africa region
Congo (male)
Central Africa subregion
Sub−Saharan Africa region
Cook Islands (male)
Oceania subregion
East Asia and Pacific region
Costa Rica (male)
Central Latin America subregion
Latin America and Caribbean region
Cote d'Ivoire (male)
West Africa subregion
Sub−Saharan Africa region
Croatia (male)
Central Europe subregion
Central and Eastern Europe and Central Asia region
Cuba (male)
Caribbean subregion
Latin America and Caribbean region
0
10
20
30
40
50
60
70
1980
1985
1990
1995
2000
2005

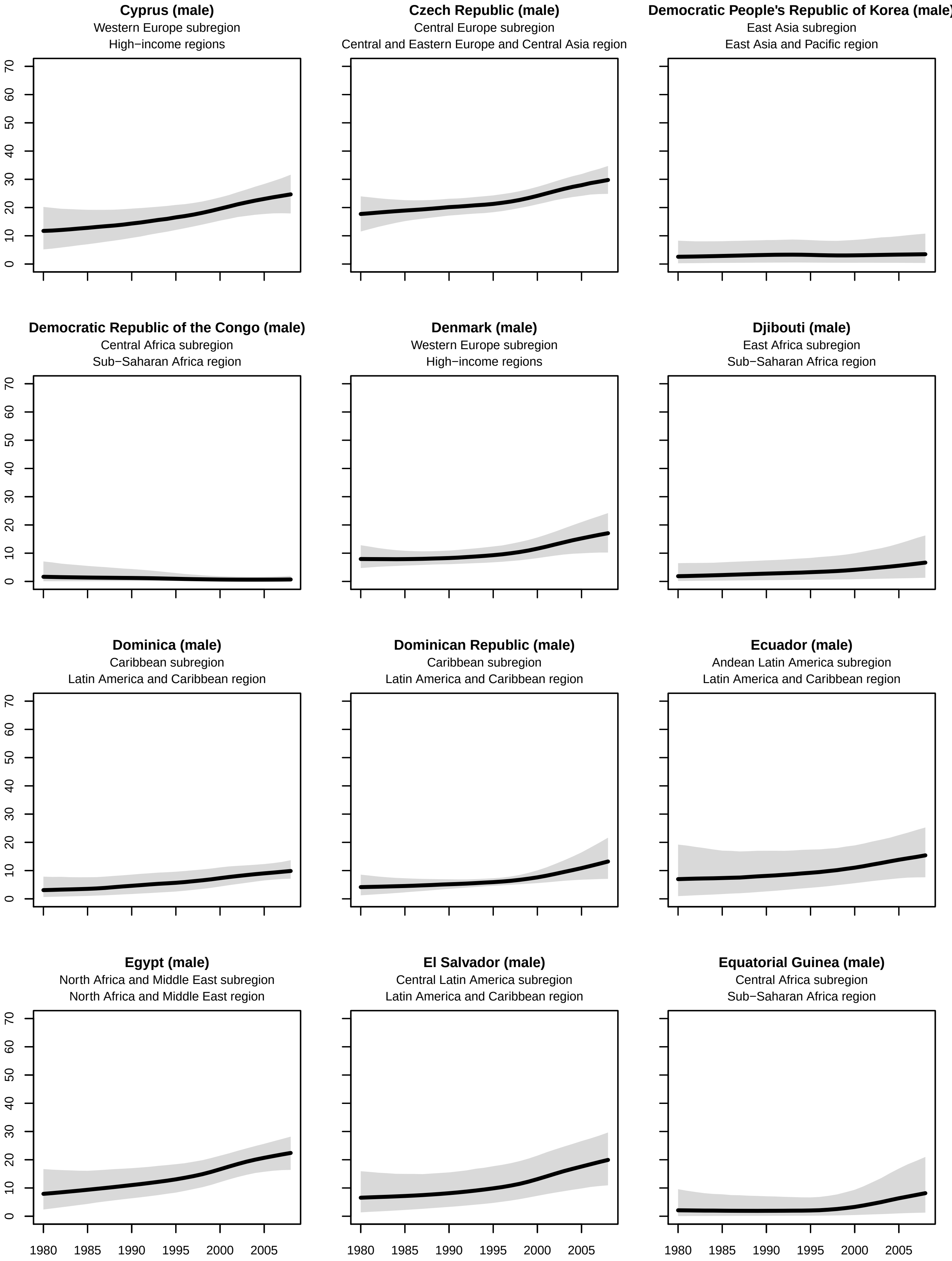
Cyprus (male)
Western Europe subregion
High−income regions
Czech Republic (male)
Central Europe subregion
Central and Eastern Europe and Central Asia region
Democratic People's Republic of Korea (male)
East Asia subregion
East Asia and Pacific region
Democratic Republic of the Congo (male)
Central Africa subregion
Sub−Saharan Africa region
Denmark (male)
Western Europe subregion
High−income regions
Djibouti (male)
East Africa subregion
Sub−Saharan Africa region
Dominica (male)
Caribbean subregion
Latin America and Caribbean region
Dominican Republic (male)
Caribbean subregion
Latin America and Caribbean region
Ecuador (male)
Andean Latin America subregion
Latin America and Caribbean region
Egypt (male)
North Africa and Middle East subregion
North Africa and Middle East region
El Salvador (male)
Central Latin America subregion
Latin America and Caribbean region
Equatorial Guinea (male)
Central Africa subregion
Sub−Saharan Africa region
0 10 20 30 40 50 60 70
1980 1985 1990 1995 2000 2005

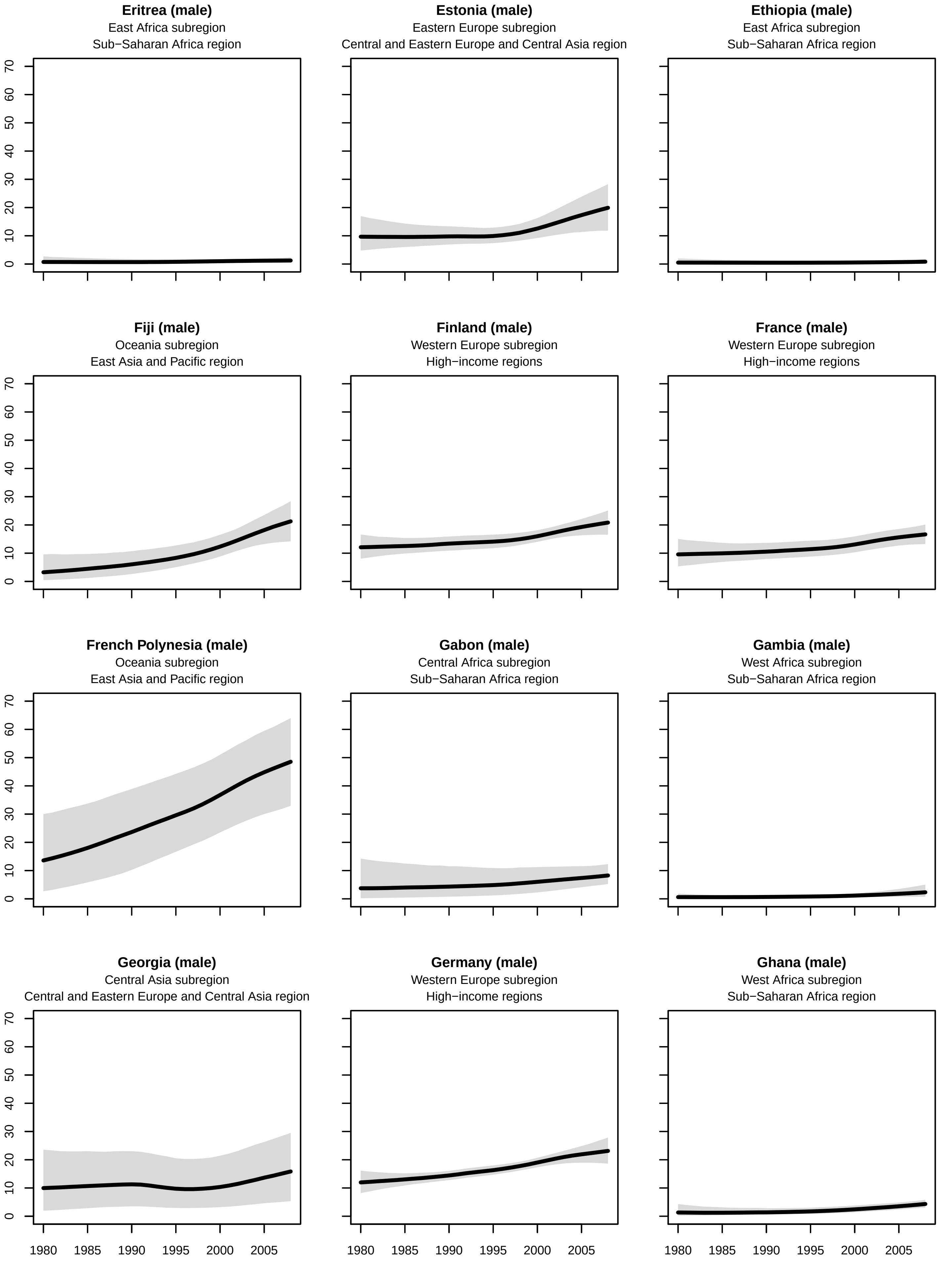
Eritrea (male)
East Africa subregion
Sub−Saharan Africa region
Estonia (male)
Eastern Europe subregion
Central and Eastern Europe and Central Asia region
Ethiopia (male)
East Africa subregion
Sub−Saharan Africa region
Fiji (male)
Oceania subregion
East Asia and Pacific region
Finland (male)
Western Europe subregion
High−income regions
France (male)
Western Europe subregion
High−income regions
French Polynesia (male)
Oceania subregion
East Asia and Pacific region
Gabon (male)
Central Africa subregion
Sub−Saharan Africa region
Gambia (male)
West Africa subregion
Sub−Saharan Africa region
Georgia (male)
Central Asia subregion
Central and Eastern Europe and Central Asia region
Germany (male)
Western Europe subregion
High−income regions
Ghana (male)
West Africa subregion
Sub−Saharan Africa region
0 10 20 30 40 50 60 70
1980 1985 1990 1995 2000 2005

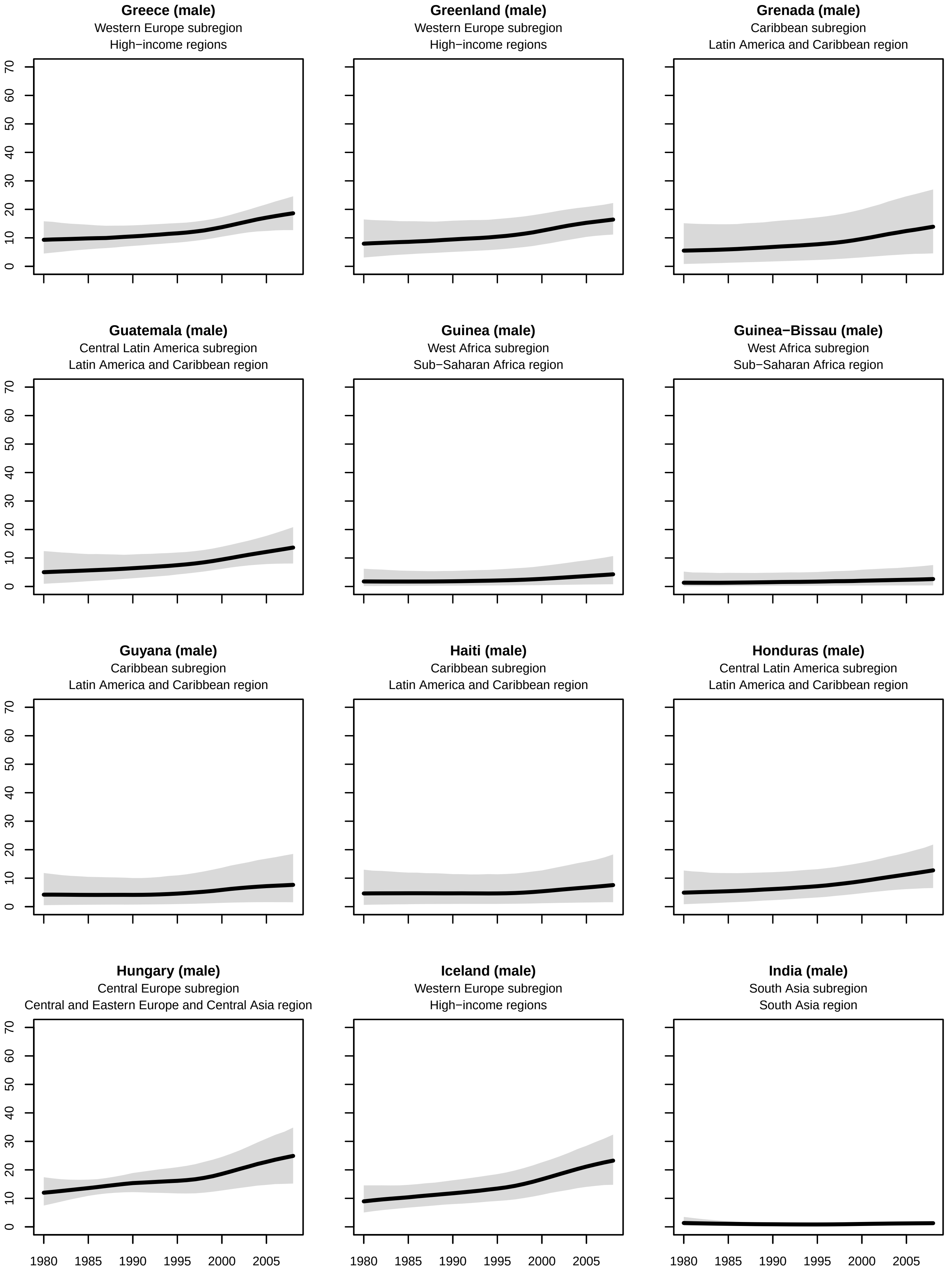
Greece (male)
Western Europe subregion
High−income regions
Greenland (male)
Western Europe subregion
High−income regions
Grenada (male)
Caribbean subregion
Latin America and Caribbean region
Guatemala (male)
Central Latin America subregion
Latin America and Caribbean region
Guinea (male)
West Africa subregion
Sub−Saharan Africa region
Guinea−Bissau (male)
West Africa subregion
Sub−Saharan Africa region
Guyana (male)
Caribbean subregion
Latin America and Caribbean region
Haiti (male)
Caribbean subregion
Latin America and Caribbean region
Honduras (male)
Central Latin America subregion
Latin America and Caribbean region
Hungary (male)
Central Europe subregion
Central and Eastern Europe and Central Asia region
Iceland (male)
Western Europe subregion
High−income regions
India (male)
South Asia subregion
South Asia region
0
10
20
30
40
50
60
70
1980
1985
1990
1995
2000
2005

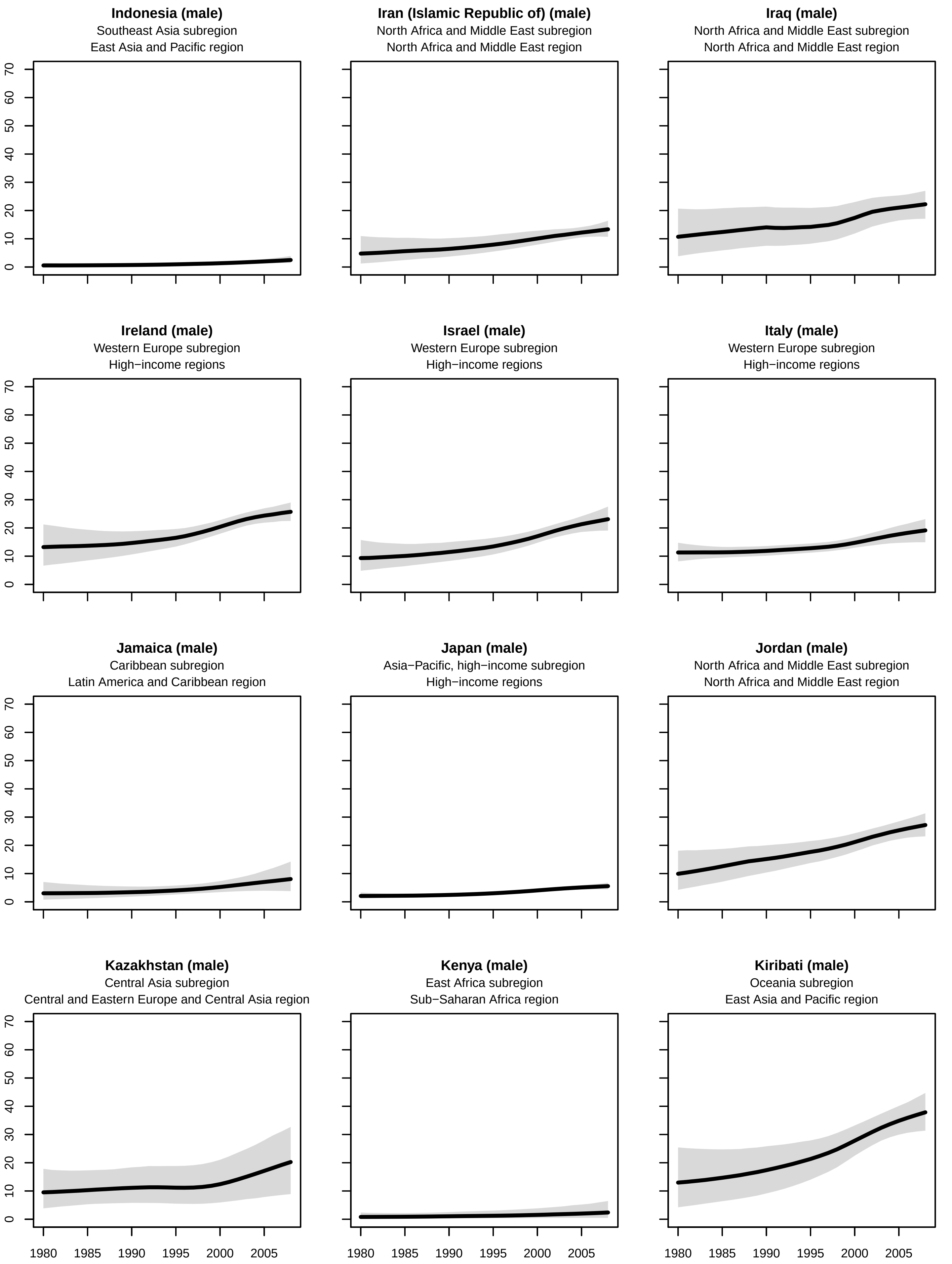
Indonesia (male)
Southeast Asia subregion
East Asia and Pacific region
Iran (Islamic Republic of) (male)
North Africa and Middle East subregion
North Africa and Middle East region
Iraq (male)
North Africa and Middle East subregion
North Africa and Middle East region
Ireland (male)
Western Europe subregion
High−income regions
Israel (male)
Western Europe subregion
High−income regions
Italy (male)
Western Europe subregion
High−income regions
Jamaica (male)
Caribbean subregion
Latin America and Caribbean region
Japan (male)
Asia−Pacific, high−income subregion
High−income regions
Jordan (male)
North Africa and Middle East subregion
North Africa and Middle East region
Kazakhstan (male)
Central Asia subregion
Central and Eastern Europe and Central Asia region
Kenya (male)
East Africa subregion
Sub−Saharan Africa region
Kiribati (male)
Oceania subregion
East Asia and Pacific region
0
10
20
30
40
50
60
70
1980
1985
1990
1995
2000
2005

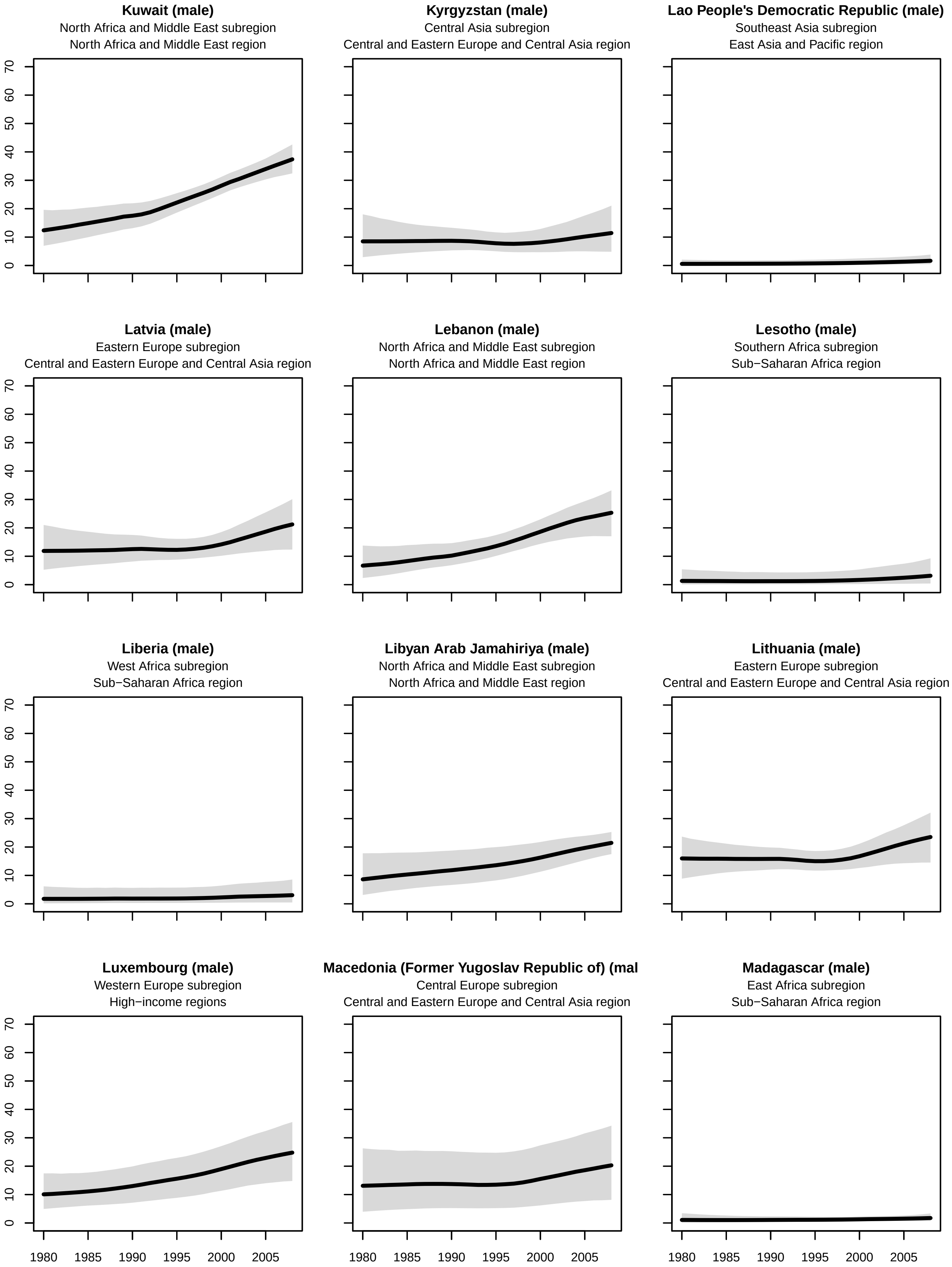
Kuwait (male)
North Africa and Middle East subregion
North Africa and Middle East region
Kyrgyzstan (male)
Central Asia subregion
Central and Eastern Europe and Central Asia region
Lao People's Democratic Republic (male)
Southeast Asia subregion
East Asia and Pacific region
Latvia (male)
Eastern Europe subregion
Central and Eastern Europe and Central Asia region
Lebanon (male)
North Africa and Middle East subregion
North Africa and Middle East region
Lesotho (male)
Southern Africa subregion
Sub−Saharan Africa region
Liberia (male)
West Africa subregion
Sub−Saharan Africa region
Libyan Arab Jamahiriya (male)
North Africa and Middle East subregion
North Africa and Middle East region
Lithuania (male)
Eastern Europe subregion
Central and Eastern Europe and Central Asia region
Luxembourg (male)
Western Europe subregion
High−income regions
Macedonia (Former Yugoslav Republic of) (mal
Central Europe subregion
Central and Eastern Europe and Central Asia region
Madagascar (male)
East Africa subregion
Sub−Saharan Africa region
0
10
20
30
40
50
60
70
1980
1985
1990
1995
2000
2005

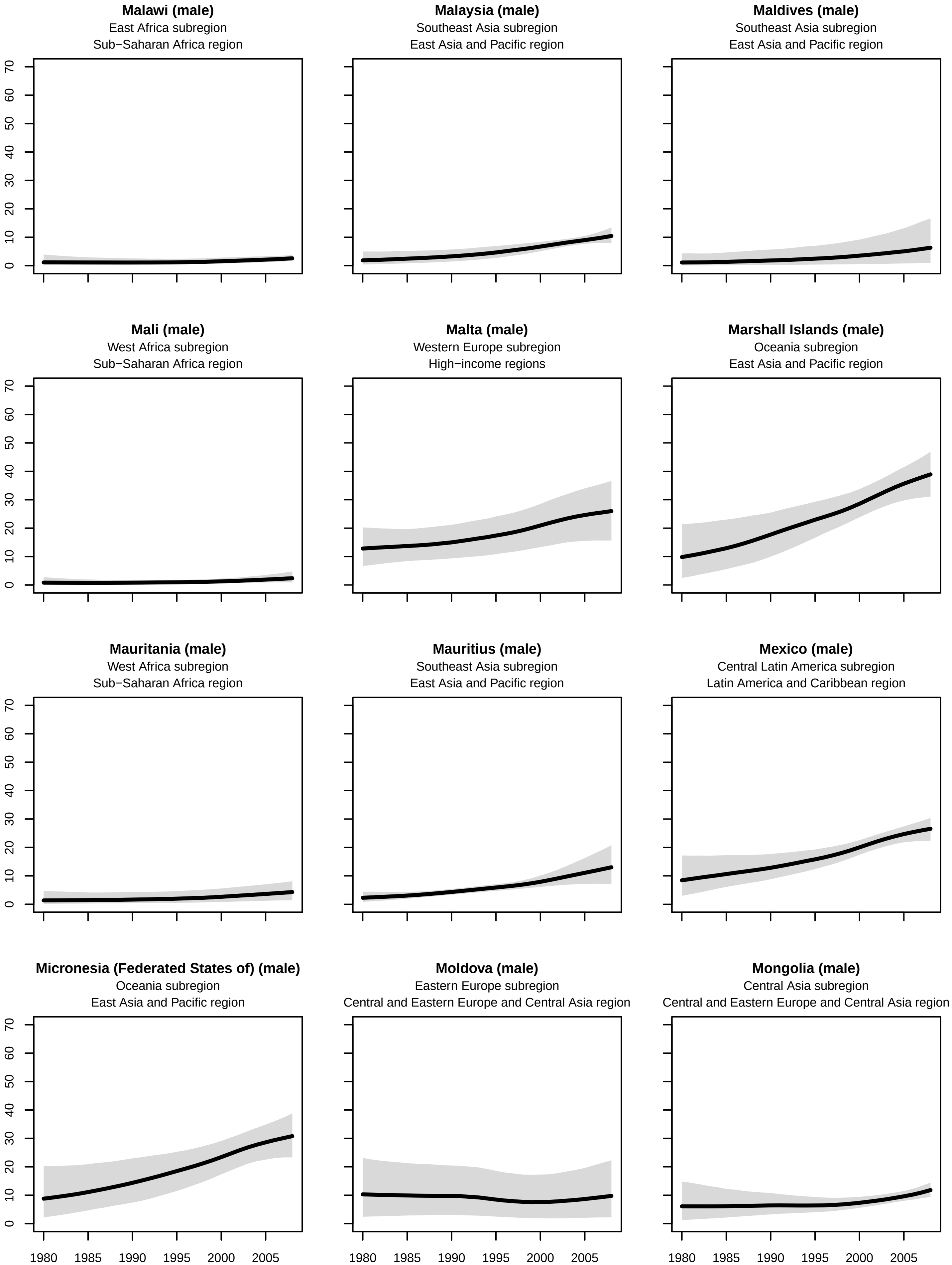
Malawi (male)
East Africa subregion
Sub−Saharan Africa region
Malaysia (male)
Southeast Asia subregion
East Asia and Pacific region
Maldives (male)
Southeast Asia subregion
East Asia and Pacific region
Mali (male)
West Africa subregion
Sub−Saharan Africa region
Malta (male)
Western Europe subregion
High−income regions
Marshall Islands (male)
Oceania subregion
East Asia and Pacific region
Mauritania (male)
West Africa subregion
Sub−Saharan Africa region
Mauritius (male)
Southeast Asia subregion
East Asia and Pacific region
Mexico (male)
Central Latin America subregion
Latin America and Caribbean region
Micronesia (Federated States of) (male)
Oceania subregion
East Asia and Pacific region
Moldova (male)
Eastern Europe subregion
Central and Eastern Europe and Central Asia region
Mongolia (male)
Central Asia subregion
Central and Eastern Europe and Central Asia region
0
10
20
30
40
50
60
70
1980
1985
1990
1995
2000
2005

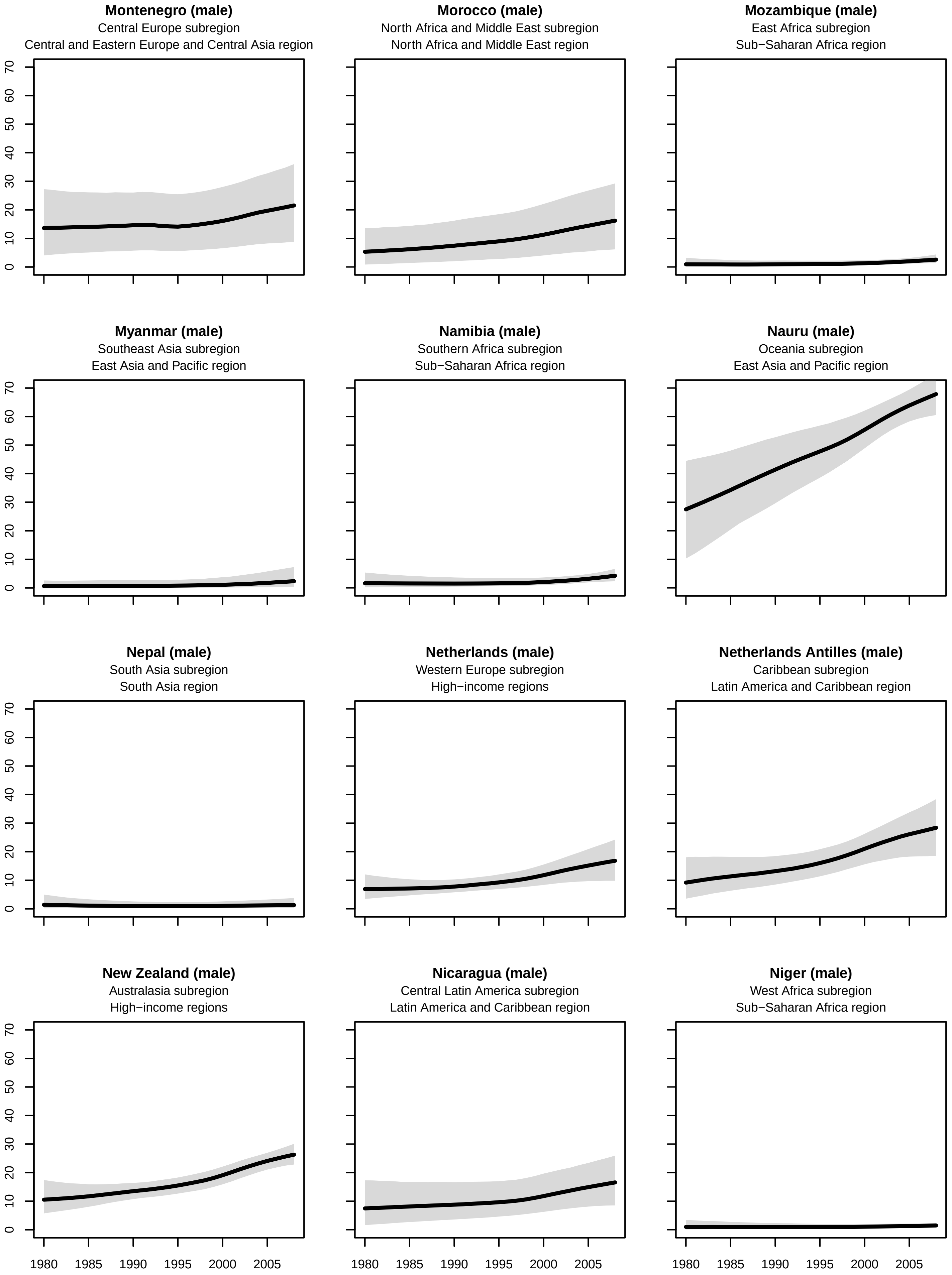
Montenegro (male)
Central Europe subregion
Central and Eastern Europe and Central Asia region
Morocco (male)
North Africa and Middle East subregion
North Africa and Middle East region
Mozambique (male)
East Africa subregion
Sub−Saharan Africa region
Myanmar (male)
Southeast Asia subregion
East Asia and Pacific region
Namibia (male)
Southern Africa subregion
Sub−Saharan Africa region
Nauru (male)
Oceania subregion
East Asia and Pacific region
Nepal (male)
South Asia subregion
South Asia region
Netherlands (male)
Western Europe subregion
High−income regions
Netherlands Antilles (male)
Caribbean subregion
Latin America and Caribbean region
New Zealand (male)
Australasia subregion
High−income regions
Nicaragua (male)
Central Latin America subregion
Latin America and Caribbean region
Niger (male)
West Africa subregion
Sub−Saharan Africa region
0 10 20 30 40 50 60 70
1980 1985 1990 1995 2000 2005

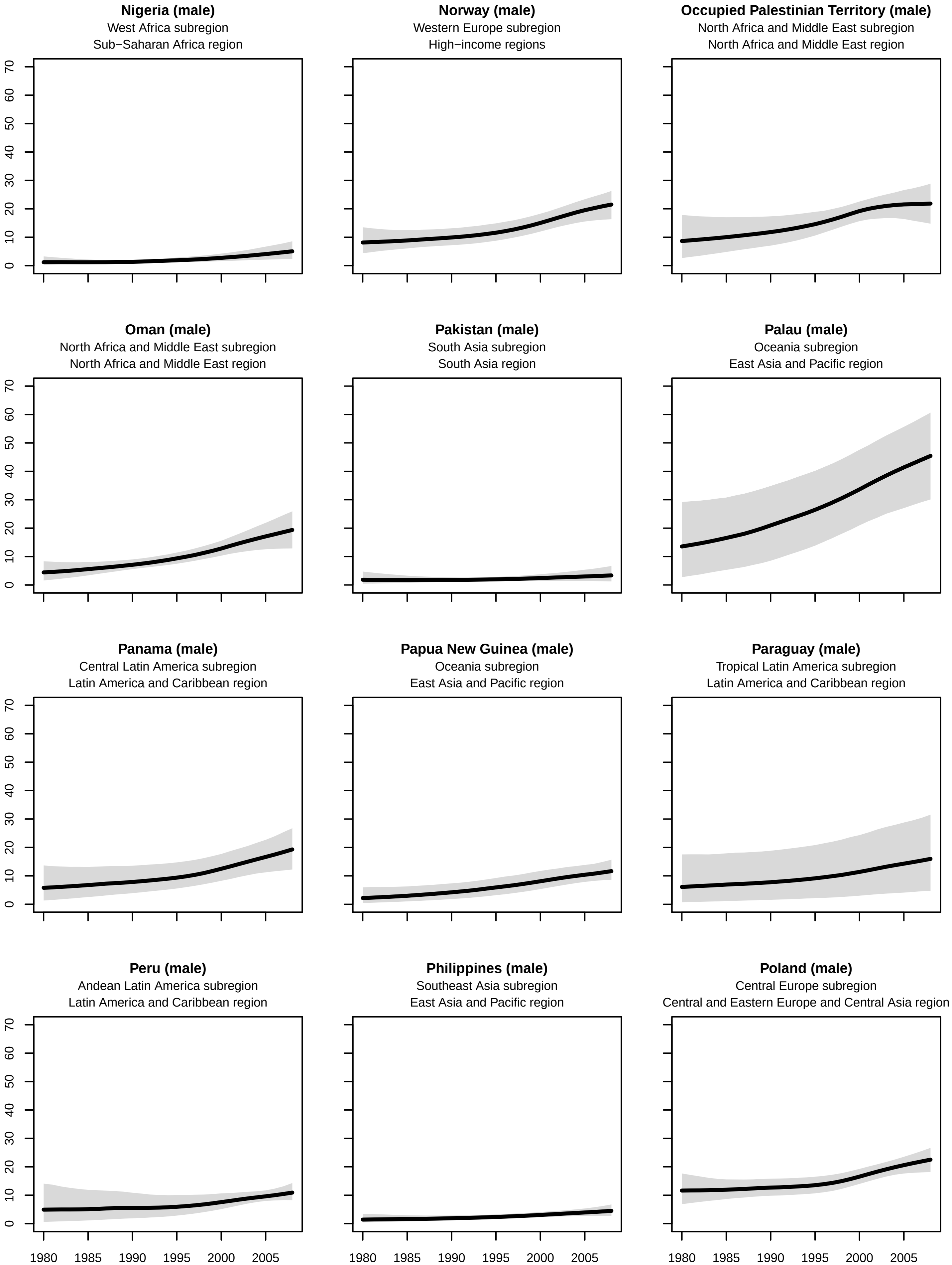

Nigeria (male)
West Africa subregion
Sub−Saharan Africa region
Norway (male)
Western Europe subregion
High−income regions
Occupied Palestinian Territory (male)
North Africa and Middle East subregion
North Africa and Middle East region
Oman (male)
North Africa and Middle East subregion
North Africa and Middle East region
Pakistan (male)
South Asia subregion
South Asia region
Palau (male)
Oceania subregion
East Asia and Pacific region
Panama (male)
Central Latin America subregion
Latin America and Caribbean region
Papua New Guinea (male)
Oceania subregion
East Asia and Pacific region
Paraguay (male)
Tropical Latin America subregion
Latin America and Caribbean region
Peru (male)
Andean Latin America subregion
Latin America and Caribbean region
Philippines (male)
Southeast Asia subregion
East Asia and Pacific region
Poland (male)
Central Europe subregion
Central and Eastern Europe and Central Asia region
0
10
20
30
40
50
60
70
1980
1985
1990
1995
2000
2005

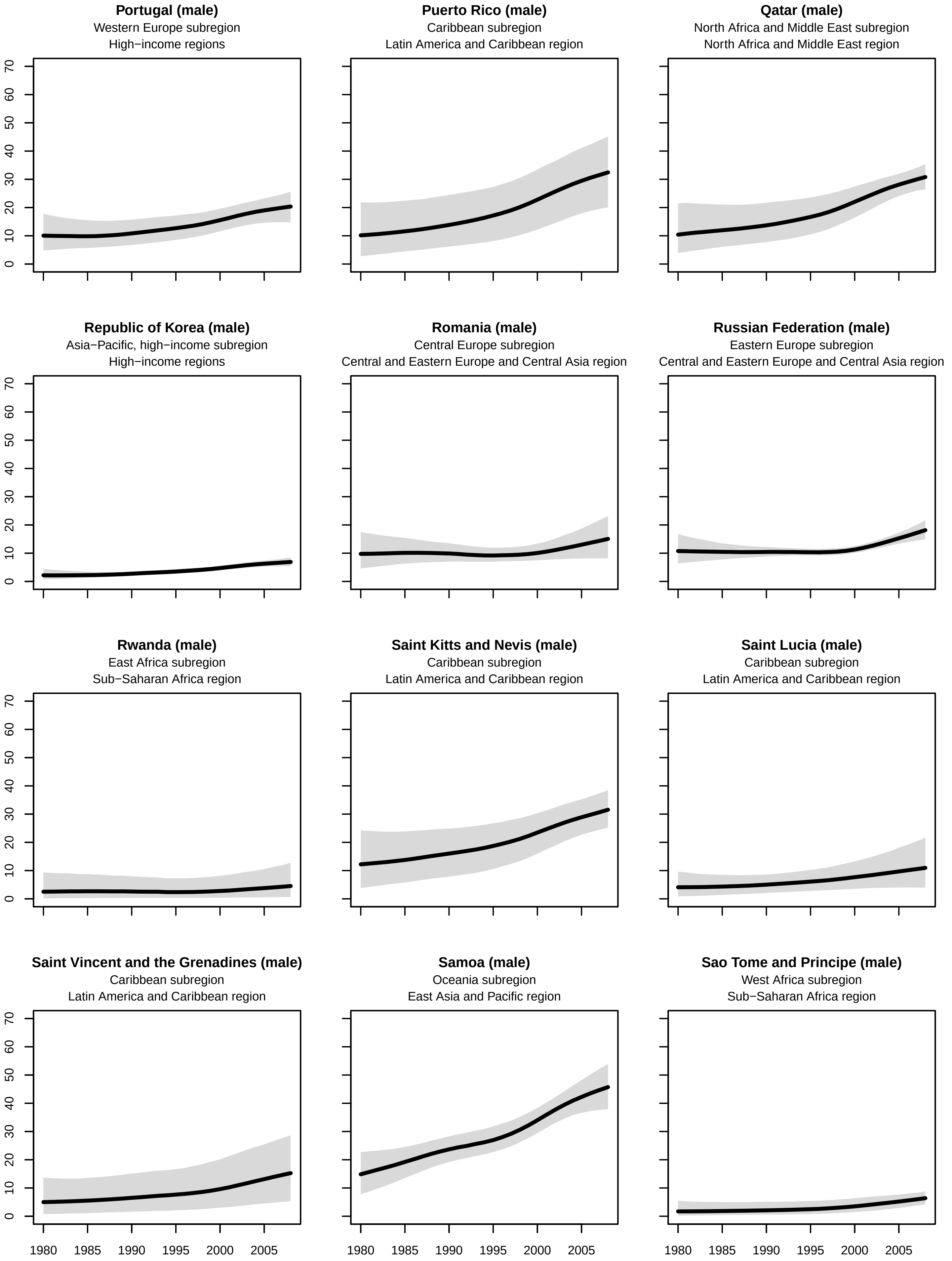
Portugal (male)
Western Europe subregion
High−income regions
Puerto Rico (male)
Caribbean subregion
Latin America and Caribbean region
Qatar (male)
North Africa and Middle East subregion
North Africa and Middle East region
Republic of Korea (male)
Asia−Pacific, high−income subregion
High−income regions
Romania (male)
Central Europe subregion
Central and Eastern Europe and Central Asia region
Russian Federation (male)
Eastern Europe subregion
Central and Eastern Europe and Central Asia region
Rwanda (male)
East Africa subregion
Sub−Saharan Africa region
Saint Kitts and Nevis (male)
Caribbean subregion
Latin America and Caribbean region
Saint Lucia (male)
Caribbean subregion
Latin America and Caribbean region
Saint Vincent and the Grenadines (male)
Caribbean subregion
Latin America and Caribbean region
Samoa (male)
Oceania subregion
East Asia and Pacific region
Sao Tome and Principe (male)
West Africa subregion
Sub−Saharan Africa region
0
10
20
30
40
50
60
70
1980
1985
1990
1995
2000
2005

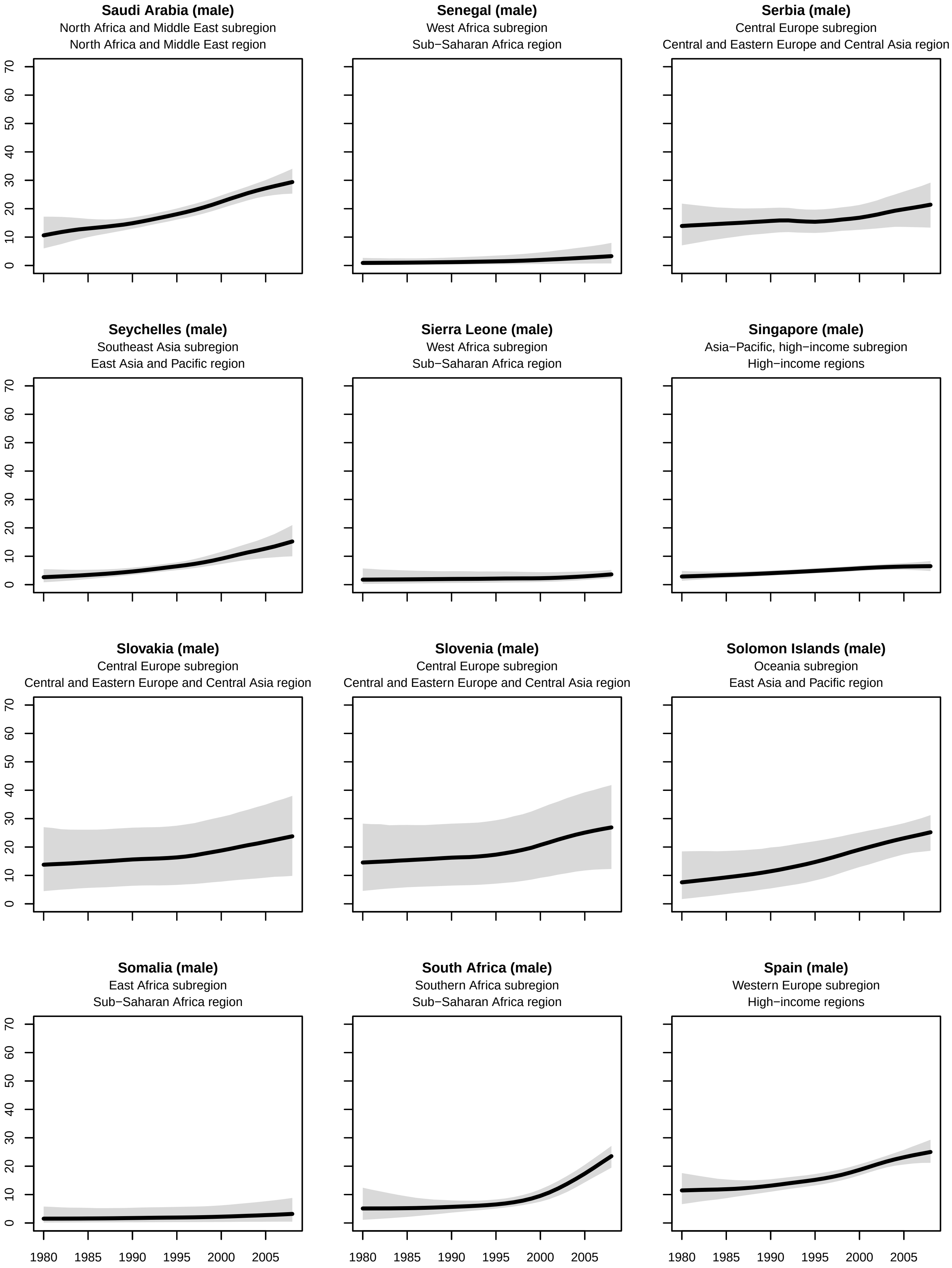
Saudi Arabia (male)
North Africa and Middle East subregion
North Africa and Middle East region
Senegal (male)
West Africa subregion
Sub−Saharan Africa region
Serbia (male)
Central Europe subregion
Central and Eastern Europe and Central Asia region
Seychelles (male)
Southeast Asia subregion
East Asia and Pacific region
Sierra Leone (male)
West Africa subregion
Sub−Saharan Africa region
Singapore (male)
Asia−Pacific, high−income subregion
High−income regions
Slovakia (male)
Central Europe subregion
Central and Eastern Europe and Central Asia region
Slovenia (male)
Central Europe subregion
Central and Eastern Europe and Central Asia region
Solomon Islands (male)
Oceania subregion
East Asia and Pacific region
Somalia (male)
East Africa subregion
Sub−Saharan Africa region
South Africa (male)
Southern Africa subregion
Sub−Saharan Africa region
Spain (male)
Western Europe subregion
High−income regions
0 10 20 30 40 50 60 70
1980 1985 1990 1995 2000 2005

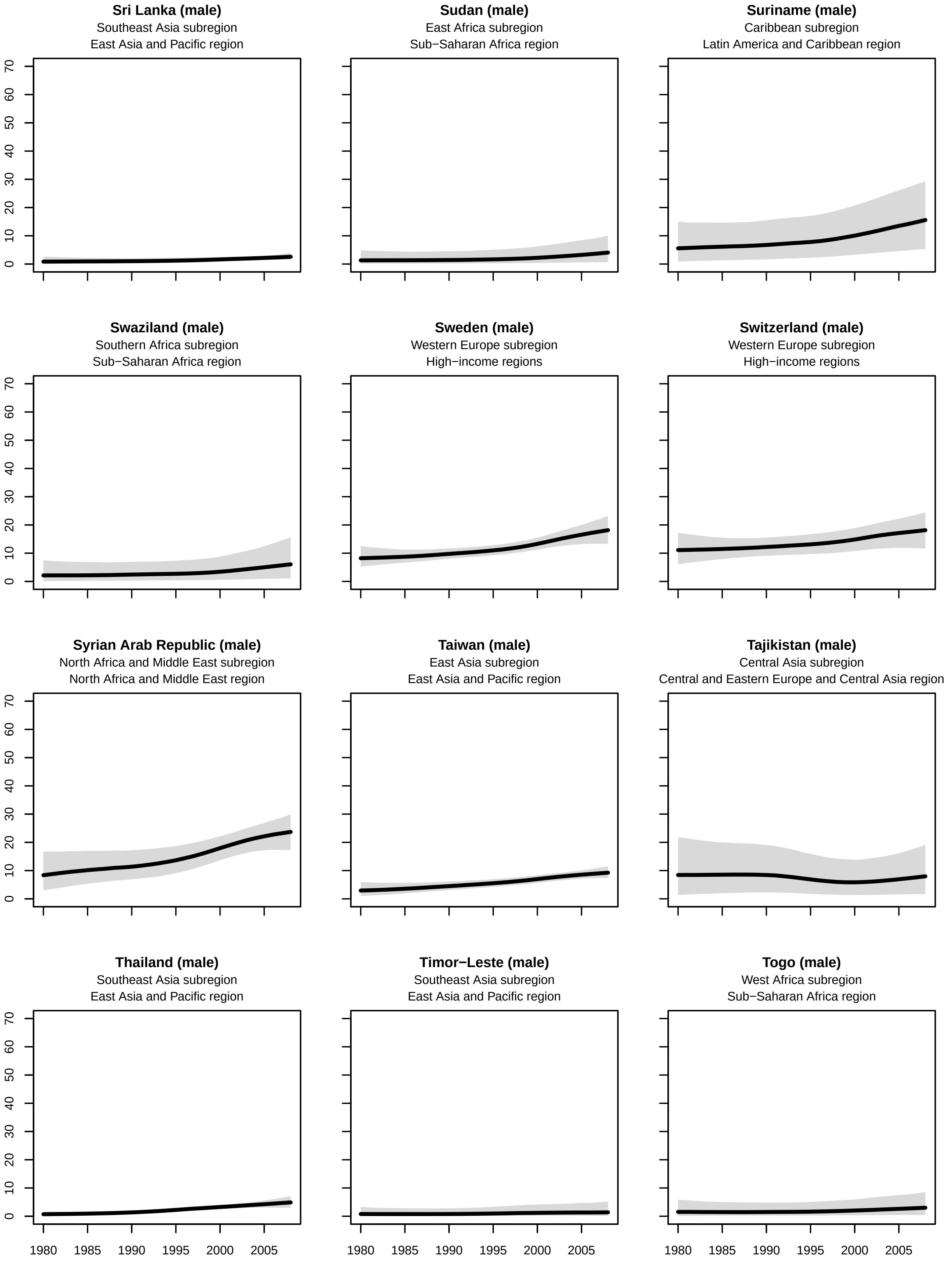

Sri Lanka (male)
Southeast Asia subregion
East Asia and Pacific region
Sudan (male)
East Africa subregion
Sub−Saharan Africa region
Suriname (male)
Caribbean subregion
Latin America and Caribbean region
Swaziland (male)
Southern Africa subregion
Sub−Saharan Africa region
Sweden (male)
Western Europe subregion
High−income regions
Switzerland (male)
Western Europe subregion
High−income regions
Syrian Arab Republic (male)
North Africa and Middle East subregion
North Africa and Middle East region
Taiwan (male)
East Asia subregion
East Asia and Pacific region
Tajikistan (male)
Central Asia subregion
Central and Eastern Europe and Central Asia region
Thailand (male)
Southeast Asia subregion
East Asia and Pacific region
Timor−Leste (male)
Southeast Asia subregion
East Asia and Pacific region
Togo (male)
West Africa subregion
Sub−Saharan Africa region
0 10 20 30 40 50 60 70
1980 1985 1990 1995 2000 2005

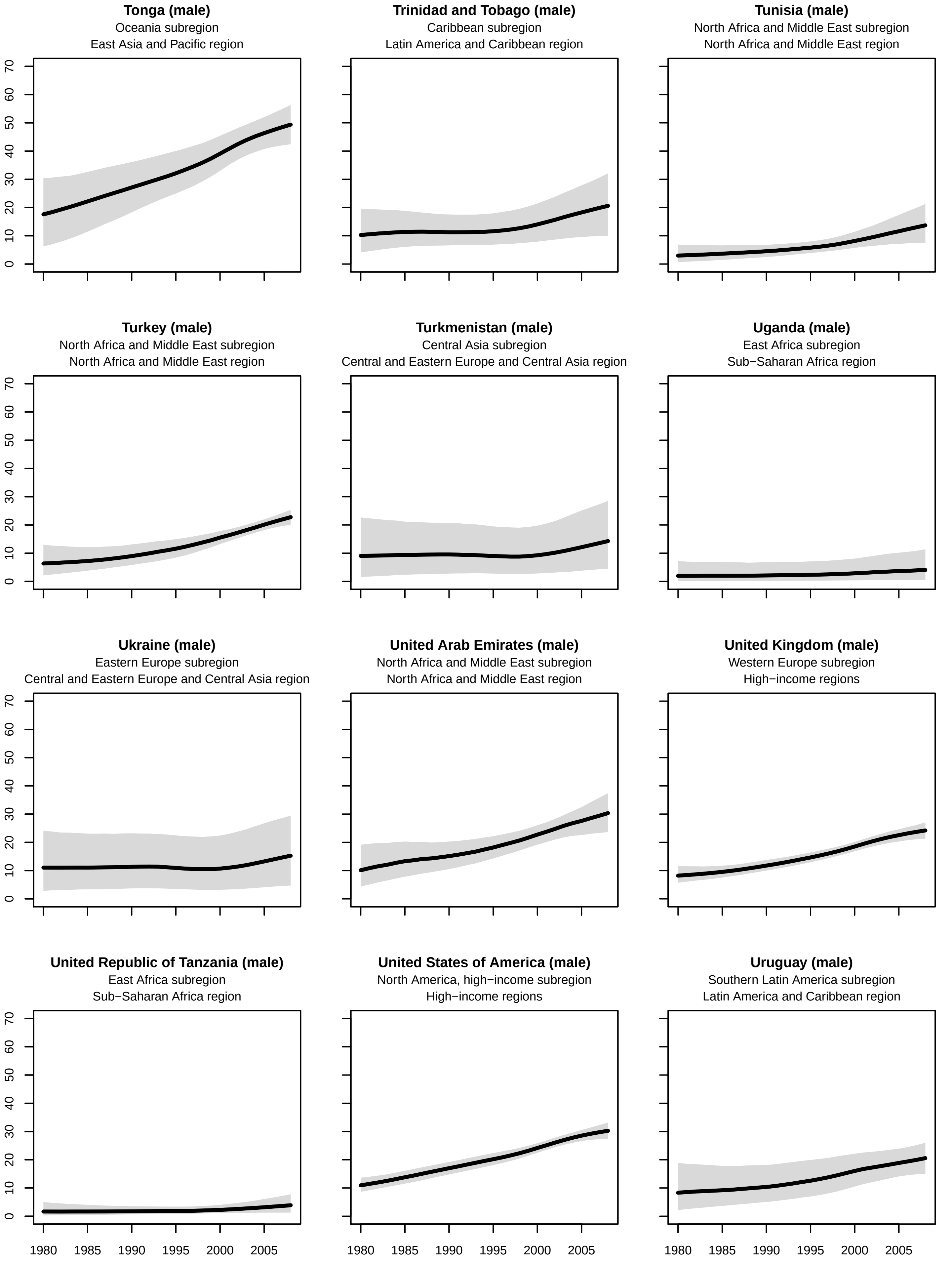
Tonga (male)
Oceania subregion
East Asia and Pacific region
Trinidad and Tobago (male)
Caribbean subregion
Latin America and Caribbean region
Tunisia (male)
North Africa and Middle East subregion
North Africa and Middle East region
Turkey (male)
North Africa and Middle East subregion
North Africa and Middle East region
Turkmenistan (male)
Central Asia subregion
Central and Eastern Europe and Central Asia region
Uganda (male)
East Africa subregion
Sub−Saharan Africa region
Ukraine (male)
Eastern Europe subregion
Central and Eastern Europe and Central Asia region
United Arab Emirates (male)
North Africa and Middle East subregion
North Africa and Middle East region
United Kingdom (male)
Western Europe subregion
High−income regions
United Republic of Tanzania (male)
East Africa subregion
Sub−Saharan Africa region
United States of America (male)
North America, high−income subregion
High−income regions
Uruguay (male)
Southern Latin America subregion
Latin America and Caribbean region
0
10
20
30
40
50
60
70
1980
1985
1990
1995
2000
2005

Uzbekistan (male)
Central Asia subregion
Central and Eastern Europe and Central Asia region

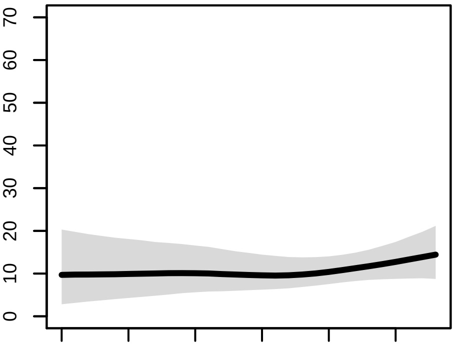

Vanuatu (male)
Oceania subregion
East Asia and Pacific region

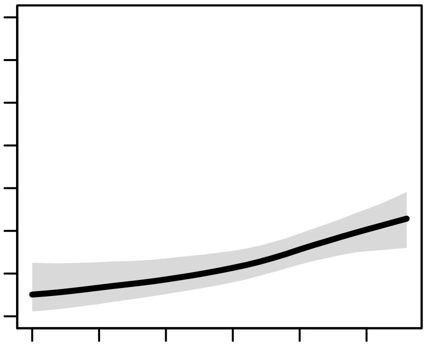

Venezuela (Bolivarian Republic of) (male)
Central Latin America subregion
Latin America and Caribbean region

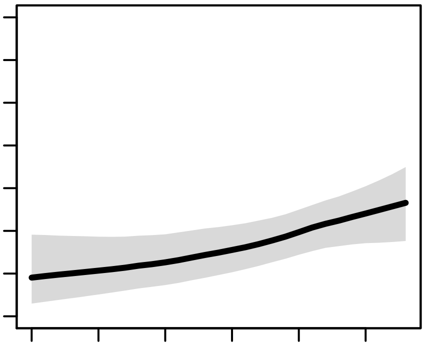

Viet Nam (male)
Southeast Asia subregion
East Asia and Pacific region

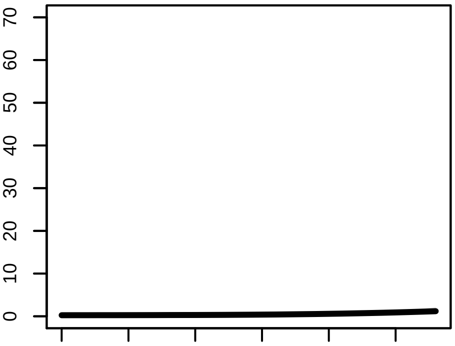

Yemen (male)
North Africa and Middle East subregion
North Africa and Middle East region

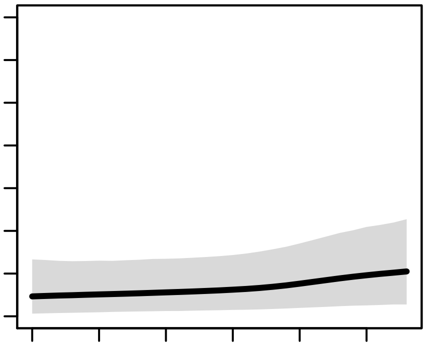

Zambia (male)
East Africa subregion
Sub−Saharan Africa region

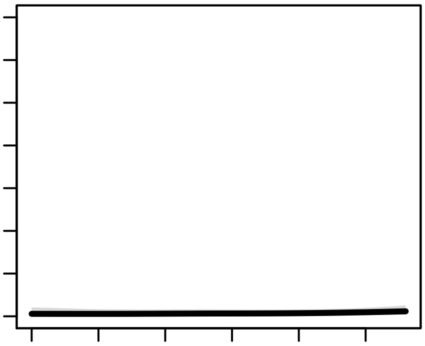

Zimbabwe (male)
Southern Africa subregion
Sub−Saharan Africa region

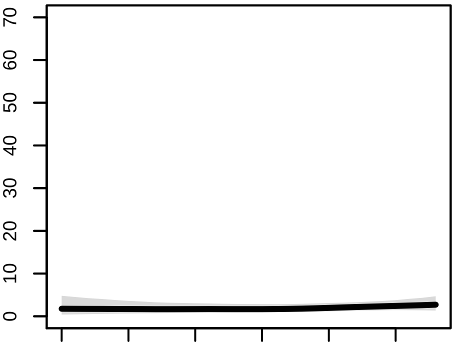

Supplement: Additional file 3 — Trends in age-standardized mean overweight (BMI ≥ 25 kg/m2) by country between 1980 and 2008, adults ≥ 20 years. The solid line represents the posterior mean estimate and the shaded area the 95% uncertainty interval. [file 1478-7954-10-22-S3.pdf]
